# Network-based virus-host interaction prediction with application to SARS-CoV-2

**DOI:** 10.1016/j.patter.2021.100242

**Published:** 2021-03-29

**Authors:** Hangyu Du, Feng Chen, Hongfu Liu, Pengyu Hong

**Affiliations:** 1 Department of Computer Science, Brandeis University, Waltham, MA 02453, USA

**Keywords:** coronavirus, COVID-19, SARS-CoV-2, machine learning, interaction prediction, protein-protein interaction, virus-host interaction network

## Abstract

COVID-19, caused by Severe Acute Respiratory Syndrome Coronavirus 2 (SARS-CoV-2), has quickly become a global health crisis since the first report of infection in December of 2019. However, the infection spectrum of SARS-CoV-2 and its comprehensive protein-level interactions with hosts remain unclear. There is a massive amount of underutilized data and knowledge about RNA viruses highly relevant to SARS-CoV-2 and proteins of their hosts. More in-depth and more comprehensive analyses of that knowledge and data can shed new light on the molecular mechanisms underlying the COVID-19 pandemic and reveal potential risks. In this work, we constructed a multi-layer virus-host interaction network to incorporate these data and knowledge. We developed a machine-learning-based method to predict virus-host interactions at both protein and organism levels. Our approach revealed five potential infection targets of SARS-CoV-2 and 19 highly possible interactions between SARS-CoV-2 proteins and human proteins in the innate immune pathway.

## Highlights

• We built a virus-host interaction network with 7 human coronaviruses and 17 hosts
• We developed an ML-based method to predict protein- and organism-level interactions
• We revealed five potential infection targets of SARS-CoV-2
• We predicted 19 highly possible interactions between SARS-CoV-2 and human proteins

## The bigger picture

SARS-CoV-2, a novel single-stranded RNA coronavirus causing COVID-19, is mounting an unprecedented threat against our society and the world. Although tremendous efforts have been devoted into SARS-CoV-2 research, most of them either focused on a few proteins or only provided high-level overviews. Deeper and more comprehensive analyses are needed to shed new light onto the molecular mechanisms underlying the COVID-19 pandemic. Moreover, there is a massive amount of data and knowledge about highly relevant RNA viruses which have yet to be fully utilized.

In this work, we constructed a multi-layer virus-host interaction network to incorporate these data and knowledge. We developed a machine-learning-based method to predict virus-host interactions at both protein and organism levels. Our approach revealed five potential infection targets of SARS-CoV-2 and 19 highly possible interactions between SARS-CoV-2 proteins and human proteins in the innate immune pathway.

## Teaser

Given a new virus, our method can utilize existing knowledge and data about other highly relevant viruses to predict multi-scale interactions between the new virus and potential hosts.

## Introduction

Severe Acute Respiratory Syndrome Coronavirus-2 (SARS-CoV-2), a novel virus causing the COVID-19 disease, was first reported in Wuhan, China, in December of 2019. Since then, it has quickly become a global health crisis^1^ with over 50 million people infected and over 1,250,000 deaths across 200 countries by November 2020.^2^ The impact of SARS-CoV-2 has significantly surpassed previous outbreaks of coronaviruses, such as Severe Acute Respiratory Syndrome Coronavirus (SARS-CoV) in 2003 and the Middle East Respiratory Syndrome Coronavirus (MERS-CoV) in 2012. Besides humans, SARS-CoV-2 has been confirmed to infect several other mammals closely related to human activities, including dogs,^3^ cats,^4^ tigers,^5^ rats,^6^ and golden Syrian hamsters.^7^ Also, there is a high possibility for infected animals to transmit and spread the virus to humans.^8^ It is important to identify a comprehensive set of such mammals because they can potentially serve as covert means to exacerbate the spread of COVID-19. Moreover, identifying interactions between SARS-CoV-2 proteins and host proteins can deepen our understanding of the viral invasion processes and may help design treatments and vaccines. In general, we want to promptly achieve the above two goals for new zoonotic viruses, which we believe can be done by leveraging the knowledge and data about known viruses highly relevant to the new ones.

**Figure 1:**
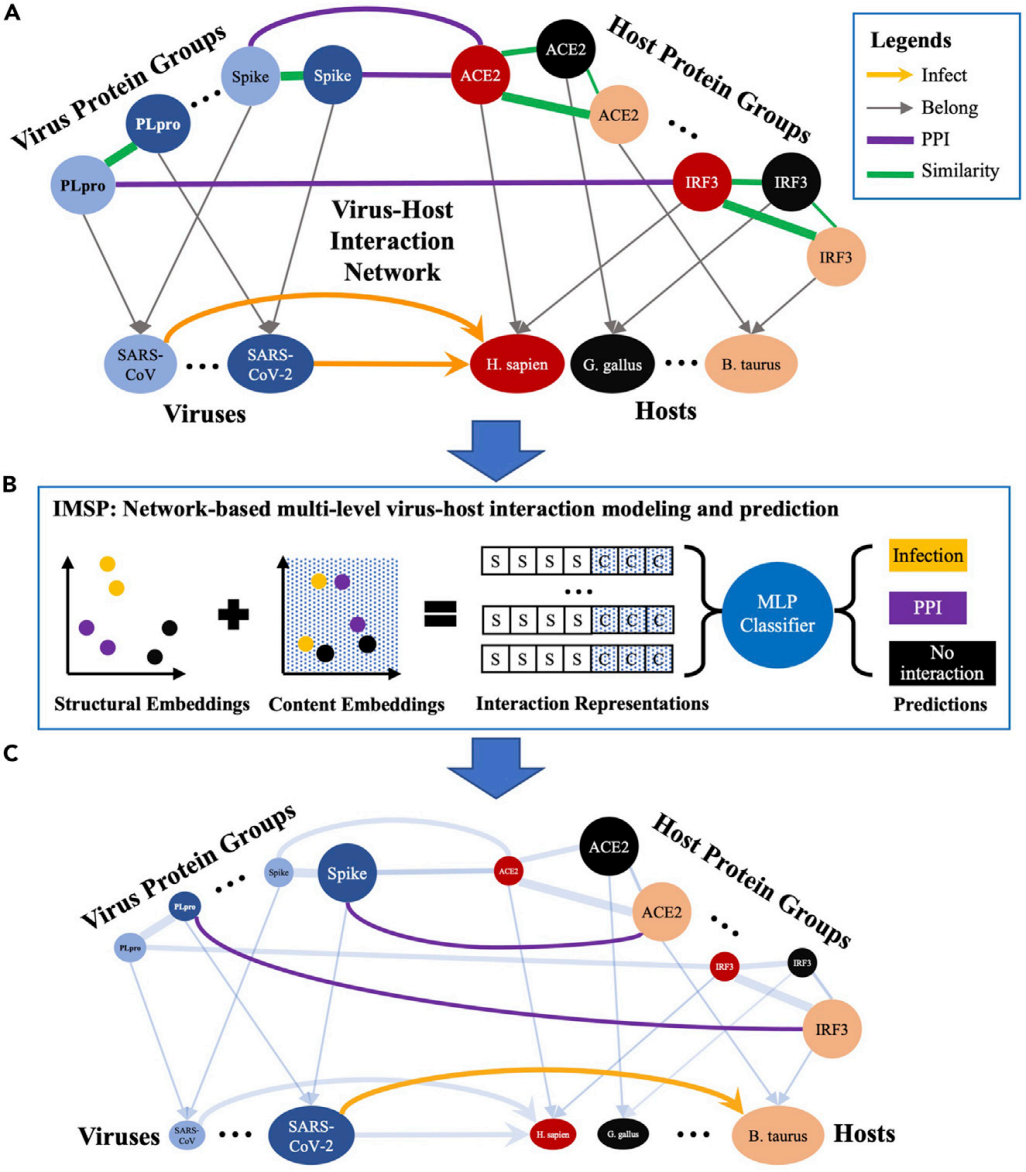
Infection mechanism and spectrum prediction (A) The virus-host interaction network. Nodes represent proteins, viruses, and hosts; edges represent relationships (i.e., PPI, infection, protein-homolog similarity, and organism-protein belonging). The color of a node indicates its organism. The thickness of a protein-homolog similarity edge indicates its level of similarity. For the full network, refer to the viral entry graph (Figure S3), interferon signaling pathway graph (Figure S4), and infection graph (Figure S5). (B) IMSP learns a representation for each potential edge, which contains a structural embedding and a content embedding. The structural embedding captures the local structural features of an edge. The content embedding captures the attributes that reveal biological aspects of an edge. The representation of each edge is derived by concatenating its structural and content embeddings, where S stands for a structural embedding element and C stands for a content embedding element. A Multi-layer Perceptron (MLP) is trained to take the edge representations as input and reports negative (non-connected) edges whose corresponding edge representations are classified as infection or PPI. Note that no-interaction is also a potential class for the classification task. See experimental procedures for calculation of the structural and content embeddings. (C) Exemplar predicted edges are highlighted and colored accordingly to their types. Existing edges are dimmed.

The research community has accumulated a great deal of knowledge about several other human coronaviruses (including SARS-CoV,^9^, ^10^, ^11^, ^12^, ^13^, ^14^, ^15^, ^16^ HCoV-HKU1,^14^ HCoV-OC43,^17^^,^^18^ HCoV-NL63,^19^ and MERS-CoV)^20^, ^21^, ^22^, ^23^, ^24^ and has collected a large amount of data about them. For example, it was shown that human angiotensin-converting enzyme 2 (ACE2) was the primary host receptor used by the S protein (S-protein) of SARS-CoV-2 for the virus to gain entry into human cells^25^ (Figure S1). ACE2 is also the host receptor used by SARS-CoV^13^ and HCoV-NL63.^19^ The S-protein of SARS-CoV-2 binds significantly tighter to ACE2 than its counterpart in SARS-CoV.^26^ After the virus enters host cells, interferon-stimulated genes are essential for a host to defend against viral infection (Figure S2). This knowledge and data can be utilized to investigate the infection spectrum of SARS-CoV-2 and its interactions with hosts at the protein level. Using this information, we have built a virus-host interaction network of 7 viruses and 17 hosts that summarizes the existing protein-protein interaction (PPI) and infection relationships among them (Figure 1A; for more details, see Figures S3–S5 and Tables S1, S2, S3, and S4).

We have developed a network-based multi-level virus-host interaction modeling and prediction, termed infection mechanism and spectrum prediction (IMSP) (Figure 1B; for details, see experimental procedures), which uses machine-learning techniques to learn from the constructed virus-host interaction network and predict novel virus-host interactions at both the protein (i.e., Mechanism) and organism (i.e., Spectrum) levels. IMSP predicts that the SARS-CoV-2 S-protein can bind well with ACE2 receptors in five mammalian hosts, which have not been reported. Among those hosts, five are predicted to have high risks of being infected by SARS-CoV-2. Moreover, IMSP identifies 19 new interactions between SARS-CoV-2 proteins and human proteins in the innate immune pathway. To our best knowledge, our work is the first to apply machine-learning techniques for predicting virus-host interactions at both protein and organism levels. Previous works^27^^,^^28^ only focused on the relationships between SARS-CoV-2 proteins and human proteins and ignored other hosts that might be infected by SARS-CoV-2.

## Results

Here we explain the structure of our virus-host interaction network, highlight the predicted interactions of SARS-CoV-2, and present the link prediction performance evaluation of our model IMSP. We built our network with two layers (an organism layer and a protein layer). The organism layer consisted of 7 human coronaviruses and 17 mammalian hosts. Those hosts are either close to human activities or proven to be infected by some human coronaviruses in our network. The protein layer contained 10 virus proteins and 13 host proteins. The proteins were selected based on two primary considerations: proteins involved in viral entry and the interferon (IFN) signaling pathway, both of which are critical to a successful virus infection. The virus needs to enter the host cells through the receptors on the membrane, and the binding ability between the S-protein of the virus and the host receptor determines the success of such viral entry. The suppression ability on the IFN signaling pathway of the virus negatively affects the efficiency and the effectiveness of the response of the innate immune system, which would allow the virus to rapidly replicate and spread among cells. IMSP performed a network-based representation learning to integrate information about virus-host infections, PPIs, organism-protein belongings, and similarities between protein homologs. This produced comprehensive representations and a neural-network-based classifier for accurately predicting novel viral infection and interactions between virus proteins and host proteins.

### SARS-CoV-2-host multiple-type interaction predictions

We applied IMSP on SARS-CoV-2 and six other human coronaviruses to obtain high-confidence predictions of PPIs and infections. Figure S1 shows the mechanism of the binding of S-proteins and host receptor ACE2. Figure S2 shows the interactions between virus proteins and host proteins involved in the IFN pathway. Figure S3 shows the S-protein binding subnetwork. Figure S4 shows the innate immune pathway subnetwork. Figure S5 shows the organism layer. Tables S1 and S2 show the complete node and linkage information of the virus-host network. All infection predictions are shown in Table S3, and PPI predictions are presented in Table S4.

### SARS-CoV-2 S-protein binding predictions

**Figure 2:**
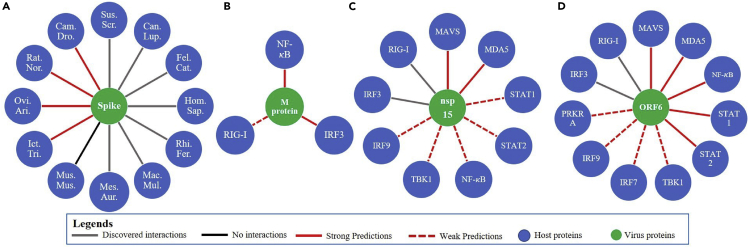
PPIs prediction for SARS-CoV-2 (A) The known and predicted bindings between the S-protein in SARS-CoV-2 and ACE2 in mammalian hosts. Host names are displayed in their abbreviation form: Hom.Sap., *Homo sapiens*; Mus.Mus., *Mus musculus*; Fel.Cat., *Felis catus*; Can.Lup., *Canis lupus familiaris*; Ovi.Ari., *Ovis aries*; Rat.Nor., *Rattus norvegicus*; Mac.Mul., *Macaca mulatta*; Rhi.Fer., *Rhinolophus ferrumequinum*; Mes.Aur., *Mesocricetus auratus*; Bos.Tau., *Bos taurus*; Ict.Tri., *Ictidomys tridecemlineatus*; Cam.Dro., *Camelus dromedarius*; Sus.Scr., *Sus scrofa domesticus*. (B–D) The known and predicted interactions of M protein (B), nsp15 (C), and ORF6 (D) in SARS-CoV-2 with proteins in the human IFN signaling pathway that contribute to IFN signaling pathway suppression.

The binding ability of the S-protein of SARS-CoV-2 with the host ACE2 receptors is a key factor deciding the infection capability of SARS-CoV-2. IMSP predicted that the S-protein of SARS-CoV-2 could have a high probability of binding well with the ACE2 receptors in rats, sheep, camels, and squirrels (Figure 2A).

Rats were recognized to be susceptible to several other human coronaviruses, such as SARS-CoV,^9^ MERS-CoV,^29^ HCoV-OC43,^18^ and HCoV-HKU1.^30^^,^^31^ It is highly possible that rats could still be the potential host for SARS-CoV-2.

The overall similarity of ACE2 for the squirrel, sheep, and camel is 91.82%, 90.81%, and 92.42%, respectively compared with human ACE2. These predictions still require more practical research to determine the binding affinity between the S-protein of SARS-CoV-2 with ACE2s on these mammals. It was shown that ACE2 could tolerate up to seven amino acid changes out of 20 critical ones that contact with the S-protein without losing the functionality as the target receptor^32^ for SARS-CoV-2. This means that sequence similarity might not be the only factor that influences the binding affinity between the ACE2 receptor and the S-protein of SARS-CoV-2.

### SARS-CoV-2 and human interferon pathway interactome prediction

The IFN pathway plays a critical role in the human immune response. After the virus infection is detected, the innate immune system will induce IFN signaling, and the expression of IFN genes will increase the cellular resistance to viral invasion. Viruses have developed various strategies to inhibit IFN signaling to facilitate successful viral invasion.^33^ SARS-CoV and MERS-CoV were studied quite comprehensively in terms of counteracting the IFN signaling responses compared with SARS-CoV-2. From IMSP, 19 interactions between SARS-CoV-2 proteins and human proteins in the innate immune pathway were identified, shown in Figures 2B–2D. These PPIs had a high probability of playing crucial roles in the suppression of the innate immune system response of the host.

Membrane (M) protein not only serves as the protein in virus to bind to all other structural proteins^34^ but also is found to inhibit IFN production in SARS-CoV^35^ and MERS-CoV.^24^ From IMSP prediction, it was highly possible that M protein in SARS-CoV-2 could interact with nuclear factor kappa-light-chain-enhancer of activated B (NF-κB), interferon regulatory factor 3 (IRF3), and retinoic acid-inducible gene I (RIG-I).

Open reading frame protein 6 (ORF6) and non-structural protein 15 (nsp15) in SARS-CoV-2 were discovered to be crucial viral IFN antagonists of SARS-CoV-2. From previous research, we knew that these two proteins inhibit the localization of IRF3 by interacting with RIG-I.^36^ A similar function was found for ORF6 in SARS-CoV.^37^ ORF6 and nsp15 in SARS-CoV were proved to interact with signal transducer and activator of transcription 1 (STAT1) and STAT2.^38^ From predictions made by IMSP (shown in Figures 2C and 2D), ORF6 and nsp15 in SARS-CoV-2 were suggested to have potential interactions with melanoma differentiation-associated protein 5 (MDA5), mitochondrial anti-viral-signaling protein (MAVS), STAT1, STAT2, NF-κB, IRF9, and TANK binding kinase 1 (TBK1). Since MAVS works as the adaptor molecule for MDA5,^39^ it is possible that a viral protein that interacts with either one of these two would also interact with the other. Besides these, ORF6 was also predicted to interact with protein kinase interferon-inducible double-stranded RNA-dependent activator (PRKRA) and IRF7. As nsp15 and ORF6 both function in nuclear transport machinery after viral entry,^27^ it is reasonable that, for these two proteins, similar interactions with innate immune pathways are predicted. Careful experiments should be conducted to identify the impact of nsp15 and ORF6 on the innate immune system.

### SARS-CoV-2 infection prediction

**Figure 3:**
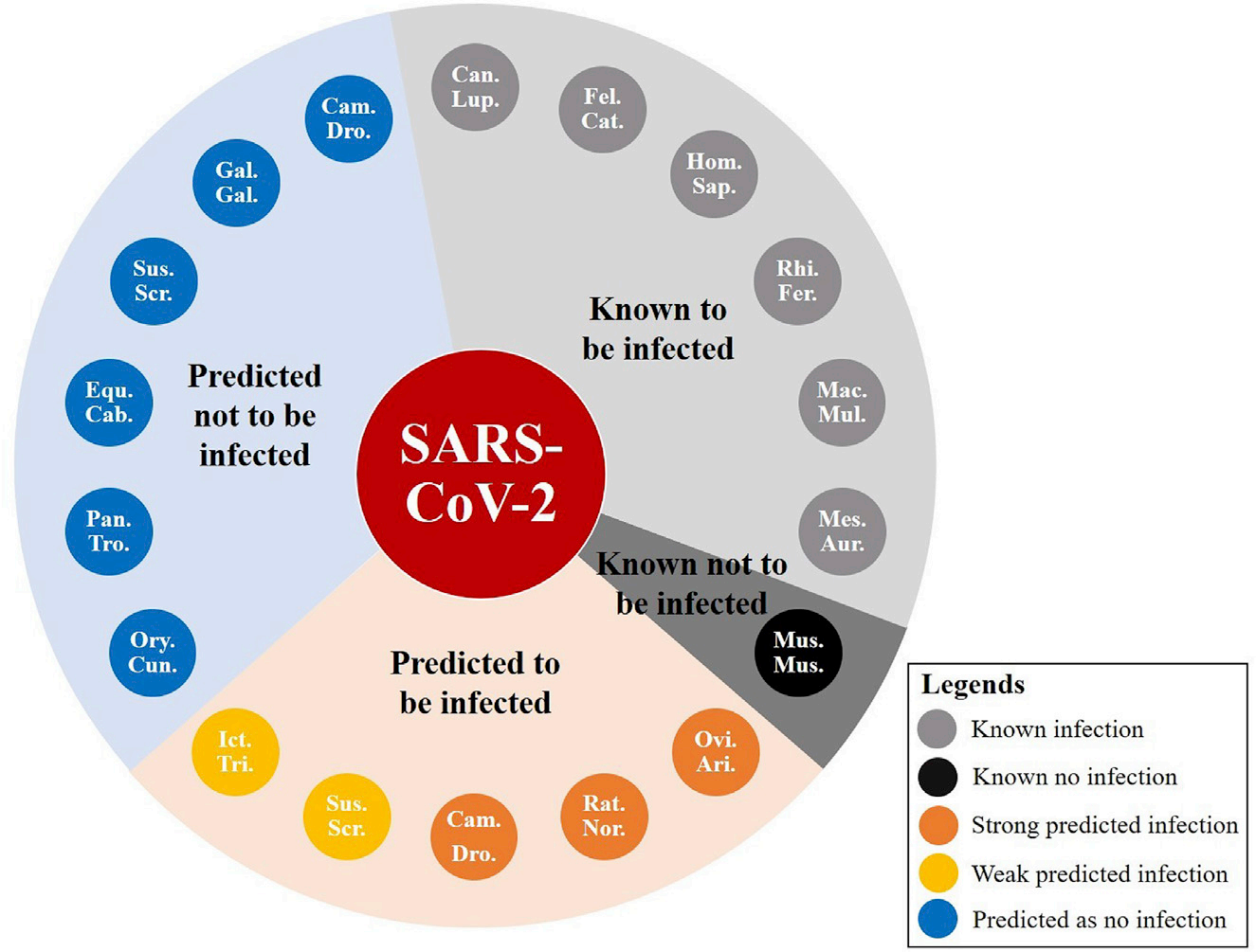
Infection prediction for SARS-CoV-2 This figure shows all 17 mammalian hosts in our network and their infection relationships with SARS-CoV-2.

Based on both the protein-level and organism-level interaction predictions, we concluded five highly possible infection predictions for SARS-CoV-2. These mammals were predicted to be susceptible to SARS-CoV-2 in the organism layer. They were also proved or predicted to have a successful spike-receptor binding between the S-protein of SARS-CoV-2 and their own ACE2 receptors. As shown in Figure 3, these animals included rats, sheep, camels, swine, and squirrels.

Rat was identified as a host for all beta-coronaviruses: SASR-CoV,^9^ MERS-CoV,^29^ HCoV-OC43,^18^ and HCoV-HKU1.^30^^,^^31^ SARS-CoV-2 also falls into the category of beta-coronavirus,^40^ which has a high possibility of infecting rats.

Swine's ACE2 was identified to be able to bind with the S-protein of SARS-CoV-2,^41^ and our model predicted that swine could be successfully infected after the receptor binding. This is also supported by recent research on swine.^42^

Camels are hosts for MERS-CoV.^22^ This means that camels can also be hosts for other coronaviruses. Camels, along with sheep and squirrels, are closely related to the human living environment or daily diet. They could be potential mammalian hosts that again transmit the virus back to human society. The investigation of these highly possible infections could potentially help identify the transition path of the virus and further control the transmission of SARS-CoV-2 from and between mammalian hosts. Further research on these potential hosts might be crucial to social health and safety.

### Interaction prediction performance evaluation

Many machine-learning and graph-embedding methods have been developed and applied to various applications.^43^, ^44^, ^45^, ^46^, ^47^, ^48^ In this work, we compared IMSP with five other baseline models on our dataset in a 5-fold stratified cross-validation setting. The baseline models include two famous random-walk-based models (DeepWalk^43^ and Node2vec),^45^ two neural-network-based models (Large-scale Information Network Embedding [LINE]^44^ and Structural Deep Network Embedding [SDNE]),^46^ and a classical matrix-based model, Graph Factorization GF.^49^ For the stratified cross-validation experiment, we created a sampling strategy to ensure that the training subset in each cross-validation run can form a fully connected network. Such a fully connected network could ensure that our network structural embedding model embedded nodes into the same vector space. To ensure the balance of input data, we gathered negative (non-connected) edges in addition to positive (connected) edges that already existed in each fold. We sampled negative edges from two directions: known negatives (i.e., true negatives) and unknown negatives. We considered spike-receptor interactions demonstrated as nonexistent as known negatives, such as the one between the S-protein of SARS-CoV-2 and the host receptor dipeptidyl peptidase 4 (DPP4; the target host receptor of MERS-CoV). Since we still lacked a comparable amount of negative edges, we randomly selected non-connected node pairs as negative edges, which we assumed as not existing. We added these negative samples into each fold to match the number of positive samples. We then evaluated IMSP and other models under the 5-fold stratified cross-validation setting as described above. We repeated the cross-validation experiment for 30 independent runs. In each run, we generated a new 5-fold split. Finally, we performed a two-sample heteroscedastic t test at the 0.01 significance level to test the significance of our model's improvement against other models.

**Figure 4:**
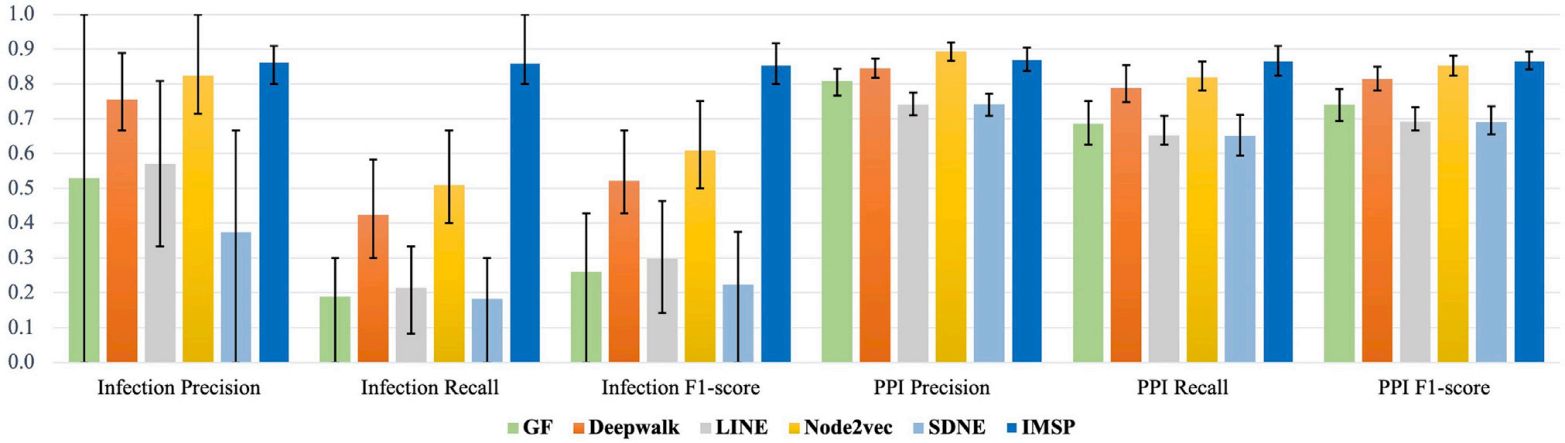
Performance on PPI and infection predictions This figure demonstrates the performance of IMSP on PPI and infection predictions in comparison with five other baseline models. The ocean-blue columns represent the performance of IMSP derived from the average of 30 independent 5-fold stratified cross-validation runs. The error bars for each column mark the 25th and 75th percentile. Our IMSP model achieved 0.854 for the infection F1-score and 0.867 for the PPI F1-score. Compared with other models, our model outperformed them in all the evaluation metrics except in PPI Precision. Specifically, in terms of infection F1-score, our model outperformed the second-best model Node2vec^45^ by 40.4%. In terms of PPI F1-score, our model also surpassed the second-best model Node2vec^45^ by 1.6%.

Table 1 shows the performance comparison measured in six common link prediction evaluation metrics. IMSP achieved an overall link prediction accuracy of 97.1% with a standard deviation (SD) of 0.005, which demonstrated a 7.7% gain compared with the second-best model. Our model also excelled in its weighted F1-score, achieving 0.971 with SD of 0.006, which exceeded the second-best model by 10.0%. The p values for these two metrics were all smaller than 0.01, which indicated significant improvement for our model. We also presented the performance on infection and PPI predictions (Figure 4). IMSP achieved an F1-score of 0.854 with a 0.090 SD for infection predictions, a 40.4% increase compared with the second-best model. The p value was smaller than 0.01, indicating a significant improvement in our model. For PPI predictions, our model achieved an F1-score of 0.867 with 0.034 SD, a 1.6% increment compared with the second-best model. The p value also demonstrated a significant improvement for IMSP under the 0.01 significance level. In conclusion, our model showed statistically significant improvements compared with all existing models in 11 of 12 evaluation metrics.

**Table 1:** Link prediction: Overall performance evaluation and comparison

Model	Accuracy	Weighted precision	Weighted recall	Weighted F1-score	AUC macro	AUC weighted
GF^49^	0.879 ± 0.008	0.852 ± 0.011	0.879 ± 0.008	0.863 ± 0.009	0.913 ± 0.007	0.944 ± 0.006
Deepwalk^43^	0.894 ± 0.008	0.870 ± 0.010	0.894 ± 0.008	0.879 ± 0.009	0.926 ± 0.010	0.952 ± 0.007
LINE^44^	0.742 ± 0.026	0.727 ± 0.030	0.742 ± 0.026	0.732 ± 0.029	0.874 ± 0.021	0.881 ± 0.023
Node2vec^45^	0.902 ± 0.007	0.868 ± 0.007	0.902 ± 0.007	0.883 ± 0.007	0.896 ± 0.011	0.932 ± 0.010
SDNE^46^	0.820 ± 0.015	0.791 ± 0.019	0.820 ± 0.015	0.799 ± 0.017	0.904 ± 0.012	0.930 ± 0.011
IMSP	0.971 ± 0.005	0.972 ± 0.006	0.971 ± 0.005	0.971 ± 0.006	0.997 ± 0.001	0.996 ± 0.001

AUC, area under the receiver-operating characteristic curve. This table presents six evaluation metrics regarding the link prediction performance of our model compared with five other baseline models. While evaluating performance, we followed 5-fold stratified cross-validation setting with shuffle enabled. This method preserved the percentage of samples for each class (i.e., type of edge) in each fold. We created a sampling strategy to ensure that the training subset in each cross-validation run can form a fully connected network. To ensure the balance of input data, we gathered negative (non-connected) edges in addition to positive (connected) edges that already existed in each fold. While sampling negative edges, we randomly selected some from known negative edges (i.e., true negatives), which consisted of spike-receptor interactions demonstrated as nonexistent. We randomly selected the remaining negative edges from other non-connected node pairs, which we assumed did not exist. These negative edges were then added to each fold to match the number of positive edges. We performed this 5-fold stratified cross-validation experiment for 30 runs. In each run, we would generate a new 5-fold split. We then performed two-sample heteroscedastic t tests for these six overall performance evaluation metrics to test the significance of IMSP improvement. Lastly, we reported the average with SD for each metric.

The high performance of IMSP might result from its ability to take full advantage of well-studied knowledge and data from previous biology research with protein-level variations. Thanks to the novel design of our virus-host interaction network, cross-organism information and multi-class linkage information can be well preserved. Another reason behind the performance improvement of IMSP is that it factors essential biological metadata for nodes into the learned representations of edges. This design substantially helped the classifier output a correct predicted class when formulating edge representations. However, around 10% of PPI predictions were unlikely predictions by our definition, i.e., PPIs between S-protein and non-receptor host proteins. To minimize unlikely predictions, we also utilized known negative edges (true negatives) in the protein layer to constitute part of the negative samples for training and testing. This finally reduced the unlikely PPI predictions to around 5%.

In conclusion, IMSP exhibited robust and stable performance in both top-level and detailed evaluation metrics, which was substantially improved compared with existing tools. When analyzing newly emerged viruses with limited available information, namely SARS-CoV-2, IMSP could provide reasonable and reliable predictions.

## Discussion

This study assembled 260 nodes and 1,995 known edges. Each node represented a virus/virus protein/host/host protein, and each edge represented a virus-host infection/PPI/protein-homolog similarity/organism-protein belonging. Based on this network, we predicted the potential host for viruses and undiscovered PPIs. Among all currently known seven human coronaviruses, SARS-CoV and MERS-CoV were relatively well studied in terms of interactions (i.e., infection and PPI). However, interactions of HCoV-OC43, HCoV-NL63, HCov-HKU1, HCoV-229E, and the newly emerged SARS-CoV-2 remained relatively less discovered. Our model predicted 939 PPIs and 24 infections that were likely to happen. These predictions need further experiments for validation.

Established discoveries about the viral interactions with host proteins were scarce for SARS-CoV-2. However, SARS-CoV-2 was highly suspected of suppressing the innate immune response and reducing the production of IFN. Thus, the findings by IMSP could help discover the protein-level mechanism of virus invasion and host response to provide clues toward developing therapeutic strategies for the treatment of this disease. Some of our prediction results have been revealed as meaningful. It should be noted that, during the review period, two of our prediction results were validated in wet-lab experiments by independent labs,^42^^,^^50^ which demonstrated that swine is susceptible to SARS-CoV-2 and that the M protein of SARS-CoV-2 inhibits IFN production by targeting RIG-I/MDA-5 signaling.

More broadly, IMSP could be applied to any other analysis of the virus-host interaction network predictions. IMSP would build the network based on the information of the PPIs, protein-homolog similarities, virus-host infection relations, and related protein function knowledge if available. Based on such a network, IMSP could predict high-possibility PPIs and infections. We hope to use this pipeline as a guideline for investigating various similar viruses and their mechanisms with hosts on both organism level and protein level.

### Limitations of the study

This section discusses the limitation of our work in terms of prediction validation, quality of data sources, model bias, and potential improvements. Concerning prediction validation, ideally wet-lab experiments should be conducted to validate our predictions, which require special facilities not commonly available. Thus, we were unable to validate our predictions through biological experiments. We collected protein sequences, infection relationships, and known PPIs from the best available data sources when carrying out this study. The quality, errors, and uncertainty of these data sources could affect the performance of our approach. This may harm the reliability of our predictions, and hence biologists should exercise extra caution when using our predictions to aid the design of experiments. Our approach may suffer from sampling bias, representation bias, and population bias.^51^ For example, we only included the proteins known to play crucial roles in viral entry and the IFN signaling pathway. It is possible that some related proteins were ignored, i.e., our model potentially carries sampling bias. Our model might also suffer from representation bias due to missing protein sequences, which could lead to non-uniform protein representation in different mammalian hosts in our network. Additionally, we could not include some mammals (e.g., rabbits and civets) because most of their protein sequences are either unavailable or of low quality in the National Center for Biotechnology Information (NCBI) database, which led to population bias. As more data become available, a more comprehensive network could be constructed by our IMSP model, which would substantially mitigate the model bias. Lastly, the model can also be improved by incorporating gene set enrichment and sequence motif analysis.

## Experimental procedures

### Resource availability

#### Lead contact

Further information and requests for code and data should be directed to and will be fulfilled by the lead contact, Hongfu Liu (hongfuliu@brandeis.edu).

#### Materials availability

This study did not generate any physical materials.

#### Data and code availability

All data and codes are available at Github repositories. IMSP model, its predictions, and performance evaluations can be found at https://github.com/hangyu98/IMSP; data and parsing code can be found at https://github.com/hangyu98/IMSP-Parser. Additional supplemental items are available from Mendeley Data at doi: 10.17632/3s2dr7y6s2.1.

### Virus-host interaction network data selection

The virus-host interaction network consists of two layers (an organism layer and a protein layer). The organism layer contains a set of viruses (including SARS-CoV-2, SARS-CoV, HCoV-229E, HCoV-HKU1, HCoV-OC43, HCoV-NL63, and MERS-CoV) and a set of hosts (including human, mouse, rat, dog, cat, camel, squirrel, cattle, chimpanzee, red junglefowl, rabbit, horse, monkey, rat, sheep, swine, and golden Syrian hamster). At the protein layer, we focus on proteins that are known to be involved in viral invasion or immune system response and suppression. The network contains 13 host protein-homolog groups obtained from NCBI: ACE2, DPP4, IRF3, IRF7, IRF9, MAVS, MDA5, NF-κB, PRKRA, TBK1, RIG-I, STAT1, and STAT2. The virus proteins include homologs of S-protein, M protein, nucleocapsid protein, nsp1, nsp15, ORF3b, ORF4a, ORF4b, ORF6, and papain-like protease (PLpro). There are four types of edges in the network: PPI, virus-host infection, organism-protein belonging, and similarity relation between protein homologs. PPI and infection relationships are gathered from academic publications.^9^, ^10^, ^11^, ^12^, ^13^, ^14^, ^15^, ^16^, ^17^, ^18^, ^19^, ^20^, ^21^, ^22^, ^23^, ^24^ Organism-protein belonging and protein-homolog similarity relation are innately connected. Detailed PPI data resources are presented in Table S5.

### Infection mechanism and spectrum prediction

Our IMSP model requests three inputs: pairwise similarity matrices (parsed from percentage of positives from NCBI BLASTp result) for protein homologs, a set of known PPIs and infections, and protein function data. Given these three inputs, the model constructs a heterogeneous two-layer virus-host interaction network. IMSP then performs graph representation learning and combines the structural embeddings with the content embeddings to form edge representations. Lastly, in the link prediction phase, IMSP trains a neural-network-based Multi-layer Perceptron (MLP) classifier on learned representations to perform multi-class classification task. Along with post-process procedures, our model outputs high-possibility undiscovered PPIs and infections. In the following, we elaborate on the two main steps of IMSP in terms of virus-host interaction network construction and representation learning, and virus-host interaction prediction. To show the design of our model, we present the pseudocode sample in Alg. 1 in supplemental information. The time complexity is O$\left(|V{|}^{2}\right)$ and the space complexity is O$\left(|V{|}^{2}\right)$. Please refer to Table 2 for notations.

**Table 2:** Notations

Notation	Description
$V_{i}$	node *i* in the network
*V*	the set of all nodes
$I_{i,j}$	edge between node *i* and node *j*
*I*	the set of all edges in the network
$R_{i}^{S}$	structure embedding vector for node *i*
$R_{i}^{C}$	content embedding vector for node *i*
$CE_{i,j}$	content embedding vector for edge $I_{i,j}$
$IE_{i,j}$	full edge embedding vector for edge $I_{i,j}$
$w_{i,j}$	edge weight for edge $I_{i,j}$
$ED_{i,j}$	Euclidean distance between node *i* and node *j*
$MD\left(R_{i}^{C},R_{j}^{C}\right)$	magnitude difference between vector $R_{i}^{C}$ and $R_{j}^{C}$
$TS-SS_{i,j}$	*TS*-*SS* similarity between vector $R_{i}^{C}$ and $R_{j}^{C}$

### Virus-host interaction network construction and representation learning

We utilized nodes to represent either organisms or proteins. Edges were used to represent PPI/infection/similarity/belonging relationships. To model the network, we constructed an undirected two-layer heterogeneous network using NetworkX.^52^ The network carried four groups of nodes: host, host protein, virus, and virus protein. We organized the virus group and the host group into the organism layer. Similarly, host protein groups and virus protein groups were put into the protein layer. By nature, the network held four types of edges: PPI (between virus protein groups and host protein groups), infection (between virus group and host group), protein-homolog similarity relation (between virus/host protein homologs in protein layer), and organism-protein belonging relation (between organism layer and protein layer). Protein-homolog similarity and organism-protein belonging relationships were innately connected. PPIs and infections were connected based on proven molecular level knowledge or infection data from existing research.^3^, ^4^, ^5^, ^6^, ^7^, ^8^, ^9^, ^10^, ^11^, ^12^, ^13^, ^14^, ^15^, ^16^, ^17^, ^18^, ^19^, ^20^, ^21^, ^22^, ^23^, ^24^^,^^53^, ^54^, ^55^ After building the network, the virus-host interaction network contained 260 nodes and 1,995 edges. Intuitively, if there is an interaction edge (infection or PPI) between two nodes $V_{i}$ and $V_{j}$, an edge with the same type (infection or PPI) is more likely to form between $V_{i}$ and another node with high biological similarity to $V_{j}$. We therefore designed a method that assigns a weight to each relationship in the network. A structure embedding model^45^ was then applied to factor in such information into the node representations, which is later used in predicting interactions between nodes. To be more specific, if a relationship connects two protein homologs, its weight is equal to the similarity between their full-length sequences. For other relationships, we calculated its weight as the similarity between the text content of the connected nodes. The text content of a node includes the name and molecular functions if a node represents a protein. The text content is processed by Text2vec, a Word2vec^56^-based model, to obtain the node content embedding denoted as $R_{i}^{C}$ for $V_{i}$. We then utilized the *TS-SS* similarity metric,^57^ a robust and reliable similarity measurement in the field of textual mining, to calculate $w_{i,j}$ as the *TS-SS* similarity between $R_{i}^{C}$ and $R_{j}^{C}$. The technical details are explained below:

(Equation 1) $$\begin{array}{c} TS-SS_{i,j}=\;\left|R_{i}^{C}\right|\cdot \left|R_{j}^{C}\right|\cdot \text{sin}(\theta^{'})\cdot \theta^{'}\cdot \pi \cdot {\left(ED\left(R_{i}^{C},R_{j}^{C}\right)\;+MD\left(R_{i}^{C},R_{j}^{C}\right)\right)}^{2}/720\text{,} \end{array}$$

where$MD\left(R_{i}^{C},R_{j}^{C}\right)$^57^ is defined as the magnitude difference between $R_{i}^{C}$ and $R_{j}^{C}$, which is calculated as

(Equation 2) $$MD\left(R_{i}^{C},R_{j}^{C}\right)=\left|\sqrt{\sum_{n=1}^{\text{dim}R_{i}^{C}}R_{i}^{C^{2}}}-\sqrt{\sum_{n=1}^{\text{dim}R_{j}^{C}}R_{j}^{C^{2}}}\right|\text{,}$$

and $\theta^{\prime }$ is defined as

(Equation 3) $$\theta^{\prime }={\text{cos}}^{-1}\left(\text{cos}\left(R_{i}^{C},R_{j}^{C}\right)\right)+10\text{.}$$

Note that $\theta^{\prime }$ is increased by 10° to overcome the problem of overlapping vectors. $w_{i,j}$ is then calculated as

(Equation 4) $$w_{i,j}=\sigma \left(TS-SS_{i,j}/\bar{TS-SS}\right)\text{,}$$

where *σ* is the sigmoid function, and $\bar{TS-SS}$ denotes the average of $TS-SS_{i,j}$, for all i,j, if i≠j and $V_{i},V_{j}\subset V$.

For graph representation learning, we captured the graph heterogeneity by adding the heterogeneous content information to its structural information. Specifically, we performed network structural embedding assuming the network is homogeneous. We then added the content embedding on top of structural embedding to model the heterogeneity.

First, for network structural embedding, we used a powerful network representation learning model, Node2vec,^45^ to learn the structural embedding for nodes. Node2vec is a state-of-the-art model for homogeneous network embedding. We took full advantage of the biased searching algorithm offered by Node2vec during our application. Precisely, the Node2vec model performed a biased fixed-length random walk for graph sampling, which takes edge weight into account. Let $c_{m}$ denote the *m*th node in walk with $c_{0}$ denoting the starting node of the current random walk. Nodes $c_{m}$ are generated by the following distribution:

(Equation 5) $$P\left(c_{m}=V_{i}\;|\;c_{m-1}=V_{j}\right)=\left\{ \begin{array}{ll} \pi_{V_{j},V_{i}}/Z & \text{if}\;I_{i,j}\subset I\\ 0 & \text{otherwise} \end{array}\right.\text{,}$$

where m⩾1, *Z* is the normalizing constant, and $\pi_{V_{j},V_{i}}$ is the unnormalized transition probability between $V_{j}$ and $V_{i}$, which is calculated as $\pi_{V_{j},V_{i}}=\alpha_{pq}\left(V_{t},V_{i}\right)\cdot w_{i,j}$. Note that the edge weight $w_{i,j}$ is taken into consideration. Assume we have just transitioned from $V_{t}$ to $V_{j}$ and are now evaluating the transition probability leaving $V_{j}$. Let $V_{i}$ represents the set of all neighbors of $V_{j}$. $\alpha_{pq}\left(V_{t},V_{i}\right)$, termed as search bias, is calculated as

(Equation 6) $$\alpha_{pq}(V_{t},V_{i})=\left\{\begin{array}{ccc} 1/p & if & d_{V_{t},V_{i}}=0\\ 1 & \mathrm{if} & d_{V_{t},V_{i}}=1\\ 1/q & \mathrm{if} & d_{V_{t},V_{i}}=2 \end{array}\right),$$

where $d_{V_{t},V_{i}}$ denotes the shortest path between $V_{t}$ and $V_{i}$.

In Equation 6, *p* (return hyperparameter) and *q* (in-out hyperparameter) are the two crucial hyperparameters of Node2vec. They can be adjusted to influence the probability of going back to $V_{i}$ after visiting $V_{j}$ and the probability of exploring the undiscovered components of the network. In this way, we were able to tune the hyperparameters of the structural embedding model, Node2vec, through a grid search algorithm to generate the structural embeddings.

Second, to generate edge content embeddings, i.e., $CE_{i,j}$ for all possible $I_{i,j}$, we combined the textualized node content (including name, group, layer, and function) of $V_{i}$ and $V_{j}$ with expected edge type such as PPI/infection/protein-homolog similarity/organism-protein belonging. We then input such text into Text2vec, a Word2vec^56^-based model, to generate edge content embeddings. The full edge representations that consider both structural and content information for edge $I_{i,j}$ are formulated as follows:

(Equation 7) $$\begin{array}{l} IE_{i,j}=\left[R_{i}^{S}\;,\;R_{j}^{S},\;CE_{i,j}\right],\\ IE_{j,i}=\left[R_{j}^{S}\;,\;R_{i}^{S},\;CE_{j,i}\right]. \end{array}$$

Note that by the nature of Text2vec, the order of input document does not affect its output, meaning that $CE_{i,j}$ is the same as $CE_{j,i}$. Upon finishing this step, we obtained all edge representations, $IE_{i,j}$, for all $V_{i}$ and $V_{j}$⊂V and i≠j.

### Virus-host interaction prediction

In the interaction prediction phase, we utilized a neural-network-based classification model, MLP classifier, provided by scikit-learn^58^ to perform multi-class classification. The classifier would classify edges into infection, PPI, no-interaction, organism-protein belongings, and similarity relations between protein homologs, using the learned edge representations. The predicted interactions (i.e., infection and PPI) would go through a post-processing step to eliminate unlikely interaction predictions. The processed result would be the output of IMSP.

Here we performed 5-fold stratified cross-validation. While splitting data into folds, we let each fold have roughly the same percentage of interactions in each interaction type. Besides, each fold has the same number of positive (i.e., known interactions) and negative (i.e., non-interaction) samples. It should be noted that the negatives consist of both validated non-interactions (e.g., the S-protein of SARS-CoV-2 is known not to bind well to the human ACE2 receptor) and other non-interactions that have yet to be validated experimentally. To mitigate the issue caused by sampling undiscovered true positive links as the negative training samples, we trained multiple independent MLP classifiers on different training sets, where the negative links were randomly sampled for each set. We then aggregated their edge classification results to pass to the post-processing step. We defined the following rules from both the computational and biological perspectives to remove unlikely predictions in the post-processing step. Computationally, since there exist two representations for $I_{i,j}$, i.e., $IE_{i,j}$ and $IE_{j,i}$, the prediction for $I_{i,j}$ is defined as a “strong” one if and only if both $IE_{i,j}$ and $IE_{j,i}$ are classified into the same interaction type (excluding the non-interaction type). Biologically, we assumed that the virus S-protein would only bind with its known target receptor.

## Supplemental information

Document S1. Figures S1–S5 and Tables S1, S3, and S5

Table S2. Edge table with edge types

Table S4. Predicted protein-protein interactions

Document S2. Article plus supplemental information

## Footnotes

Supplemental information can be found online at https://doi.org/10.1016/j.patter.2021.100242.
