# Supplementary material for: Network-based virus-host interaction prediction with application to SARS-CoV-2
Source: Patterns (N Y). 2021 Mar 29;2(5):100242. doi: 10.1016/j.patter.2021.100242 (PMC8006187; doi:10.1016/j.patter.2021.100242)
Supplement: Document S1. Figures S1–S5 and Tables S1, S3, and S5 [file mmc1.pdf]

Patterns, Volume *2*

Supplemental information

Network-based virus-host interaction prediction with application to SARS-CoV-2

Hangyu Du, Feng Chen, Hongfu Liu, and Pengyu Hong

This supplementary material contained visualized demonstrations of viral entry in Figure S1 and IFN pathway mechanisms in Figure S2. The full network with predictions made by the model was visualized in three figures: Figure S3 for viral entry, Figure S4 for IFN pathway and Figure S5 for host infection. The full nodes and edges in the network are presented in Table S1 and Table S2. The predicted interactions are presented in Table S3 and Table S4. The PPI data source are shown in Table S5. The IMSP algorithm is presented in Alg. 1.

Supplementary Note 1: Virus Entry - Receptor Binding of S Protein

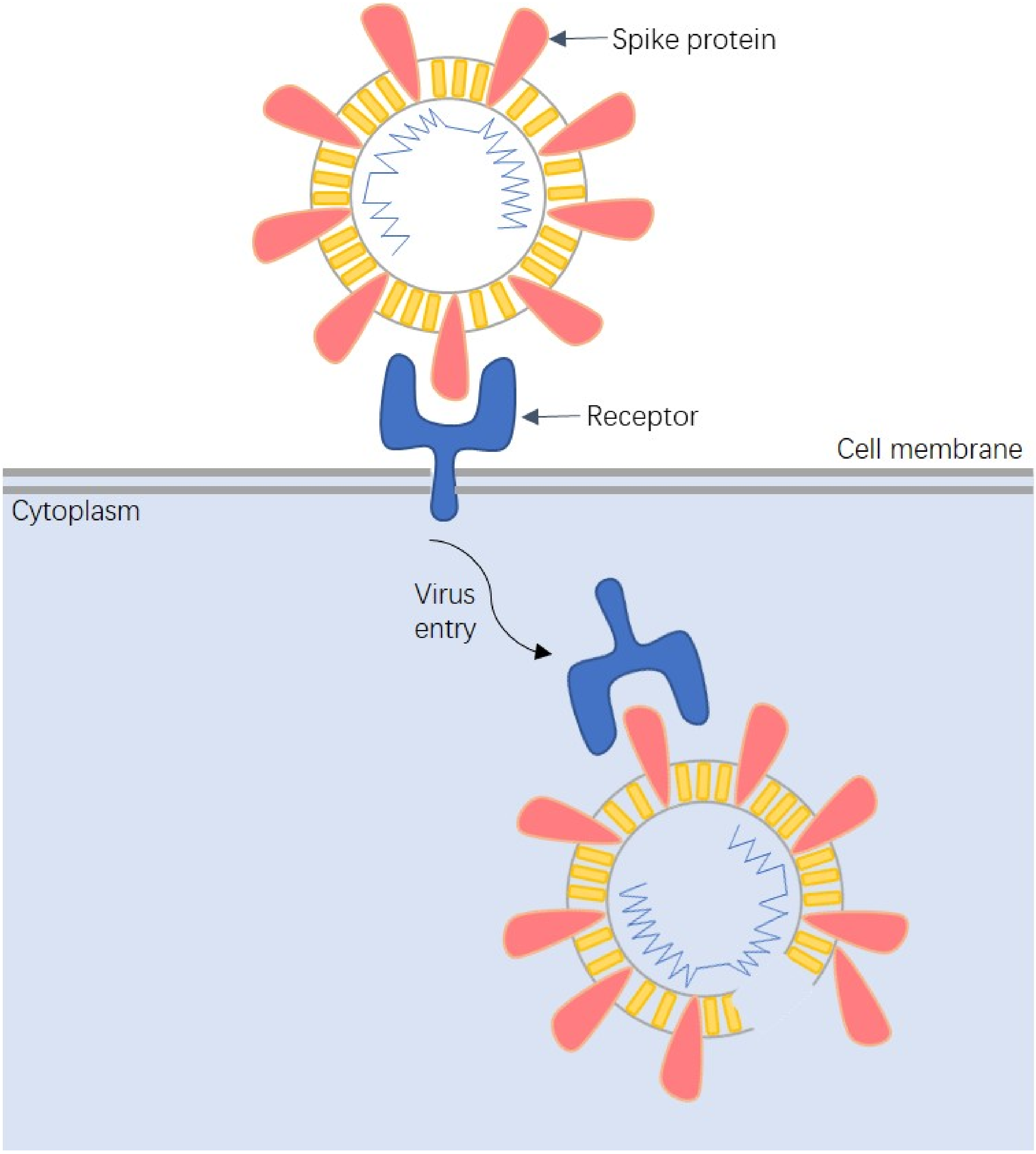

Fig. S1. The Process for Coronavirus Receptor Binding and Virus Entry. *The S protein in coronaviruses plays a crucial role in viral entry. It binds with host receptors and facilitates the fusion between the viral envelope and the host cell membrane.*

Supplementary Note 2: Immune Response - IFN Signaling Pathway

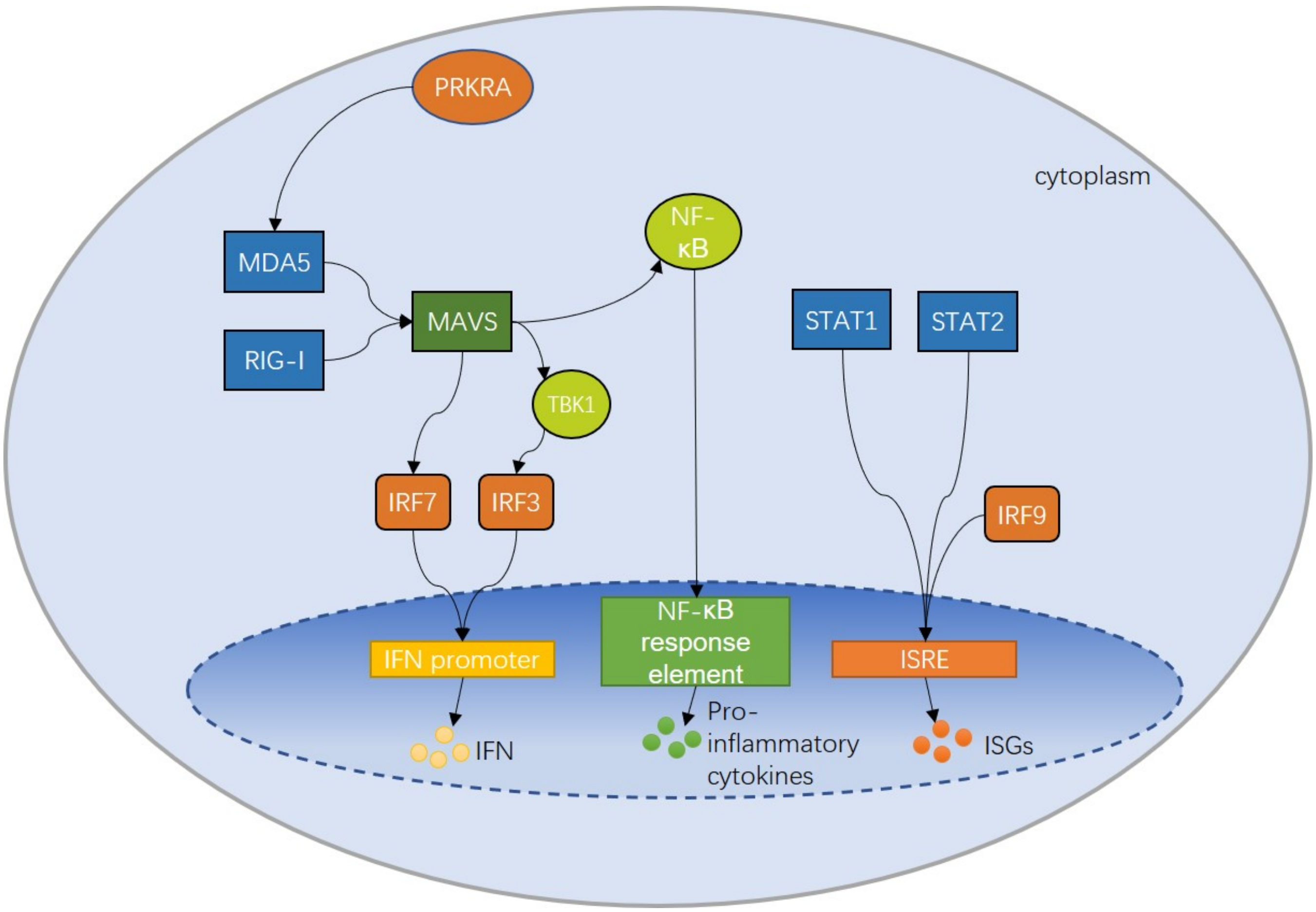

Fig. S2. Innate Immune Response to Coronaviruses' Viral Infection and IFN signaling Mechanism. *RIG-I and MDA5 detect the pattern of virus and trigger the production of Interferons (IFNs)*[S1] *and the activation of the NF-κB.*[S2] *The activated NF-κB induces the Pro-inflammatory cytokines,*[S3] *which play a central role in inflammatory diseases of infectious.*[S4] *STAT1 and STAT2 associate with IRF9 to induce the expression of interferon-stimulated genes (ISGs)*[S5] *and produce antiviral proteins.*[S6] *In this way, viral interactions with the host innate immune system to suppress immune responses become the critical determinant of the disease outcome and viral infection.*

Supplementary Note 3: Full Map of Binding Interactions between Coronaviruses' S Protein and Mammalian Hosts' Receptor

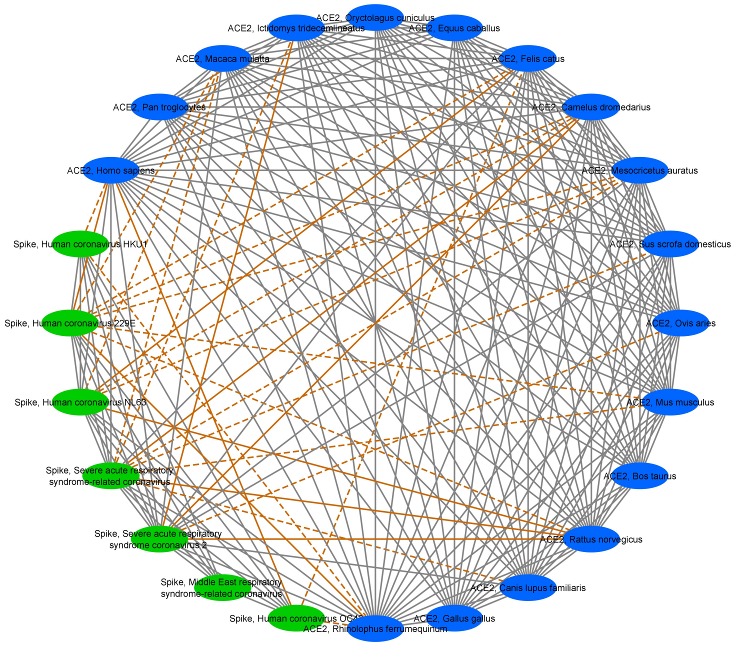

Fig. S3. Virus entry: binding relationships between the S-proteins of human coronaviruses and the ACE2 receptors of mammalian hosts. *This figure of the network is visualized by Cytoscape. Virus Protein nodes are represented in green, and Host Protein Layer nodes are represented in blue. The original interactions are represented in light grey lines, including the known receptor bindings between viruses spike and mammalian hosts ACE2. Predicted receptor bindings are represented in orange lines. As the infection relations have been checked for likelihood in the IMSP model, all predicted interactions are strong predicted interactions.*

Supplementary Note 4: Full Map of PPIs in IFN Signaling Pathway between Coronaviruses' Proteins and Mammalian Hosts' Proteins

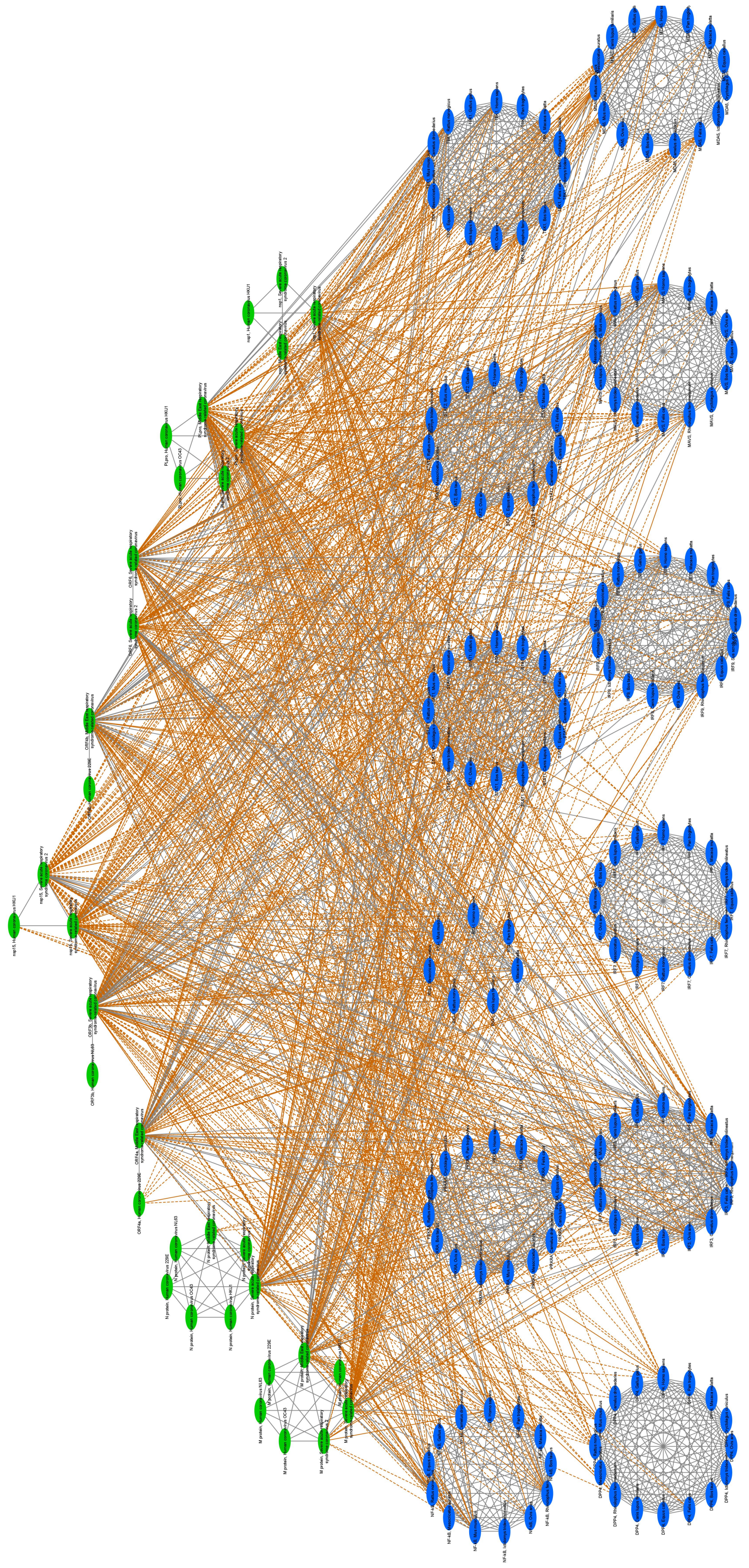

Fig. S4. IFN interactions: virus proteins interactions with IFN signaling pathway to suppress the IFN signaling. *Same representations for nodes and interactions as described in Figure 3. The predicted interactions are represented in orange lines: the solid lines stand for strong predictions, and dotted lines stand for weak predictions as defined in the IMSP model.*

Supplementary Note 5: Full Map of Infection Relationships between Coronaviruses and Mammalian Hosts

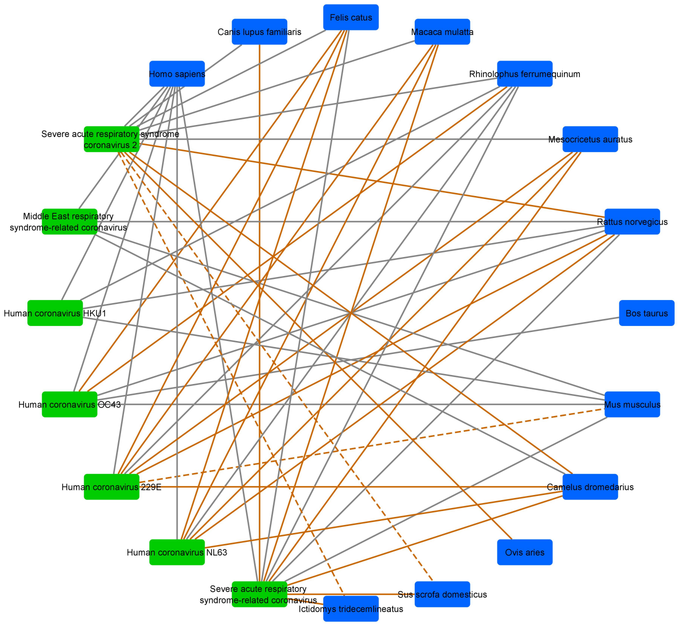

Fig. S5. Coronaviruses and mammalian hosts infection relationships *Virus Layer nodes are represented in green rhombi, and Host Layer nodes are represented in blue. The original infection interactions are represented in grey lines. Predicted infection relations are represented in orange lines: the solid lines stand for strong predictions and dotted lines stand for weak predictions as defined in the IMSP model.*

Supplementary Note 6: Network Node

Table S1. Node IDs and Node Full Names.

Node ID	Full name representation	Node ID	Full name representation
0	nsp15 Severe acute respiratory syndrome coronavirus 2	130	IRF7 Mus musculus
1	nsp15 Severe acute respiratory syndrome-related coronavirus	131	IRF7 Bos taurus
2	nsp15 Human coronavirus HKU1	132	IRF7 Canis lupus familiaris
3	STAT1 Homo sapiens	133	IRF7 Gallus gallus
4	STAT1 Pan troglodytes	134	MDA5 Homo sapiens
5	STAT1 Macaca mulatta	135	MDA5 Pan troglodytes
6	STAT1 Felis catus	136	MDA5 Macaca mulatta
7	STAT1 Camelus dromedarius	137	MDA5 Equus caballus
8	STAT1 Equus caballus	138	MDA5 Oryctolagus cuniculus
9	STAT1 Canis lupus familiaris	139	MDA5 Ictidomys tridecemlineatus
10	STAT1 Rhinolophus ferrumequinum	140	MDA5 Felis catus
11	STAT1 Bos taurus	141	MDA5 Camelus dromedarius
12	STAT1 Ovis aries	142	MDA5 Bos taurus
13	STAT1 Ictidomys tridecemlineatus	143	MDA5 Ovis aries
14	STAT1 Oryctolagus cuniculus	144	MDA5 Mus musculus
15	STAT1 Rattus norvegicus	145	MDA5 Rattus norvegicus
16	STAT1 Mus musculus	146	MDA5 Mesocricetus auratus
17	STAT1 Mesocricetus auratus	147	MDA5 Canis lupus familiaris
18	STAT1 Gallus gallus	148	MDA5 Gallus gallus
19	IRF9 Homo sapiens	149	PRKRA Homo sapiens
20	IRF9 Macaca mulatta	150	PRKRA Macaca mulatta
21	IRF9 Pan troglodytes	151	PRKRA Felis catus
22	IRF9 Felis catus	152	PRKRA Equus caballus
23	IRF9 Camelus dromedarius	153	PRKRA Canis lupus familiaris
24	IRF9 Sus scrofa domesticus	154	PRKRA Camelus dromedarius
25	IRF9 Equus caballus	155	PRKRA Mesocricetus auratus
26	IRF9 Rhinolophus ferrumequinum	156	PRKRA Mus musculus
27	IRF9 Ovis aries	157	PRKRA Ictidomys tridecemlineatus
28	IRF9 Canis lupus familiaris	158	PRKRA Ovis aries
29	IRF9 Bos taurus	159	PRKRA Bos taurus
30	IRF9 Ictidomys tridecemlineatus	160	PRKRA Rattus norvegicus
31	IRF9 Oryctolagus cuniculus	161	PRKRA Rhinolophus ferrumequinum
32	IRF9 Mus musculus	162	PRKRA Oryctolagus cuniculus
33	IRF9 Mesocricetus auratus	163	PRKRA Pan troglodytes
34	IRF9 Rattus norvegicus	164	ORF3b Severe acute respiratory syndrome-related coronavirus
35	IRF9 Gallus gallus	165	ORF3b Human coronavirus NL63
36	RIG-I Homo sapiens	166	DPP4 Homo sapiens
37	RIG-I Pan troglodytes	167	DPP4 Pan troglodytes
38	RIG-I Macaca mulatta	168	DPP4 Macaca mulatta
39	RIG-I Canis lupus familiaris	169	DPP4 Oryctolagus cuniculus
40	RIG-I Rattus norvegicus	170	DPP4 Ovis aries
41	RIG-I Mesocricetus auratus	171	DPP4 Ictidomys tridecemlineatus
42	RIG-I Mus musculus	172	DPP4 Bos taurus
43	ORF4b Middle East respiratory syndrome-related coronavirus	173	DPP4 Felis catus
44	ORF4b Human coronavirus 229E	174	DPP4 Equus caballus
45	nsp1 Severe acute respiratory syndrome coronavirus 2	175	DPP4 Canis lupus familiaris
46	nsp1 Severe acute respiratory syndrome-related coronavirus	176	DPP4 Rhinolophus ferrumequinum
47	nsp1 Middle East respiratory syndrome-related coronavirus	177	DPP4 Mesocricetus auratus
48	nsp1 Human coronavirus HKU1	178	DPP4 Rattus norvegicus
49	Spike Human coronavirus OC43	179	DPP4 Mus musculus
50	Spike Human coronavirus HKU1	180	DPP4 Camelus dromedarius
51	Spike Middle East respiratory syndrome-related coronavirus	181	DPP4 Gallus gallus
52	Spike Severe acute respiratory syndrome coronavirus 2	182	ORF6 Severe acute respiratory syndrome-related coronavirus
53	Spike Severe acute respiratory syndrome-related coronavirus	183	ORF6 Severe acute respiratory syndrome coronavirus 2
54	Spike Human coronavirus NL63	184	STAT2 Homo sapiens
55	Spike Human coronavirus 229E	185	STAT2 Pan troglodytes
56	IRF3 Homo sapiens	186	STAT2 Macaca mulatta
57	IRF3 Pan troglodytes	187	STAT2 Felis catus
58	IRF3 Macaca mulatta	188	STAT2 Canis lupus familiaris
59	IRF3 Ictidomys tridecemlineatus	189	STAT2 Camelus dromedarius
60	IRF3 Rhinolophus ferrumequinum	190	STAT2 Rhinolophus ferrumequinum
61	IRF3 Felis catus	191	STAT2 Equus caballus
62	IRF3 Camelus dromedarius	192	STAT2 Ovis aries
63	IRF3 Ovis aries	193	STAT2 Bos taurus
64	IRF3 Bos taurus	194	STAT2 Mesocricetus auratus
65	IRF3 Equus caballus	195	STAT2 Rattus norvegicus
66	IRF3 Oryctolagus cuniculus	196	STAT2 Ictidomys tridecemlineatus
67	IRF3 Rattus norvegicus	197	STAT2 Mus musculus
68	IRF3 Mesocricetus auratus	198	STAT2 Gallus gallus
69	IRF3 Mus musculus	199	PLpro Middle East respiratory syndrome-related coronavirus
70	IRF3 Canis lupus familiaris	200	PLpro Severe acute respiratory syndrome-related coronavirus
71	IRF3 Gallus gallus	201	PLpro Severe acute respiratory syndrome coronavirus 2
72	N protein Middle East respiratory syndrome-related coronavirus	202	PLpro Human coronavirus OC43
73	N protein Severe acute respiratory syndrome coronavirus 2	203	PLpro Human coronavirus HKU1
74	N protein Severe acute respiratory syndrome-related coronavirus	204	Homo sapiens
75	N protein Human coronavirus HKU1	205	Mus musculus
76	N protein Human coronavirus OC43	206	Rattus norvegicus

Table S1. Node IDs and Node Full Names.

Node ID	Full name representation	Node ID	Full name representation
77	N protein Human coronavirus 229E	207	Canis lupus familiaris
78	N protein Human coronavirus NL63	208	Camelus dromedarius
79	Human coronavirus OC43	209	Felis catus
80	Human coronavirus HKU1	210	Ictidomys tridecemlineatus
81	Middle East respiratory syndrome-related coronavirus	211	Bos taurus
82	Severe acute respiratory syndrome coronavirus 2	212	Pan troglodytes
83	Severe acute respiratory syndrome-related coronavirus	213	Gallus gallus
84	Human coronavirus NL63	214	Oryctolagus cuniculus
85	Human coronavirus 229E	215	Equus caballus
86	ACE2 Homo sapiens	216	Macaca mulatta
87	ACE2 Pan troglodytes	217	Ovis aries
88	ACE2 Macaca mulatta	218	Sus scrofa domesticus
89	ACE2 Ictidomys tridecemlineatus	219	Rhinolophus ferrumequinum
90	ACE2 Oryctolagus cuniculus	220	Mesocricetus auratus
91	ACE2 Equus caballus	221	M protein Middle East respiratory syndrome-related coronavirus
92	ACE2 Felis catus	222	M protein Human coronavirus HKU1
93	ACE2 Camelus dromedarius	223	M protein Severe acute respiratory syndrome-related coronavirus
94	ACE2 Mesocricetus auratus	224	M protein Severe acute respiratory syndrome coronavirus 2
95	ACE2 Sus scrofa domesticus	225	M protein Human coronavirus OC43
96	ACE2 Ovis aries	226	M protein Human coronavirus NL63
97	ACE2 Mus musculus	227	M protein Human coronavirus 229E
98	ACE2 Bos taurus	228	TBK1 Homo sapiens
99	ACE2 Rattus norvegicus	229	TBK1 Pan troglodytes
100	ACE2 Rhinolophus ferrumequinum	230	TBK1 Macaca mulatta
101	ACE2 Canis lupus familiaris	231	TBK1 Oryctolagus cuniculus
102	ACE2 Gallus gallus	232	TBK1 Ictidomys tridecemlineatus
103	ORF4a Middle East respiratory syndrome-related coronavirus	233	TBK1 Felis catus
104	ORF4a Human coronavirus 229E	234	TBK1 Bos taurus
105	NF-κB Homo sapiens	235	TBK1 Rhinolophus ferrumequinum
106	NF-κB Pan troglodytes	236	TBK1 Ovis aries
107	NF-κB Macaca mulatta	237	TBK1 Canis lupus familiaris
108	NF-κB Bos taurus	238	TBK1 Equus caballus
109	NF-κB Rhinolophus ferrumequinum	239	TBK1 Mesocricetus auratus
110	NF-κB Ovis aries	240	TBK1 Mus musculus
111	NF-κB Ictidomys tridecemlineatus	241	TBK1 Camelus dromedarius
112	NF-κB Mus musculus	242	TBK1 Rattus norvegicus
113	NF-κB Mesocricetus auratus	243	TBK1 Gallus gallus
114	NF-κB Rattus norvegicus	244	MAVS Homo sapiens
115	NF-κB Equus caballus	245	MAVS Pan troglodytes
116	NF-κB Gallus gallus	246	MAVS Macaca mulatta
117	NF-κB Camelus dromedarius	247	MAVS Ovis aries
118	IRF7 Homo sapiens	248	MAVS Equus caballus
119	IRF7 Pan troglodytes	249	MAVS Bos taurus
120	IRF7 Macaca mulatta	250	MAVS Oryctolagus cuniculus
121	IRF7 Ictidomys tridecemlineatus	251	MAVS Rhinolophus ferrumequinum
122	IRF7 Equus caballus	252	MAVS Felis catus
123	IRF7 Rhinolophus ferrumequinum	253	MAVS Camelus dromedarius
124	IRF7 Felis catus	254	MAVS Ictidomys tridecemlineatus
125	IRF7 Camelus dromedarius	255	MAVS Canis lupus familiaris
126	IRF7 Rattus norvegicus	256	MAVS Mesocricetus auratus
127	IRF7 Oryctolagus cuniculus	257	MAVS Mus musculus
128	IRF7 Mesocricetus auratus	258	MAVS Rattus norvegicus
129	IRF7 Ovis aries	259	MAVS Gallus gallus

Supplementary Note 7: Edge Table

Table S2. Edge Table with Edge Types

Supplementary Note 8: IMSP Predicted Infections

Certainty is the probability score for the predictions made by IMSP, ranging from 0%-100%. Confidence is the computational rule set in IMSP. Strong confidence represents that for a predicted interaction $E_{i,j}$, its two edge representations $EE_{i,j}$ and $EE_{j,i}$ are all classified into the same class other than the no-interaction class. For weak confidence interactions, only one representation is classified into the class other than the no-interaction class. Likelihood is the biological rule to validate the predictions. Based on pre-defined filters, the unlikely interactions are predictions that have conflicts with those filters.

Table S3. Predicted Infections Table

Source Name	Target Name	Certainty	Confidence	Likelihood
virus Human coronavirus OC43	host Rhinolophus ferrumequinum	96.51%	strong	likely
virus Severe acute respiratory syndrome-related coronavirus	host Macaca mulatta	96.45%	strong	likely
virus Human coronavirus 229E	host Rattus norvegicus	94.14%	strong	likely
virus Severe acute respiratory syndrome-related coronavirus	host Mesocricetus auratus	94.10%	strong	likely
virus Severe acute respiratory syndrome coronavirus 2	host Rattus norvegicus	91.86%	strong	likely
virus Human coronavirus NL63	host Rattus norvegicus	91.15%	strong	likely
virus Severe acute respiratory syndrome-related coronavirus	host Camelus dromedarius	90.80%	strong	likely
virus Human coronavirus 229E	host Camelus dromedarius	87.21%	strong	likely
virus Human coronavirus 229E	host Mesocricetus auratus	85.17%	strong	likely
virus Human coronavirus OC43	host Felis catus	84.30%	strong	likely
virus Severe acute respiratory syndrome-related coronavirus	host Canis lupus familiaris	83.64%	strong	likely
virus Human coronavirus NL63	host Camelus dromedarius	82.39%	strong	likely
virus Human coronavirus 229E	host Macaca mulatta	79.19%	strong	likely
virus Human coronavirus NL63	host Mesocricetus auratus	78.32%	strong	likely
virus Severe acute respiratory syndrome-related coronavirus	host Ictidomys tridecemlineatus	74.45%	strong	likely
virus Human coronavirus 229E	host Felis catus	73.59%	strong	likely
virus Severe acute respiratory syndrome coronavirus 2	host Camelus dromedarius	68.71%	strong	likely
virus Human coronavirus NL63	host Macaca mulatta	68.07%	strong	likely
virus Severe acute respiratory syndrome coronavirus 2	host Ovis aries	65.65%	strong	likely
virus Human coronavirus NL63	host Felis catus	65.62%	strong	likely
virus Severe acute respiratory syndrome-related coronavirus	host Sus scrofa domesticus	62.09%	strong	likely
virus Human coronavirus 229E	host Mus musculus	86.12%	weak	likely
virus Severe acute respiratory syndrome coronavirus 2	host Ictidomys tridecemlineatus	66.26%	weak	likely
virus Severe acute respiratory syndrome coronavirus 2	host Sus scrofa domesticus	59.56%	weak	likely
virus Human coronavirus HKU1	host Mesocricetus auratus	94.84%	strong	unlikely
virus Human coronavirus HKU1	host Camelus dromedarius	94.42%	strong	unlikely
virus Middle East respiratory syndrome-related coronavirus	host Rhinolophus ferrumequinum	93.21%	strong	unlikely
virus Human coronavirus OC43	host Camelus dromedarius	92.94%	strong	unlikely
virus Human coronavirus HKU1	host Macaca mulatta	91.85%	strong	unlikely
virus Human coronavirus OC43	host Mesocricetus auratus	90.58%	strong	unlikely
virus Human coronavirus HKU1	host Felis catus	89.32%	strong	unlikely
virus Middle East respiratory syndrome-related coronavirus	host Macaca mulatta	88.46%	strong	unlikely
virus Severe acute respiratory syndrome-related coronavirus	host Ovis aries	87.95%	strong	unlikely
virus Middle East respiratory syndrome-related coronavirus	host Mesocricetus auratus	84.07%	strong	unlikely
virus Human coronavirus OC43	host Macaca mulatta	83.75%	strong	unlikely
virus Severe acute respiratory syndrome-related coronavirus	host Bos taurus	80.89%	strong	unlikely
virus Human coronavirus HKU1	host Bos taurus	80.83%	strong	unlikely
virus Middle East respiratory syndrome-related coronavirus	host Ovis aries	79.52%	strong	unlikely
virus Human coronavirus HKU1	host Ovis aries	76.10%	strong	unlikely
virus Middle East respiratory syndrome-related coronavirus	host Felis catus	74.00%	strong	unlikely
virus Middle East respiratory syndrome-related coronavirus	host Ictidomys tridecemlineatus	69.40%	strong	unlikely
virus Human coronavirus OC43	host Ovis aries	67.82%	strong	unlikely
virus Human coronavirus HKU1	host Canis lupus familiaris	63.01%	strong	unlikely
virus Human coronavirus OC43	host Sus scrofa domesticus	51.93%	strong	unlikely
virus Human coronavirus 229E	host Ovis aries	47.25%	strong	unlikely
virus Middle East respiratory syndrome-related coronavirus	host Bos taurus	92.83%	weak	unlikely
virus Human coronavirus 229E	host Bos taurus	83.89%	weak	unlikely
virus Human coronavirus NL63	host Mus musculus	82.16%	weak	unlikely
virus Human coronavirus OC43	host Ictidomys tridecemlineatus	81.23%	weak	unlikely
virus Severe acute respiratory syndrome-related coronavirus	host Equus caballus	80.57%	weak	unlikely
virus Middle East respiratory syndrome-related coronavirus	host Canis lupus familiaris	77.79%	weak	unlikely
virus Middle East respiratory syndrome-related coronavirus	host Pan troglodytes	77.02%	weak	unlikely
virus Middle East respiratory syndrome-related coronavirus	host Sus scrofa domesticus	76.57%	weak	unlikely
virus Human coronavirus NL63	host Ovis aries	75.21%	weak	unlikely
virus Human coronavirus NL63	host Bos taurus	74.95%	weak	unlikely
virus Human coronavirus HKU1	host Ictidomys tridecemlineatus	74.01%	weak	unlikely
virus Severe acute respiratory syndrome-related coronavirus	host Pan troglodytes	73.98%	weak	unlikely
virus Human coronavirus HKU1	host Equus caballus	73.25%	weak	unlikely
virus Severe acute respiratory syndrome coronavirus 2	host Bos taurus	69.41%	weak	unlikely
virus Human coronavirus HKU1	host Pan troglodytes	67.38%	weak	unlikely
virus Human coronavirus HKU1	host Sus scrofa domesticus	65.95%	weak	unlikely
virus Human coronavirus OC43	host Gallus gallus	65.57%	weak	unlikely
virus Severe acute respiratory syndrome coronavirus 2	host Equus caballus	63.82%	weak	unlikely
virus Human coronavirus 229E	host Sus scrofa domesticus	62.90%	weak	unlikely
virus Severe acute respiratory syndrome-related coronavirus	host Gallus gallus	62.54%	weak	unlikely
virus Human coronavirus OC43	host Pan troglodytes	61.95%	weak	unlikely
virus Human coronavirus 229E	host Equus caballus	61.42%	weak	unlikely
virus Severe acute respiratory syndrome coronavirus 2	host Pan troglodytes	60.91%	weak	unlikely
virus Human coronavirus 229E	host Canis lupus familiaris	59.83%	weak	unlikely
virus Human coronavirus OC43	host Canis lupus familiaris	58.86%	weak	unlikely
virus Human coronavirus NL63	host Equus caballus	57.11%	weak	unlikely
virus Human coronavirus HKU1	host Gallus gallus	56.75%	weak	unlikely
virus Human coronavirus NL63	host Canis lupus familiaris	56.03%	weak	unlikely

Table S3. Predicted Infections Table

Source Name	Target Name	Certainty	Confidence	Likelihood
virus Human coronavirus 229E	host Ictidomys tridecemlineatus	54.27%	weak	unlikely
virus Human coronavirus OC43	host Equus caballus	53.57%	weak	unlikely
virus Human coronavirus NL63	host Ictidomys tridecemlineatus	52.83%	weak	unlikely
virus Middle East respiratory syndrome-related coronavirus	host Equus caballus	52.31%	weak	unlikely
virus Human coronavirus NL63	host Sus scrofa domesticus	50.30%	weak	unlikely
virus Severe acute respiratory syndrome coronavirus 2	host Gallus gallus	49.51%	weak	unlikely

Supplementary Note 9: IMSP Predicted PPIs

Table S4. Predicted Protein-Protein Interactions Table

Supplementary Note 10: PPIs Data Source

Table S5. PPIs Data Source Table

Virus Protein	Host Protein	Source
SARS-CoV-2 nsp15	IRF3	Yuen CK, Lam JY, Wong WM, et al. SARS-CoV-2 nsp13, nsp14, nsp15 and orf6 function as potent interferon antagonists. Emerg Microbes Infect. 2020;9(1):1418-1428. doi:10.1080/22221751.2020.1780953
SARS-CoV-2 nsp15	RIG-I	Yuen CK, Lam JY, Wong WM, et al. SARS-CoV-2 nsp13, nsp14, nsp15 and orf6 function as potent interferon antagonists. Emerg Microbes Infect. 2020;9(1):1418-1428. doi:10.1080/22221751.2020.1780953
SARS-CoV nsp15	IRF3	Yuen CK, Lam JY, Wong WM, et al. SARS-CoV-2 nsp13, nsp14, nsp15 and orf6 function as potent interferon antagonists. Emerg Microbes Infect. 2020;9(1):1418-1428. doi:10.1080/22221751.2020.1780953
SARS-CoV-2 ORF6	IRF3	Yuen CK, Lam JY, Wong WM, et al. SARS-CoV-2 nsp13, nsp14, nsp15 and orf6 function as potent interferon antagonists. Emerg Microbes Infect. 2020;9(1):1418-1428. doi:10.1080/22221751.2020.1780953
SARS-CoV-2 ORF6	RIG-I	Yuen CK, Lam JY, Wong WM, et al. SARS-CoV-2 nsp13, nsp14, nsp15 and orf6 function as potent interferon antagonists. Emerg Microbes Infect. 2020;9(1):1418-1428. doi:10.1080/22221751.2020.1780953
SARS-CoV nsp15	MAVS	Lei Y, Moore CB, Liesman RM, et al. MAVS-mediated apoptosis and its inhibition by viral proteins. PLoS One. 2009;4(5):e5466. doi:10.1371/journal.pone.0005466
HCoV-HKU1 nsp15	MAVS	Lei Y, Moore CB, Liesman RM, et al. MAVS-mediated apoptosis and its inhibition by viral proteins. PLoS One. 2009;4(5):e5466. doi:10.1371/journal.pone.0005466
SARS-CoV PLpro	TBK1	Siu KL, Kok KH, Ng MH, et al. Severe acute respiratory syndrome coronavirus M protein inhibits type I interferon production by impeding the formation of TRAF3.TANK.TBK1/IKKepsilon complex. J Biol Chem. 2009;284(24):16202-16209. doi:10.1074/jbc.M109.008227
MERS-CoV ORF4b	TBK1	Yang, Y., Ye, F., Zhu, N. et al. Middle East respiratory syndrome coronavirus ORF4b protein inhibits type I interferon production through both cytoplasmic and nuclear targets. Sci Rep 5, 17554 (2015). https://doi.org/10.1038/srep17554
MERS-CoV M protein	TBK1	Lui, P. L., Wong, L. Y. R., Fung, C. L., Siu, K. L., Yeung, M. L., Yuen, K. S., et al. (2016). Middle East respiratory syndrome coronavirus M protein suppresses type I interferon expression through the inhibition of TBK1-dependent phosphorylation of IRF3. Emerg. Microbes Infect. 5:e39. doi: 10.1038/emi.2016.33
MERS-CoV M protein	IRF3	Lui, P. L., Wong, L. Y. R., Fung, C. L., Siu, K. L., Yeung, M. L., Yuen, K. S., et al. (2016). Middle East respiratory syndrome coronavirus M protein suppresses type I interferon expression through the inhibition of TBK1-dependent phosphorylation of IRF3. Emerg. Microbes Infect. 5:e39. doi: 10.1038/emi.2016.33
MERS-CoV PLpro	TBK1	Sun L, Xing Y, Chen X, et al. Coronavirus papain-like proteases negatively regulate antiviral innate immune response through disruption of STING-mediated signaling. PLoS One. 2012;7(2):e30802. doi:10.1371/journal.pone.0030802
MERS-CoV S protein	DPP4	Zhao, J. et al. Rapid generation of a mouse model for Middle East respiratory syndrome. Proc. Natl Acad. Sci. USA 111, 4970–4975 (2014).
MERS-CoV ORF4a	NF-κB	Yang, Y. et al. The structural and accessory proteins M, ORF 4a, ORF 4b, and ORF 5 of Middle East respiratory syndrome coronavirus (MERS-CoV) are potent interferon antagonists. Protein Cell 4, 951–961 (2013).
MERS-CoV ORF4a	IRF3	Yang, Y. et al. The structural and accessory proteins M, ORF 4a, ORF 4b, and ORF 5 of Middle East respiratory syndrome coronavirus (MERS-CoV) are potent interferon antagonists. Protein Cell 4, 951–961 (2013).
MERS-CoV ORF4b	NF-κB	Yang, Y. et al. The structural and accessory proteins M, ORF 4a, ORF 4b, and ORF 5 of Middle East respiratory syndrome coronavirus (MERS-CoV) are potent interferon antagonists. Protein Cell 4, 951–961 (2013).
MERS-CoV ORF4b	IRF3	Yang, Y. et al. The structural and accessory proteins M, ORF 4a, ORF 4b, and ORF 5 of Middle East respiratory syndrome coronavirus (MERS-CoV) are potent interferon antagonists. Protein Cell 4, 951–961 (2013).
MERS-CoV PLpro	NF-κB	Bailey-Elkin, B. A. et al. Crystal structure of the Middle East respiratory syndrome coronavirus (MERS-CoV) papain-like protease bound to ubiquitin facilitates targeted disruption of deubiquitinating activity to demonstrate its role in innate immune suppression. J. Biol. Chem. 289, 34667–34682 (2014).
MERS-CoV PLpro	IRF3	Bailey-Elkin, B. A. et al. Crystal structure of the Middle East respiratory syndrome coronavirus (MERS-CoV) papain-like protease bound to ubiquitin facilitates targeted disruption of deubiquitinating activity to demonstrate its role in innate immune suppression. J. Biol. Chem. 289, 34667–34682 (2014).
SARS-CoV PLpro	NK-κB	Frieman, M., Ratia, K., Johnston, R. E., Mesecar, A. D. & Baric, R. S. Severe acute respiratory syndrome coronavirus papain-like protease ubiquitin-like domain and catalytic domain regulate antagonism of IRF3 and NF-$\varkappa$B signaling. J. Virol. 83, 6689–6705 (2009).
SARS-CoV PLpro	IRF3	Frieman, M., Ratia, K., Johnston, R. E., Mesecar, A. D. & Baric, R. S. Severe acute respiratory syndrome coronavirus papain-like protease ubiquitin-like domain and catalytic domain regulate antagonism of IRF3 and NF-$\varkappa$B signaling. J. Virol. 83, 6689–6705 (2009).
SARS-CoV ORF6	IRF3	Kopecky-Bromberg, S. A., Martinez-Sobrido, L., Frieman, M., Baric, R. A. & Palese, P. Severe acute respiratory syndrome coronavirus open reading frame (ORF) 3b, ORF 6, and nucleocapsid proteins function as interferon antagonists. J. Virol. 81, 548–557 (2007).
SARS-CoV ORF6	IRF9	Emmie de Wit, Neeltje van Doremalen, Darryl Falzarano, and Vincent J Munster. Sars and mers: recentinsights into emerging coronaviruses.Nature reviews. Microbiology, 14(8):523—534, August 2016
SARS-CoV ORF6	STAT1	Kopecky-Bromberg, S. A., Martinez-Sobrido, L., Frieman, M., Baric, R. A. & Palese, P. Severe acute respiratory syndrome coronavirus open reading frame (ORF) 3b, ORF 6, and nucleocapsid proteins function as interferon antagonists. J. Virol. 81, 548–557 (2007).
SARS-CoV ORF6	STAT2	Emmie de Wit, Neeltje van Doremalen, Darryl Falzarano, and Vincent J Munster. Sars and mers: recentinsights into emerging coronaviruses.Nature reviews. Microbiology, 14(8):523—534, August 2016
SARS-CoV ORF3b	STAT2	Emmie de Wit, Neeltje van Doremalen, Darryl Falzarano, and Vincent J Munster. Sars and mers: recentinsights into emerging coronaviruses.Nature reviews. Microbiology, 14(8):523—534, August 2016
SARS-CoV ORF3b	STAT1	Kopecky-Bromberg, S. A., Martinez-Sobrido, L., Frieman, M., Baric, R. A. & Palese, P. Severe acute respiratory syndrome coronavirus open reading frame (ORF) 3b, ORF 6, and nucleocapsid proteins function as interferon antagonists. J. Virol. 81, 548–557 (2007).
SARS-CoV ORF3b	IRF3	Kopecky-Bromberg, S. A., Martinez-Sobrido, L., Frieman, M., Baric, R. A. & Palese, P. Severe acute respiratory syndrome coronavirus open reading frame (ORF) 3b, ORF 6, and nucleocapsid proteins function as interferon antagonists. J. Virol. 81, 548–557 (2007).
SARS-CoV ORF3b	IRF9	Emmie de Wit, Neeltje van Doremalen, Darryl Falzarano, and Vincent J Munster. Sars and mers: recentinsights into emerging coronaviruses.Nature reviews. Microbiology, 14(8):523—534, August 2016
HCoV-NL63 S proten	ACE2	Hofmann H, Pyrc K, van der Hoek L, Geier M, Berkhout B, Pöhlmann S. Human coronavirus NL63 employs the severe acute respiratory syndrome coronavirus receptor for cellular entry. Proc Natl Acad Sci U S A. 2005;102(22):7988-7993. doi:10.1073/pnas.0409465102

Table S5. PPIs Data Source Table

Virus Protein	Host Protein	Source
MERS-CoV M protein	TBK1	Pak-Yin Lui, Lok-Yin Roy Wong, Cheuk-Lai Fung, Kam-Leung Siu, Man-Lung Yeung, Kit-San Yuen, Chi-Ping Chan, Patrick Chiu-Yat Woo, Kwok-Yung Yuen, and Dong-Yan Jin. Mid-dle east respiratory syndrome coronavirus m protein suppresses type i interferon expressionthrough the inhibition of tbk1-dependent phosphorylation of irf3.Emerging microbes & in-fections, 5(1):1–9, 2016
MERS-CoV M protein	STAT1	Emmie de Wit, Neeltje van Doremalen, Darryl Falzarano, and Vincent J Munster. Sars and mers: recentinsights into emerging coronaviruses.Nature reviews. Microbiology, 14(8):523—534, August 2016
MERS-CoV M protein	STAT2	Emmie de Wit, Neeltje van Doremalen, Darryl Falzarano, and Vincent J Munster. Sars and mers: recentinsights into emerging coronaviruses.Nature reviews. Microbiology, 14(8):523—534, August 2016
MERS-CoV M protein	IRF9	Emmie de Wit, Neeltje van Doremalen, Darryl Falzarano, and Vincent J Munster. Sars and mers: recentinsights into emerging coronaviruses.Nature reviews. Microbiology, 14(8):523—534, August 2016
MERS-CoV ORF4a	MDA5	Niemeyer, Daniela et al. "Middle East respiratory syndrome coronavirus accessory protein 4a is a type I interferon antagonist." Journal of virology vol. 87,22 (2013): 12489-95. doi:10.1128/JVI.01845-13
MERS-CoV ORF4a	STAT1	Emmie de Wit, Neeltje van Doremalen, Darryl Falzarano, and Vincent J Munster. Sars and mers: recentinsights into emerging coronaviruses.Nature reviews. Microbiology, 14(8):523—534, August 2016
MERS-CoV ORF4a	IRF9	Emmie de Wit, Neeltje van Doremalen, Darryl Falzarano, and Vincent J Munster. Sars and mers: recentinsights into emerging coronaviruses.Nature reviews. Microbiology, 14(8):523—534, August 2016
MERS-CoV ORF4a	STAT2	Emmie de Wit, Neeltje van Doremalen, Darryl Falzarano, and Vincent J Munster. Sars and mers: recentinsights into emerging coronaviruses.Nature reviews. Microbiology, 14(8):523—534, August 2016
MERS-CoV ORF4b	TBK1	Yang, Y. et al. Middle East respiratory syndrome coronavirus ORF4b protein inhibits type I interferon production through both cytoplasmic and nuclear targets. Sci. Rep. 5, 17554 (2015).
MERS-CoV ORF4b	IRF3	Yang, Y. et al. Middle East respiratory syndrome coronavirus ORF4b protein inhibits type I interferon production through both cytoplasmic and nuclear targets. Sci. Rep. 5, 17554 (2015).
MERS-CoV ORF4b	IRF7	Yang, Y. et al. Middle East respiratory syndrome coronavirus ORF4b protein inhibits type I interferon production through both cytoplasmic and nuclear targets. Sci. Rep. 5, 17554 (2015).
MERS-CoV ORF4b	IRF9	Emmie de Wit, Neeltje van Doremalen, Darryl Falzarano, and Vincent J Munster. Sars and mers: recentinsights into emerging coronaviruses.Nature reviews. Microbiology, 14(8):523—534, August 2016
SARS-CoV nsp1	STAT1	Wathelet, M. G., Orr, M., Frieman, M. B. & Baric, R. S. Severe acute respiratory syndrome coronavirus evades antiviral signaling: role of nsp1 and rational design of an attenuated strain. J. Virol. 81, 11620–11633 (2007).
SARS-CoV nsp1	STAT2	Wathelet, M. G., Orr, M., Frieman, M. B. & Baric, R. S. Severe acute respiratory syndrome coronavirus evades antiviral signaling: role of nsp1 and rational design of an attenuated strain. J. Virol. 81, 11620–11633 (2007).
SARS-CoV ORF3b	MAVS	Emmie de Wit, Neeltje van Doremalen, Darryl Falzarano, and Vincent J Munster. Sars and mers: recentinsights into emerging coronaviruses.Nature reviews. Microbiology, 14(8):523—534, August 2016
SARS-CoV ORF3b	MDA5	Emmie de Wit, Neeltje van Doremalen, Darryl Falzarano, and Vincent J Munster. Sars and mers: recentinsights into emerging coronaviruses.Nature reviews. Microbiology, 14(8):523—534, August 2016
SARS-CoV ORF3b	RIG-I	Emmie de Wit, Neeltje van Doremalen, Darryl Falzarano, and Vincent J Munster. Sars and mers: recentinsights into emerging coronaviruses.Nature reviews. Microbiology, 14(8):523—534, August 2016
SARS-CoV M protein	NF-κB	Fang, Xiaonan et al. "The membrane protein of SARS-CoV suppresses NF-κB activation." Journal of medical virology vol. 79,10 (2007): 1431-9. doi:10.1002/jmv.20953
SARS-CoV M protein	IRF3	Siu KL, Kok KH, Ng MH, et al. Severe acute respiratory syndrome coronavirus M protein inhibits type I interferon production by impeding the formation of TRAF3.TANK.TBK1/IKKepsilon complex. J Biol Chem. 2009;284(24):16202-16209. doi:10.1074/jbc.M109.008227
SARS-CoV N protein	MAVS	Emmie de Wit, Neeltje van Doremalen, Darryl Falzarano, and Vincent J Munster. Sars and mers: recentinsights into emerging coronaviruses.Nature reviews. Microbiology, 14(8):523—534, August 2016
SARS-CoV N protein	MDA5	Emmie de Wit, Neeltje van Doremalen, Darryl Falzarano, and Vincent J Munster. Sars and mers: recentinsights into emerging coronaviruses.Nature reviews. Microbiology, 14(8):523—534, August 2016
SARS-CoV N protein	IRF3	Emmie de Wit, Neeltje van Doremalen, Darryl Falzarano, and Vincent J Munster. Sars and mers: recentinsights into emerging coronaviruses.Nature reviews. Microbiology, 14(8):523—534, August 2016
SARS-CoV N protein	RIG-I	Emmie de Wit, Neeltje van Doremalen, Darryl Falzarano, and Vincent J Munster. Sars and mers: recentinsights into emerging coronaviruses.Nature reviews. Microbiology, 14(8):523—534, August 2016

Supplementary Note 11: IMSP algorithm

Algorithm 1 IMSP algorithm

Input : Pairwise similarity matrices M, interaction relationships R, relevant biological metadata D
Output: Type for each Edge (Interaction) $I_{i,j}$

```
// Stage 1: network construction and representation learning
```

G = constructNetwork(M, R, D) `// construct the unweighted network` G

`// Text2vec is a Word2vec-based sentence embedding model`

$Text2vec(V)$ = Text2vec.train(G.nodes) `// Pre-train node content embedding`

$Text2vec(I)$ = Text2vec.train(G.pos_and_neg_edges) `// Pre-train edge content embedding`

for V_i *in* V **do**
  $R_i^C = Text2vec(V)$.get(V_i) `// Get the content embedding for Node` V_i
end
for $I_{i,j}$ *in* I **do**
  if V_i *and* V_j *are protein homologs* **then**
    $w_{i,j}$ = getSequenceSimilarity(V_i, V_j) `// assign sequence similarity by BLASTp as edge weight`
  else
    $w_{i,j} = \sigma(TS\text{-}SS_{i,j}/\overline{TS\text{-}SS})$ `// assign` $TS\text{-}SS_{i,j}$ `similarity, which takes input` R_i^C `and` R_j^C`, as edge weight. Refer to Eq.(1)-(4)`
  end
end

$Node2vec(V)$ = Node2vec.train(G) `// Pre-train structural embeddings on weighted graph` G

IE = combine($Node2vec(V)$, $Text2vec(I)$) `// Generate final edge embeddings. Refer to Eq.(7)`

```
// Stage 2: edge classification
```

IE_{train} = getTrainingIE(G) `// Get representations for training edges`

$Label_{train}$ = getTrainingLabels(G) `// Get labels for training edges`

clf = MLP.train(IE_{train}, $Label_{train}$)

return clf.predict() `// Return predictions`

References

[S1] Tomoh Matsumiya and Diana M Stafforini. Function and regulation of retinoic acid-inducible gene-i. *Critical Reviews™ in Immunology*, 30(6), 2010.
[S2] Hilario J Ramos and Michael Gale Jr. Rig-i like receptors and their signaling crosstalk in the regulation of antiviral immunity. *Current opinion in virology*, 1(3):167–176, 2011.
[S3] Ting Liu, Lingyun Zhang, Donghyun Joo, and Shao-Cong Sun. Nf-κb signaling in inflammation. *Signal transduction and targeted therapy*, 2(1):1–9, 2017.
[S4] Laurie Kilpatrick and Mary Catherine Harris. Cytokines and inflammatory response in the fetus and neonate. In *Fetal and neonatal physiology*, pages 1555–1572. Elsevier, 2004.
[S5] Agata Michalska, Katarzyna Blaszczyk, Joanna Wesoly, and Hans AR Bluyssen. A positive feedback amplifier circuit that regulates interferon (ifn)-stimulated gene expression and controls type i and type ii ifn responses. *Frontiers in immunology*, 9:1135, 2018.
[S6] Lionel B Ivashkiv and Laura T Donlin. Regulation of type i interferon responses. *Nature reviews Immunology*, 14(1):36–49, 2014.
