# Supplementary material for: Network-based virus-host interaction prediction with application to SARS-CoV-2
Source: Patterns (N Y). 2021 Mar 29;2(5):100242. doi: 10.1016/j.patter.2021.100242 (PMC8006187; doi:10.1016/j.patter.2021.100242)
Supplement: Document S2. Article plus supplemental information [file mmc4.pdf]

Article

Patterns

Network-based virus-host interaction prediction with application to SARS-CoV-2

Highlights

- We built a virus-host interaction network with 7 human coronaviruses and 17 hosts
- We developed an ML-based method to predict protein- and organism-level interactions
- We revealed five potential infection targets of SARS-CoV-2
- We predicted 19 highly possible interactions between SARS-CoV-2 and human proteins

Authors

Hangyu Du, Feng Chen, Hongfu Liu, Pengyu Hong

Correspondence

hongfuliu@brandeis.edu

In brief

Given a new virus, our method can utilize existing knowledge and data about other highly relevant viruses to predict multi-scale interactions between the new virus and potential hosts.

Du et al., 2021, Patterns *2*, 100242
May 14, 2021 © 2021 The Authors.
https://doi.org/10.1016/j.patter.2021.100242

CellPress

Patterns

CellPress
OPEN ACCESS

Article

Network-based virus-host interaction prediction with application to SARS-CoV-2

Hangyu Du,[1,2] Feng Chen,[1,2] Hongfu Liu,[1,3,*] and Pengyu Hong[1,3]
[1]Department of Computer Science, Brandeis University, Waltham, MA 02453, USA
[2]These authors contributed equally
[3]Lead contact
*Correspondence: hongfuliu@brandeis.edu
https://doi.org/10.1016/j.patter.2021.100242

THE BIGGER PICTURE SARS-CoV-2, a novel single-stranded RNA coronavirus causing COVID-19, is mounting an unprecedented threat against our society and the world. Although tremendous efforts have been devoted into SARS-CoV-2 research, most of them either focused on a few proteins or only provided high-level overviews. Deeper and more comprehensive analyses are needed to shed new light onto the molecular mechanisms underlying the COVID-19 pandemic. Moreover, there is a massive amount of data and knowledge about highly relevant RNA viruses which have yet to be fully utilized.

In this work, we constructed a multi-layer virus-host interaction network to incorporate these data and knowledge. We developed a machine-learning-based method to predict virus-host interactions at both protein and organism levels. Our approach revealed five potential infection targets of SARS-CoV-2 and 19 highly possible interactions between SARS-CoV-2 proteins and human proteins in the innate immune pathway.

1 2 **3** 4 5 **Development/Pre-production:** Data science output has been rolled out/validated across multiple domains/problems

SUMMARY

COVID-19, caused by Severe Acute Respiratory Syndrome Coronavirus 2 (SARS-CoV-2), has quickly become a global health crisis since the first report of infection in December of 2019. However, the infection spectrum of SARS-CoV-2 and its comprehensive protein-level interactions with hosts remain unclear. There is a massive amount of underutilized data and knowledge about RNA viruses highly relevant to SARS-CoV-2 and proteins of their hosts. More in-depth and more comprehensive analyses of that knowledge and data can shed new light on the molecular mechanisms underlying the COVID-19 pandemic and reveal potential risks. In this work, we constructed a multi-layer virus-host interaction network to incorporate these data and knowledge. We developed a machine-learning-based method to predict virus-host interactions at both protein and organism levels. Our approach revealed five potential infection targets of SARS-CoV-2 and 19 highly possible interactions between SARS-CoV-2 proteins and human proteins in the innate immune pathway.

INTRODUCTION

Severe Acute Respiratory Syndrome Coronavirus-2 (SARS-CoV-2), a novel virus causing the COVID-19 disease, was first reported in Wuhan, China, in December of 2019. Since then, it has quickly become a global health crisis[1] with over 50 million people infected and over 1,250,000 deaths across 200 countries by November 2020.[2] The impact of SARS-CoV-2 has significantly surpassed previous outbreaks of coronaviruses, such as Severe Acute Respiratory Syndrome Coronavirus (SARS-CoV) in 2003 and the Middle East Respiratory Syndrome Coronavirus (MERS-CoV) in 2012. Besides humans, SARS-CoV-2 has been confirmed to infect several other mammals closely related to human activities, including dogs,[3] cats,[4] tigers,[5] rats,[6] and golden Syrian hamsters.[7] Also, there is a high possibility for infected animals to transmit and spread the virus to humans.[8] It is important to identify a comprehensive set of such mammals because they can potentially serve as covert means to exacerbate the spread of COVID-19. Moreover, identifying interactions between SARS-CoV-2 proteins and host proteins can deepen our understanding

Check for updates

This is an open access article under the CC BY license (http://creativecommons.org/licenses/by/4.0/).

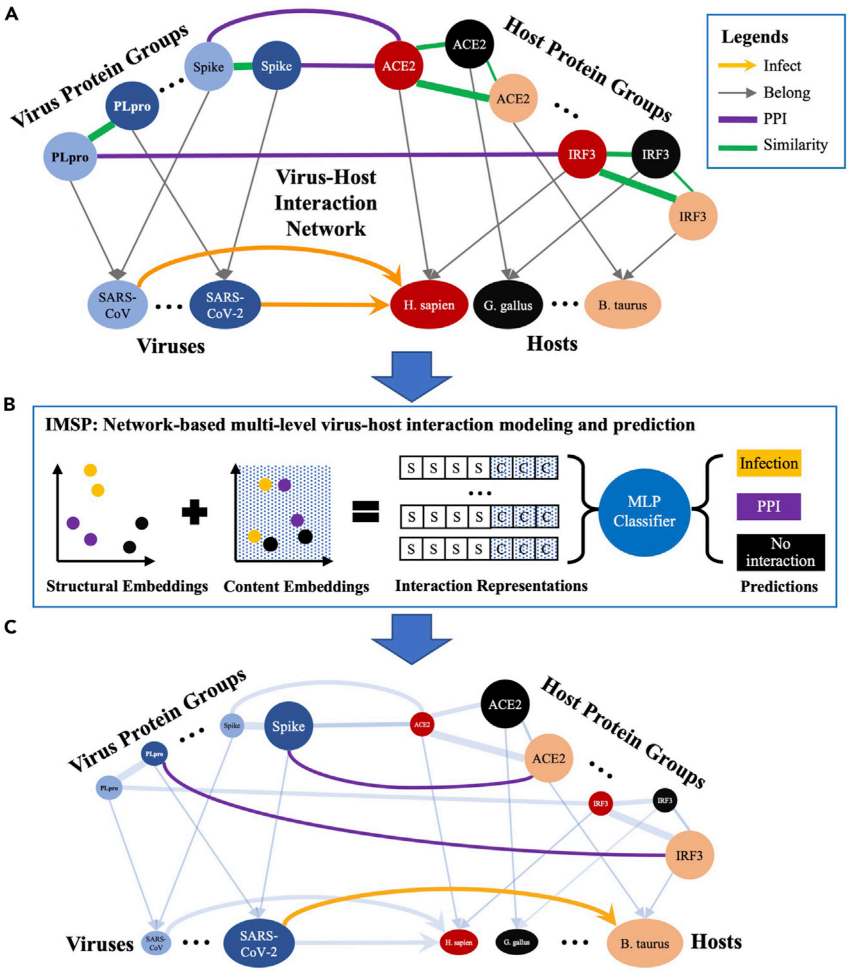

Figure 1. Infection mechanism and spectrum prediction

(A) The virus-host interaction network. Nodes represent proteins, viruses, and hosts; edges represent relationships (i.e., PPI, infection, protein-homolog similarity, and organism-protein belonging). The color of a node indicates its organism. The thickness of a protein-homolog similarity edge indicates its level of similarity. For the full network, refer to the viral entry graph (Figure S3), interferon signaling pathway graph (Figure S4), and infection graph (Figure S5).

(B) IMSP learns a representation for each potential edge, which contains a structural embedding and a content embedding. The structural embedding captures the local structural features of an edge. The content embedding captures the attributes that reveal biological aspects of an edge. The representation of each edge is derived by concatenating its structural and content embeddings, where S stands for a structural embedding element and C stands for a content embedding element. A Multi-layer Perceptron (MLP) is trained to take the edge representations as input and reports negative (non-connected) edges whose corresponding edge representations are classified as infection or PPI. Note that no-interaction is also a potential class for the classification task. See experimental procedures for calculation of the structural and content embeddings.

(C) Exemplar predicted edges are highlighted and colored accordingly to their types. Existing edges are dimmed.

of the viral invasion processes and may help design treatments and vaccines. In general, we want to promptly achieve the above two goals for new zoonotic viruses, which we believe can be done by leveraging the knowledge and data about known viruses highly relevant to the new ones.

The research community has accumulated a great deal of knowledge about several other human coronaviruses (including SARS-CoV,[9–16] HCoV-HKU1,[14] HCoV-OC43,[17,18] HCoV-NL63,[19] and MERS-CoV)[20–24] and has collected a large amount of data about them. For example, it was shown that human angiotensin-converting enzyme 2 (ACE2) was the primary host receptor used by the S protein (S-protein) of SARS-CoV-2 for the virus to gain entry into human cells[25] (Figure S1). ACE2 is also the host receptor used by SARS-CoV[13] and HCoV-NL63.[19] The S-protein of SARS-CoV-2 binds significantly tighter to ACE2 than its counterpart in SARS-CoV.[26] After the virus enters host cells, interferon-stimulated genes are essential for a host to defend against viral infection (Figure S2). This knowledge and data can be utilized to investigate the infection spectrum of SARS-CoV-2 and its interactions with hosts at the protein level. Using this information, we have built a virus-host interaction network of 7 viruses and 17 hosts that summarizes the existing protein-protein interaction (PPI) and infection relationships among them (Figure 1A; for more details, see Figures S3–S5 and Tables S1, S2, S3, and S4).

We have developed a network-based multi-level virus-host interaction modeling and prediction, termed infection mechanism and spectrum prediction (IMSP) (Figure 1B; for details, see experimental procedures), which uses machine-learning techniques to learn from the constructed virus-host interaction network and predict novel virus-host interactions at both the protein (i.e., Mechanism) and organism (i.e., Spectrum) levels. IMSP predicts that the SARS-CoV-2 S-protein can bind well with ACE2 receptors in five mammalian hosts, which have not been reported. Among those hosts, five are predicted to have high risks of being infected by SARS-CoV-2. Moreover, IMSP identifies 19 new interactions between SARS-CoV-2 proteins and human proteins in the innate immune pathway. To our best knowledge, our work is the first to apply machine-learning techniques for predicting virus-host interactions at both protein and organism levels. Previous works[27,28] only focused on the relationships between SARS-CoV-2 proteins and human proteins and ignored other hosts that might be infected by SARS-CoV-2.

RESULTS

Here we explain the structure of our virus-host interaction network, highlight the predicted interactions of SARS-CoV-2, and present the link prediction performance evaluation of our model IMSP. We built our network with two layers (an organism layer and a protein layer). The organism layer consisted of 7 human coronaviruses and 17 mammalian hosts. Those hosts are either close to human

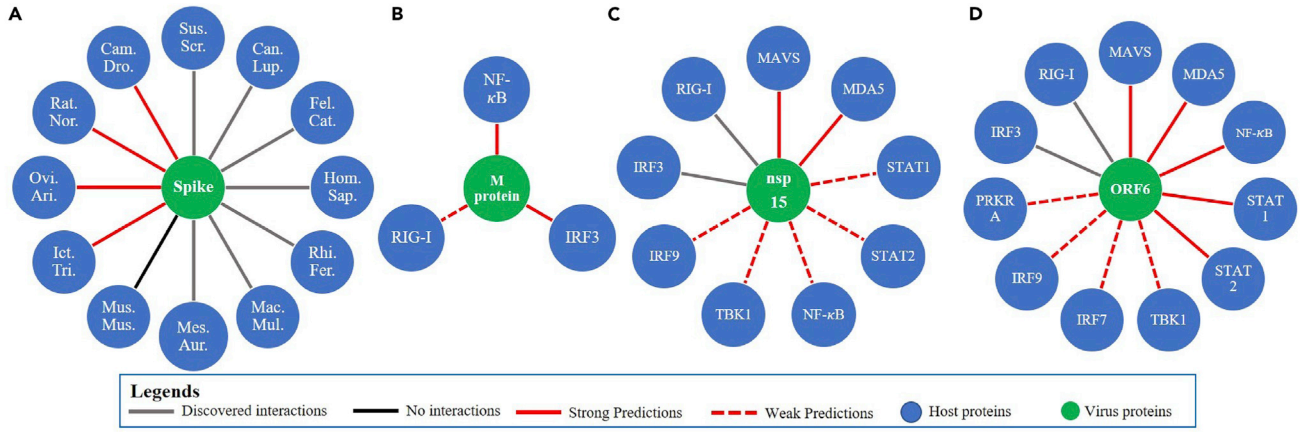

Figure 2. PPIs prediction for SARS-CoV-2

(A) The known and predicted bindings between the S-protein in SARS-CoV-2 and ACE2 in mammalian hosts. Host names are displayed in their abbreviation form: Hom.Sap., *Homo sapiens*; Mus.Mus., *Mus musculus*; Fel.Cat., *Felis catus*; Can.Lup., *Canis lupus familiaris*; Ovi.Ari., *Ovis aries*; Rat.Nor., *Rattus norvegicus*; Mac.Mul., *Macaca mulatta*; Rhi.Fer., *Rhinolophus ferrumequinum*; Mes.Aur., *Mesocricetus auratus*; Bos.Tau., *Bos taurus*; Ict.Tri., *Ictidomys tridecemlineatus*; Cam.Dro., *Camelus dromedarius*; Sus.Scr., *Sus scrofa domesticus*.

(B–D) The known and predicted interactions of M protein (B), nsp15 (C), and ORF6 (D) in SARS-CoV-2 with proteins in the human IFN signaling pathway that contribute to IFN signaling pathway suppression.

activities or proven to be infected by some human coronaviruses in our network. The protein layer contained 10 virus proteins and 13 host proteins. The proteins were selected based on two primary considerations: proteins involved in viral entry and the interferon (IFN) signaling pathway, both of which are critical to a successful virus infection. The virus needs to enter the host cells through the receptors on the membrane, and the binding ability between the S-protein of the virus and the host receptor determines the success of such viral entry. The suppression ability on the IFN signaling pathway of the virus negatively affects the efficiency and the effectiveness of the response of the innate immune system, which would allow the virus to rapidly replicate and spread among cells. IMSP performed a network-based representation learning to integrate information about virus-host infections, PPIs, organism-protein belongings, and similarities between protein homologs. This produced comprehensive representations and a neural-network-based classifier for accurately predicting novel viral infection and interactions between virus proteins and host proteins.

SARS-CoV-2-host multiple-type interaction predictions

We applied IMSP on SARS-CoV-2 and six other human coronaviruses to obtain high-confidence predictions of PPIs and infections. Figure S1 shows the mechanism of the binding of S-proteins and host receptor ACE2. Figure S2 shows the interactions between virus proteins and host proteins involved in the IFN pathway. Figure S3 shows the S-protein binding subnetwork. Figure S4 shows the innate immune pathway subnetwork. Figure S5 shows the organism layer. Tables S1 and S2 show the complete node and linkage information of the virus-host network. All infection predictions are shown in Table S3, and PPI predictions are presented in Table S4.

SARS-CoV-2 S-protein binding predictions

The binding ability of the S-protein of SARS-CoV-2 with the host ACE2 receptors is a key factor deciding the infection capability of SARS-CoV-2. IMSP predicted that the S-protein of SARS-CoV-2 could have a high probability of binding well with the ACE2 receptors in rats, sheep, camels, and squirrels (Figure 2A).

Rats were recognized to be susceptible to several other human coronaviruses, such as SARS-CoV,[9] MERS-CoV,[29] HCoV-OC43,[18] and HCoV-HKU1.[30,31] It is highly possible that rats could still be the potential host for SARS-CoV-2.

The overall similarity of ACE2 for the squirrel, sheep, and camel is 91.82%, 90.81%, and 92.42%, respectively compared with human ACE2. These predictions still require more practical research to determine the binding affinity between the S-protein of SARS-CoV-2 with ACE2s on these mammals. It was shown that ACE2 could tolerate up to seven amino acid changes out of 20 critical ones that contact with the S-protein without losing the functionality as the target receptor[32] for SARS-CoV-2. This means that sequence similarity might not be the only factor that influences the binding affinity between the ACE2 receptor and the S-protein of SARS-CoV-2.

SARS-CoV-2 and human interferon pathway interactome prediction

The IFN pathway plays a critical role in the human immune response. After the virus infection is detected, the innate immune system will induce IFN signaling, and the expression of IFN genes will increase the cellular resistance to viral invasion. Viruses have developed various strategies to inhibit IFN signaling to facilitate successful viral invasion.[33] SARS-CoV and MERS-CoV were studied quite comprehensively in terms of counteracting the IFN signaling responses compared with SARS-CoV-2. From IMSP, 19 interactions between SARS-CoV-2 proteins and human proteins in the innate immune pathway were identified, shown in Figures 2B–2D. These PPIs had a high probability of playing crucial roles in the suppression of the innate immune system response of the host.

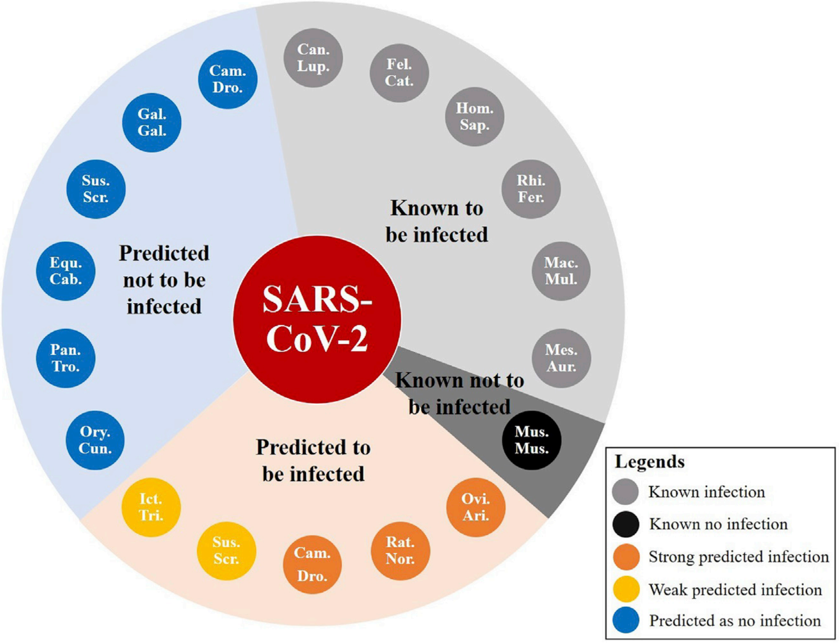

Figure 3. Infection prediction for SARS-CoV-2
This figure shows all 17 mammalian hosts in our network and their infection relationships with SARS-CoV-2.

Membrane (M) protein not only serves as the protein in virus to bind to all other structural proteins[34] but also is found to inhibit IFN production in SARS-CoV[35] and MERS-CoV.[24] From IMSP prediction, it was highly possible that M protein in SARS-CoV-2 could interact with nuclear factor kappa-light-chain-enhancer of activated B (NF-κB), interferon regulatory factor 3 (IRF3), and retinoic acid-inducible gene I (RIG-I).

Open reading frame protein 6 (ORF6) and non-structural protein 15 (nsp15) in SARS-CoV-2 were discovered to be crucial viral IFN antagonists of SARS-CoV-2. From previous research, we knew that these two proteins inhibit the localization of IRF3 by interacting with RIG-I.[36] A similar function was found for ORF6 in SARS-CoV.[37] ORF6 and nsp15 in SARS-CoV were proved to interact with signal transducer and activator of transcription 1 (STAT1) and STAT2.[38] From predictions made by IMSP (shown in Figures 2C and 2D), ORF6 and nsp15 in SARS-CoV-2 were suggested to have potential interactions with melanoma differentiation-associated protein 5 (MDA5), mitochondrial anti-viral-signaling protein (MAVS), STAT1, STAT2, NF-κB, IRF9, and TANK binding kinase 1 (TBK1). Since MAVS works as the adaptor molecule for MDA5,[39] it is possible that a viral protein that interacts with either one of these two would also interact with the other. Besides these, ORF6 was also predicted to interact with protein kinase interferon-inducible double-stranded RNA-dependent activator (PRKRA) and IRF7. As nsp15 and ORF6 both function in nuclear transport machinery after viral entry,[27] it is reasonable that, for these two proteins, similar interactions with innate immune pathways are predicted. Careful experiments should be conducted to identify the impact of nsp15 and ORF6 on the innate immune system.

SARS-CoV-2 infection prediction

Based on both the protein-level and organism-level interaction predictions, we concluded five highly possible infection predictions for SARS-CoV-2. These mammals were predicted to be susceptible to SARS-CoV-2 in the organism layer. They were also proved or predicted to have a successful spike-receptor binding between the S-protein of SARS-CoV-2 and their own ACE2 receptors. As shown in Figure 3, these animals included rats, sheep, camels, swine, and squirrels.

Rat was identified as a host for all beta-coronaviruses: SASR-CoV,[9] MERS-CoV,[29] HCoV-OC43,[18] and HCoV-HKU1.[30,31] SARS-CoV-2 also falls into the category of beta-coronavirus,[40] which has a high possibility of infecting rats.

Swine's ACE2 was identified to be able to bind with the S-protein of SARS-CoV-2,[41] and our model predicted that swine could be successfully infected after the receptor binding. This is also supported by recent research on swine.[42]

Camels are hosts for MERS-CoV.[22] This means that camels can also be hosts for other coronaviruses. Camels, along with sheep and squirrels, are closely related to the human living environment or daily diet. They could be potential mammalian hosts that again transmit the virus back to human society. The investigation of these highly possible infections could potentially help identify the transition path of the virus and further control the transmission of SARS-CoV-2 from and between mammalian hosts. Further research on these potential hosts might be crucial to social health and safety.

Interaction prediction performance evaluation

Many machine-learning and graph-embedding methods have been developed and applied to various applications.[43–48] In this work, we compared IMSP with five other baseline models on our dataset in a 5-fold stratified cross-validation setting. The baseline models include two famous random-walk-based models (DeepWalk[43] and Node2vec),[45] two neural-network-based models (Large-scale Information Network Embedding [LINE][44] and Structural Deep Network Embedding [SDNE]),[46] and a classical matrix-based model, Graph Factorization GF.[49] For the stratified cross-validation experiment, we created a sampling strategy to ensure that the training subset in each cross-validation run can form a fully connected network. Such a fully connected network could ensure that our network structural embedding model embedded nodes into the same vector space. To ensure the balance of input data, we gathered negative (non-connected) edges in addition to positive (connected) edges that already existed in each fold. We sampled negative edges from two directions: known negatives (i.e., true negatives) and unknown negatives. We considered spike-receptor interactions demonstrated as nonexistent as known negatives, such as the one between the S-protein of SARS-CoV-2 and the host receptor

CellPress
OPEN ACCESS

Table 1. Link prediction: Overall performance evaluation and comparison

Model	Accuracy	Weighted precision	Weighted recall	Weighted F1-score	AUC macro	AUC weighted
GF[49]	0.879 ± 0.008	0.852 ± 0.011	0.879 ± 0.008	0.863 ± 0.009	0.913 ± 0.007	0.944 ± 0.006
Deepwalk[43]	0.894 ± 0.008	0.870 ± 0.010	0.894 ± 0.008	0.879 ± 0.009	0.926 ± 0.010	0.952 ± 0.007
LINE[44]	0.742 ± 0.026	0.727 ± 0.030	0.742 ± 0.026	0.732 ± 0.029	0.874 ± 0.021	0.881 ± 0.023
Node2vec[45]	0.902 ± 0.007	0.868 ± 0.007	0.902 ± 0.007	0.883 ± 0.007	0.896 ± 0.011	0.932 ± 0.010
SDNE[46]	0.820 ± 0.015	0.791 ± 0.019	0.820 ± 0.015	0.799 ± 0.017	0.904 ± 0.012	0.930 ± 0.011
IMSP	0.971 ± 0.005	0.972 ± 0.006	0.971 ± 0.005	0.971 ± 0.006	0.997 ± 0.001	0.996 ± 0.001

AUC, area under the receiver-operating characteristic curve. This table presents six evaluation metrics regarding the link prediction performance of our model compared with five other baseline models. While evaluating performance, we followed 5-fold stratified cross-validation setting with shuffle enabled. This method preserved the percentage of samples for each class (i.e., type of edge) in each fold. We created a sampling strategy to ensure that the training subset in each cross-validation run can form a fully connected network. To ensure the balance of input data, we gathered negative (non-connected) edges in addition to positive (connected) edges that already existed in each fold. While sampling negative edges, we randomly selected some from known negative edges (i.e., true negatives), which consisted of spike-receptor interactions demonstrated as nonexistent. We randomly selected the remaining negative edges from other non-connected node pairs, which we assumed did not exist. These negative edges were then added to each fold to match the number of positive edges. We performed this 5-fold stratified cross-validation experiment for 30 runs. In each run, we would generate a new 5-fold split. We then performed two-sample heteroscedastic t tests for these six overall performance evaluation metrics to test the significance of IMSP improvement. Lastly, we reported the average with SD for each metric.

dipeptidyl peptidase 4 (DPP4; the target host receptor of MERS-CoV). Since we still lacked a comparable amount of negative edges, we randomly selected non-connected node pairs as negative edges, which we assumed as not existing. We added these negative samples into each fold to match the number of positive samples. We then evaluated IMSP and other models under the 5-fold stratified cross-validation setting as described above. We repeated the cross-validation experiment for 30 independent runs. In each run, we generated a new 5-fold split. Finally, we performed a two-sample heteroscedastic t test at the 0.01 significance level to test the significance of our model's improvement against other models.

Table 1 shows the performance comparison measured in six common link prediction evaluation metrics. IMSP achieved an overall link prediction accuracy of 97.1% with a standard deviation (SD) of 0.005, which demonstrated a 7.7% gain compared with the second-best model. Our model also excelled in its weighted F1-score, achieving 0.971 with SD of 0.006, which exceeded the second-best model by 10.0%. The p values for these two metrics were all smaller than 0.01, which indicated significant improvement for our model. We also presented the performance on infection and PPI predictions (Figure 4). IMSP achieved an F1-score of 0.854 with a 0.090 SD for infection predictions, a 40.4% increase compared with the second-best model. The p value was smaller than 0.01, indicating a significant improvement in our model. For PPI predictions, our model achieved an F1-score of 0.867 with 0.034 SD, a 1.6% increment compared with the second-best model. The p value also demonstrated a significant improvement for IMSP under the 0.01 significance level. In conclusion, our model showed statistically significant improvements compared with all existing models in 11 of 12 evaluation metrics.

The high performance of IMSP might result from its ability to take full advantage of well-studied knowledge and data from previous biology research with protein-level variations. Thanks to the novel design of our virus-host interaction network, cross-organism information and multi-class linkage information can be well preserved. Another reason behind the performance improvement of IMSP is that it factors essential biological metadata for nodes into the learned representations of edges. This design substantially helped the classifier output a correct predicted class when formulating edge representations. However, around 10% of PPI predictions were unlikely predictions by our definition, i.e., PPIs between S-protein and non-receptor host proteins. To minimize unlikely predictions, we also utilized known negative edges (true negatives) in the protein layer to constitute part of the negative samples for training and testing. This finally reduced the unlikely PPI predictions to around 5%.

In conclusion, IMSP exhibited robust and stable performance in both top-level and detailed evaluation metrics, which was substantially improved compared with existing tools. When analyzing newly emerged viruses with limited available information, namely SARS-CoV-2, IMSP could provide reasonable and reliable predictions.

DISCUSSION

This study assembled 260 nodes and 1,995 known edges. Each node represented a virus/virus protein/host/host protein, and each edge represented a virus-host infection/PPI/protein-homolog similarity/organism-protein belonging. Based on this network, we predicted the potential host for viruses and undiscovered PPIs. Among all currently known seven human coronaviruses, SARS-CoV and MERS-CoV were relatively well studied in terms of interactions (i.e., infection and PPI). However, interactions of HCoV-OC43, HCoV-NL63, HCov-HKU1, HCoV-229E, and the newly emerged SARS-CoV-2 remained relatively less discovered. Our model predicted 939 PPIs and 24 infections that were likely to happen. These predictions need further experiments for validation.

Established discoveries about the viral interactions with host proteins were scarce for SARS-CoV-2. However, SARS-CoV-2 was highly suspected of suppressing the innate immune response and reducing the production of IFN. Thus, the findings by IMSP could help discover the protein-level mechanism of virus invasion and host response to provide clues toward developing therapeutic strategies for the treatment of this disease. Some of our prediction results have been revealed as

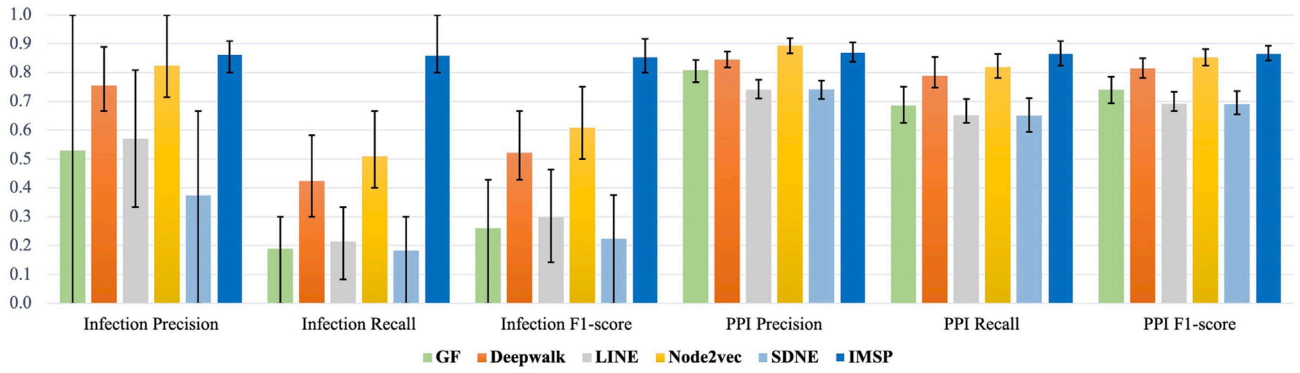

Figure 4. Performance on PPI and infection predictions

This figure demonstrates the performance of IMSP on PPI and infection predictions in comparison with five other baseline models. The ocean-blue columns represent the performance of IMSP derived from the average of 30 independent 5-fold stratified cross-validation runs. The error bars for each column mark the 25th and 75th percentile. Our IMSP model achieved 0.854 for the infection F1-score and 0.867 for the PPI F1-score. Compared with other models, our model outperformed them in all the evaluation metrics except in PPI Precision. Specifically, in terms of infection F1-score, our model outperformed the second-best model Node2vec[45] by 40.4%. In terms of PPI F1-score, our model also surpassed the second-best model Node2vec[45] by 1.6%.

meaningful. It should be noted that, during the review period, two of our prediction results were validated in wet-lab experiments by independent labs,[42,50] which demonstrated that swine is susceptible to SARS-CoV-2 and that the M protein of SARS-CoV-2 inhibits IFN production by targeting RIG-I/MDA-5 signaling.

More broadly, IMSP could be applied to any other analysis of the virus-host interaction network predictions. IMSP would build the network based on the information of the PPIs, protein-homolog similarities, virus-host infection relations, and related protein function knowledge if available. Based on such a network, IMSP could predict high-possibility PPIs and infections. We hope to use this pipeline as a guideline for investigating various similar viruses and their mechanisms with hosts on both organism level and protein level.

Limitations of the study

This section discusses the limitation of our work in terms of prediction validation, quality of data sources, model bias, and potential improvements. Concerning prediction validation, ideally wet-lab experiments should be conducted to validate our predictions, which require special facilities not commonly available. Thus, we were unable to validate our predictions through biological experiments. We collected protein sequences, infection relationships, and known PPIs from the best available data sources when carrying out this study. The quality, errors, and uncertainty of these data sources could affect the performance of our approach. This may harm the reliability of our predictions, and hence biologists should exercise extra caution when using our predictions to aid the design of experiments. Our approach may suffer from sampling bias, representation bias, and population bias.[51] For example, we only included the proteins known to play crucial roles in viral entry and the IFN signaling pathway. It is possible that some related proteins were ignored, i.e., our model potentially carries sampling bias. Our model might also suffer from representation bias due to missing protein sequences, which could lead to non-uniform protein representation in different mammalian hosts in our network. Additionally, we could not include some mammals (e.g., rabbits and civets) because most of their protein sequences are either unavailable or of low quality in the National Center for Biotechnology Information (NCBI) database, which led to population bias. As more data become available, a more comprehensive network could be constructed by our IMSP model, which would substantially mitigate the model bias. Lastly, the model can also be improved by incorporating gene set enrichment and sequence motif analysis.

EXPERIMENTAL PROCEDURES

Resource availability

Lead contact

Further information and requests for code and data should be directed to and will be fulfilled by the lead contact, Hongfu Liu (hongfuliu@brandeis.edu).

Materials availability

This study did not generate any physical materials.

Data and code availability

All data and codes are available at Github repositories. IMSP model, its predictions, and performance evaluations can be found at https://github.com/hangyu98/IMSP; data and parsing code can be found at https://github.com/hangyu98/IMSP-Parser. Additional supplemental items are available from Mendeley Data at doi: 10.17632/3s2dr7y6s2.1.

Virus-host interaction network data selection

The virus-host interaction network consists of two layers (an organism layer and a protein layer). The organism layer contains a set of viruses (including SARS-CoV-2, SARS-CoV, HCoV-229E, HCoV-HKU1, HCoV-OC43, HCoV-NL63, and MERS-CoV) and a set of hosts (including human, mouse, rat, dog, cat, camel, squirrel, cattle, chimpanzee, red junglefowl, rabbit, horse, monkey, rat, sheep, swine, and golden Syrian hamster). At the protein layer, we focus on proteins that are known to be involved in viral invasion or immune system response and suppression. The network contains 13 host protein-homolog groups obtained from NCBI: ACE2, DPP4, IRF3, IRF7, IRF9, MAVS, MDA5, NF-κB, PRKRA, TBK1, RIG-I, STAT1, and STAT2. The virus proteins include homologs of S-protein, M protein, nucleocapsid protein, nsp1, nsp15, ORF3b, ORF4a, ORF4b, ORF6, and papain-like protease (PLpro). There are four types of edges in the network: PPI, virus-host infection, organism-protein belonging, and similarity relation between protein homologs. PPI and infection relationships are gathered from academic publications.[9–24] Organism-protein belonging and protein-homolog similarity relation are innately connected. Detailed PPI data resources are presented in Table S5.

Table 2. Notations

Notation	Description
V_i	node i in the network
V	the set of all nodes
$I_{i,j}$	edge between node i and node j
I	the set of all edges in the network
R_i^S	structure embedding vector for node i
R_i^C	content embedding vector for node i
$CE_{i,j}$	content embedding vector for edge $I_{i,j}$
$IE_{i,j}$	full edge embedding vector for edge $I_{i,j}$
$w_{i,j}$	edge weight for edge $I_{i,j}$
$ED_{i,j}$	Euclidean distance between node i and node j
$MD(R_i^C, R_j^C)$	magnitude difference between vector R_i^C and R_j^C
$TS-SS_{i,j}$	*TS-SS* similarity between vector R_i^C and R_j^C

Infection mechanism and spectrum prediction

Our IMSP model requests three inputs: pairwise similarity matrices (parsed from percentage of positives from NCBI BLASTp result) for protein homologs, a set of known PPIs and infections, and protein function data. Given these three inputs, the model constructs a heterogeneous two-layer virus-host interaction network. IMSP then performs graph representation learning and combines the structural embeddings with the content embeddings to form edge representations. Lastly, in the link prediction phase, IMSP trains a neural-network-based Multi-layer Perceptron (MLP) classifier on learned representations to perform multi-class classification task. Along with post-process procedures, our model outputs high-possibility undiscovered PPIs and infections. In the following, we elaborate on the two main steps of IMSP in terms of virus-host interaction network construction and representation learning, and virus-host interaction prediction. To show the design of our model, we present the pseudocode sample in Alg. 1 in supplemental information. The time complexity is $O(|V|^2)$ and the space complexity is $O(|V|^2)$. Please refer to Table 2 for notations.

Virus-host interaction network construction and representation learning

We utilized nodes to represent either organisms or proteins. Edges were used to represent PPI/infection/similarity/belonging relationships. To model the network, we constructed an undirected two-layer heterogeneous network using NetworkX.[52] The network carried four groups of nodes: host, host protein, virus, and virus protein. We organized the virus group and the host group into the organism layer. Similarly, host protein groups and virus protein groups were put into the protein layer. By nature, the network held four types of edges: PPI (between virus protein groups and host protein groups), infection (between virus group and host group), protein-homolog similarity relation (between virus/host protein homologs in protein layer), and organism-protein belonging relation (between organism layer and protein layer). Protein-homolog similarity and organism-protein belonging relationships were innately connected. PPIs and infections were connected based on proven molecular level knowledge or infection data from existing research.[3–24,53–55] After building the network, the virus-host interaction network contained 260 nodes and 1,995 edges. Intuitively, if there is an interaction edge (infection or PPI) between two nodes V_i and V_j, an edge with the same type (infection or PPI) is more likely to form between V_i and another node with high biological similarity to V_j. We therefore designed a method that assigns a weight to each relationship in the network. A structure embedding model[45] was then applied to factor in such information into the node representations, which is later used in predicting interactions between nodes. To be more specific, if a relationship connects two protein homologs, its weight is equal to the similarity between their full-length sequences. For other relationships, we calculated its weight as the similarity between the text content of the connected nodes. The text content of a node includes the name and molecular functions if a node represents a protein. The text content is processed by Text2vec, a Word2vec[56]-based model, to obtain the node content embedding denoted as R_i^C for V_i. We then utilized the *TS-SS* similarity metric,[57] a robust and reliable similarity measurement in the field of textual mining, to calculate $w_{i,j}$ as the *TS-SS* similarity between R_i^C and R_j^C. The technical details are explained below:

$$TS-SS_{i,j} = |R_i^C| \cdot |R_j^C| \cdot \sin(\theta') \cdot \theta' \cdot \pi \cdot \left(ED\left(R_i^C, R_j^C\right) + MD\left(R_i^C, R_j^C\right)\right)^2 \Big/ 720, \qquad \text{(Equation 1)}$$

where$MD(R_i^C, R_j^C)$[57] is defined as the magnitude difference between R_i^C and R_j^C, which is calculated as

$$MD\left(R_i^C, R_j^C\right) = \left| \sqrt{\sum_{n=1}^{\dim R_i^C} R_i^{C^2}} - \sqrt{\sum_{n=1}^{\dim R_j^C} R_j^{C^2}} \right|, \qquad \text{(Equation 2)}$$

and θ' is defined as

$$\theta' = \cos^{-1}\left(\cos\left(R_i^C, R_j^C\right)\right) + 10. \qquad \text{(Equation 3)}$$

Note that θ' is increased by 10° to overcome the problem of overlapping vectors. $w_{i,j}$ is then calculated as

$$w_{i,j} = \sigma\left(TS-SS_{i,j} \Big/ \overline{TS-SS}\right), \qquad \text{(Equation 4)}$$

where σ is the sigmoid function, and $\overline{TS-SS}$ denotes the average of $TS-SS_{i,j}$, for all i,j, if $i \neq j$ and $V_i, V_j \subset V$.

For graph representation learning, we captured the graph heterogeneity by adding the heterogeneous content information to its structural information. Specifically, we performed network structural embedding assuming the network is homogeneous. We then added the content embedding on top of structural embedding to model the heterogeneity.

First, for network structural embedding, we used a powerful network representation learning model, Node2vec,[45] to learn the structural embedding for nodes. Node2vec is a state-of-the-art model for homogeneous network embedding. We took full advantage of the biased searching algorithm offered by Node2vec during our application. Precisely, the Node2vec model performed a biased fixed-length random walk for graph sampling, which takes edge weight into account. Let c_m denote the mth node in walk with c_0 denoting the starting node of the current random walk. Nodes c_m are generated by the following distribution:

$$P(c_m = V_i \mid c_{m-1} = V_j) = \begin{cases} \pi_{V_j,V_i}/Z & \text{if } I_{i,j} \subset I \\ 0 & \text{otherwise} \end{cases}, \qquad \text{(Equation 5)}$$

where $m \ngeq 1$, Z is the normalizing constant, and π_{V_j,V_i} is the unnormalized transition probability between V_j and V_i, which is calculated as $\pi_{V_j,V_i} = \alpha_{pq}(V_t, V_i) \cdot w_{i,j}$. Note that the edge weight $w_{i,j}$ is taken into consideration. Assume we have just transitioned from V_t to V_j and are now evaluating the transition probability leaving V_j. Let V_i represents the set of all neighbors of V_j. $\alpha_{pq}(V_t, V_i)$, termed as search bias, is calculated as

$$\alpha_{pq}\left(V_t, V_i\right) = \begin{cases} 1/p & \text{if } d_{V_t,V_i} = 0 \\ 1 & \text{if } d_{V_t,V_i} = 1 \\ 1/q & \text{if } d_{V_t,V_i} = 2 \end{cases}, \qquad \text{(Equation 6)}$$

where d_{V_t,V_i} denotes the shortest path between V_t and V_i.

In Equation 6, p (return hyperparameter) and q (in-out hyperparameter) are the two crucial hyperparameters of Node2vec. They can be adjusted to influence the probability of going back to V_i after visiting V_j and the probability of exploring the undiscovered components of the network. In this way, we were able to tune the hyperparameters of the structural embedding model, Node2vec, through a grid search algorithm to generate the structural embeddings.

Second, to generate edge content embeddings, i.e., $CE_{i,j}$ for all possible $I_{i,j}$, we combined the textualized node content (including name, group, layer, and function) of V_i and V_j with expected edge type such as PPI/infection/protein-homolog similarity/organism-protein belonging. We then input such text into

Text2vec, a Word2vec[56]-based model, to generate edge content embeddings. The full edge representations that consider both structural and content information for edge $l_{i,j}$ are formulated as follows:

$$IE_{i,j} = \left[R_i^S, R_j^S, CE_{i,j}\right], \quad IE_{j,i} = \left[R_j^S, R_i^S, CE_{j,i}\right]. \quad \text{(Equation 7)}$$

Note that by the nature of Text2vec, the order of input document does not affect its output, meaning that $CE_{i,j}$ is the same as $CE_{j,i}$. Upon finishing this step, we obtained all edge representations, $IE_{i,j}$, for all V_i and $V_j \subset V$ and $i \neq j$.

Virus-host interaction prediction

In the interaction prediction phase, we utilized a neural-network-based classification model, MLP classifier, provided by scikit-learn[58] to perform multi-class classification. The classifier would classify edges into infection, PPI, no-interaction, organism-protein belongings, and similarity relations between protein homologs, using the learned edge representations. The predicted interactions (i.e., infection and PPI) would go through a post-processing step to eliminate unlikely interaction predictions. The processed result would be the output of IMSP.

Here we performed 5-fold stratified cross-validation. While splitting data into folds, we let each fold have roughly the same percentage of interactions in each interaction type. Besides, each fold has the same number of positive (i.e., known interactions) and negative (i.e., non-interaction) samples. It should be noted that the negatives consist of both validated non-interactions (e.g., the S-protein of SARS-CoV-2 is known not to bind well to the human ACE2 receptor) and other non-interactions that have yet to be validated experimentally. To mitigate the issue caused by sampling undiscovered true positive links as the negative training samples, we trained multiple independent MLP classifiers on different training sets, where the negative links were randomly sampled for each set. We then aggregated their edge classification results to pass to the post-processing step. We defined the following rules from both the computational and biological perspectives to remove unlikely predictions in the post-processing step. Computationally, since there exist two representations for $l_{i,j}$, i.e., $IE_{i,j}$ and $IE_{j,i}$, the prediction for $l_{i,j}$ is defined as a "strong" one if and only if both $IE_{i,j}$ and $IE_{j,i}$ are classified into the same interaction type (excluding the non-interaction type). Biologically, we assumed that the virus S-protein would only bind with its known target receptor.

SUPPLEMENTAL INFORMATION

Supplemental information can be found online at https://doi.org/10.1016/j.patter.2021.100242.

ACKNOWLEDGMENTS

This work was partially supported by NSF OAC 1920147.

AUTHOR CONTRIBUTIONS

Conceptualization, P.H. and H.L.; methodology, H.D., F.C., and H.L.; software, H.D.; formal analysis, H.D.; investigation, F.C. and H.D.; writing – original draft, F.C. and H.D.; writing – review & editing, all authors; visualization, F.C. and H.D.; supervision, H.L. and P.H.

DECLARATION OF INTERESTS

The authors declare no competing interests.

Received: November 20, 2020
Revised: January 6, 2021
Accepted: March 24, 2021
Published: March 29, 2021

REFERENCES

1. Li, Q., Guan, X., Wu, P., Wang, X., Zhou, L., Tong, Y., Ren, R., Kathy, S.M.L., Lau, E.H.Y., Wong, J.Y., et al. (2020). Early transmission dynamics in wuhan, China, of novel coronavirus-infected pneumonia. N. Engl. J. Med. *382*, 1199–1207.
2. World Health Organization WHO Coronavirus (COVID-19) Dashboard. World Health Organization. https://covid19.who.int/. Accessed April, 17, 2020.
3. Sit, T.H.C., Brackman, C.J., Ip, S.M., Tam, K.W.S., Law, P.Y.T., To, E.M.W., Veronica, Y.T. Yu, Sims, L.D., Tsang, D.N.C., Chu, D.K.W., et al. (2020). Infection of dogs with SARS-CoV-2. Nature *586*, 776–778.
4. Halfmann, P.J., Hatta, M., Chiba, S., Maemura, T., Fan, S., Takeda, M., Kinoshita, N., Hattori, S.I., Sakai-Tagawa, Y., Iwatsuki-Horimoto, K., et al. (2020). Transmission of SARS-CoV-2 in domestic cats. N. Engl. J. Med. *383*, 592–594.
5. Wang, L., Mitchell, P.K., Calle, P.P., Bartlett, S.L., McAloose, D., Killian, M.L., Yuan, F., Fang, Y., Goodman, L.B., Fredrickson, R., et al. (2020). Complete genome sequence of SARS-CoV-2 in a tiger from a US zoological collection. Microbiol. Resourc. Announce. *9*, e00468-20.
6. Schlottau, K., Rissmann, M., Graaf, A., Schön, J., Sehl, J., Wylezich, C., Höper, D., Mettenleiter, T.C., Balkema-Buschmann, A., Harder, T., et al. (2020). SARS-CoV-2 in fruit bats, ferrets, pigs, and chickens: an experimental transmission study. Lancet Microbe *1*, e218–e225.
7. Sia, S.F., Yan, L.M., Chin, A.W.H., Fung, K., Choy, K.T., Wong, A.Y.L., Kaewpreedee, P., Perera, R.A.P.M., Poon, L.L.M., Nicholls, J.M., et al. (2020). Pathogenesis and transmission of SARS-CoV-2 in golden hamsters. Nature *583*, 834–838.
8. Munnink, B.B., Sikkema, R.S., Nieuwenhuijse, D.F., Molenaar, R.J., Munger, E., Molenkamp, R., Van Der Spek, A., Tolsma, P., Rietveld, A., Brouwer, M., et al. (2020). Jumping back and forth: anthropozoonotic and zoonotic transmission of SARS-Cov-2 on mink farms. bioRxiv. https://doi.org/10.1101/2020.09.01.277152.
9. Channappanavar, R., Fehr, A.R., Vijay, R., Mack, M., Zhao, J., Meyerholz, D.K., and Perlman, S. (2016). Dysregulated type I interferon and inflammatory monocyte-macrophage responses cause lethal pneumonia in SARS-CoV-infected mice. Cell Host Microbe *19*, 181–193.
10. Matthews, K., Schäfer, A., Pham, A., and Frieman, M. (2014). The SARS coronavirus papain like protease can inhibit IRF3 at a post activation step that requires deubiquitination activity. Virol. J. *11*, 209.
11. Hu, Y., Li, W., Gao, T., Cui, Y., Jin, Y., Li, P., Ma, Q., Liu, X., and Cao, C. (2017). SARS coronavirus nucleocapsid inhibits type I interferon production by interfering with TRIM25-mediated RIG-I ubiquitination. J. Virol. *91*, e02143-16.
12. Chen, X., Yang, X., Zheng, Y., Yang, Y., Xing, Y., and Chen, Z. (2014). SARS coronavirus papain-like protease inhibits the type I interferon signaling pathway through interaction with the STING-TRAF3-TBK1 complex. Protein Cell *5*, 369–381.
13. Li, W., Moore, M.J., Vasilieva, N., Sui, J., Wong, S.K., Berne, M.A., Somasundaran, M., Sullivan, J.L., Luzuriaga, K., Greenough, T.C., et al. (2003). Angiotensin-converting enzyme 2 is a functional receptor for the SARS coronavirus. Nature *426*, 450–454.
14. Lei, Y., Moore, C.B., Liesman, R.M., O'Connor, B.P., Bergstralh, D.T., Chen, Z.J., Pickles, R.J., and Ting, J.P. (2009). MAVS-mediated apoptosis and its inhibition by viral proteins. PloS One *4*, e5466.
15. Haagmans, B.L., Kuiken, T., Martina, B.E., Fouchier, R.A., Rimmelzwaan, G.F., van Amerongen, G., van Riel, D., de Jong, T., Itamura, S., Chan, K.H., et al. (2004). Pegylated interferon-α protects type 1 pneumocytes against SARS coronavirus infection in macaques. Nat. Med. *10*, 290–293.
16. Dahl, H., Linde, A., and Strannegård, O. (2004). In vitro inhibition of SARS virus replication by human interferons. Scand. J. Infect. Dis. *36*, 829–831.
17. Szczepanski, A., Owczarek, K., Bzowska, M., Gula, K., Drebot, I., Ochman, M., Maksym, B., Rajfur, Z., Mitchell, J.A., and Pyrc, K. (2019). Canine respiratory coronavirus, bovine coronavirus, and human coronavirus OC43: receptors and attachment factors. Viruses *11*, 328.
18. Niu, J., Shen, L., Huang, B., Ye, F., Zhao, L., Wang, H., Deng, Y., and Tan, W. (2020). Non-invasive bioluminescence imaging of hCoV-OC43

CellPress
OPEN ACCESS

infection and therapy in the central nervous system of live mice. Antivir. Res. *173*, 104646.

19. Hofmann, H., Pyrc, K., van der Hoek, L., Geier, M., Berkhout, B., and Pöhlmann, S. (2005). Human coronavirus NL63 employs the severe acute respiratory syndrome coronavirus receptor for cellular entry. Proc. Natl. Acad. Sci. U S A *102*, 7988–7993.
20. Haagmans, B.L., Al Dhahiry, S.H., Reusken, C.B., Raj, V.S., Galiano, M., Myers, R., Godeke, G.J., Jonges, M., Farag, E., Diab, A., et al. (2014). Middle east respiratory syndrome coronavirus in dromedary camels: an outbreak investigation. Lancet Infect. Dis. *14*, 140–145.
21. Mou, H., Raj, V.S., van Kuppeveld, F.J., Rottier, P.J., Haagmans, B.L., and Bosch, B.J. (2013). The receptor binding domain of the new Middle East respiratory syndrome coronavirus maps to a 231-residue region in the spike protein that efficiently elicits neutralizing antibodies. J. Virol. *87*, 9379–9383.
22. Kandeil, A., Gomaa, M., Shehata, M., El-Taweel, A., Kayed, A.E., Abiadh, A., Jrijer, J., Moatasim, Y., Kutkat, O., Bagato, O., et al. (2019). Middle East respiratory syndrome coronavirus infection in non-camelid domestic mammals. Emerg. Microbes Infect. *8*, 103–108.
23. Bailey-Elkin, B.A., Knaap, R.C., Johnson, G.G., Dalebout, T.J., Ninaber, D.K., van Kasteren, P.B., Bredenbeek, P.J., Snijder, E.J., Kikkert, M., and Mark, B.L. (2014). Crystal structure of the Middle East respiratory syndrome coronavirus (MERS-CoV) papain-like protease bound to ubiquitin facilitates targeted disruption of deubiquitinating activity to demonstrate its role in innate immune suppression. J. Biol. Chem. *289*, 34667–34682.
24. Lui, P.Y., Wong, L.Y., Fung, C.L., Siu, K.L., Yeung, M.L., Yuen, K.S., Chan, C.P., Woo, P.C., Yuen, K.Y., and Jin, D.Y. (2016). Middle East respiratory syndrome coronavirus m protein suppresses type I interferon expression through the inhibition of TBK1-dependent phosphorylation of IRF3. Emerg. Microbes Infect. *5*, e39.
25. Zhang, H., Penninger, J.M., Li, Y., Zhong, N., and Slutsky, A.S. (2020). Angiotensin-converting enzyme 2 (ACE2) as a SARS-CoV-2 receptor: molecular mechanisms and potential therapeutic target. Intensive Care Med. *46*, 586–590.
26. Shang, J., Ye, G., Shi, K., Wan, Y., Luo, C., Aihara, H., Geng, Q., Auerbach, A., and Li, F. (2020). Structural basis of receptor recognition by SARS-CoV-2. Nature *581*, 221–224.
27. Gordon, D.E., Jang, G.M., Bouhaddou, M., Xu, J., Obernier, K., White, K.M., O'Meara, M.J., Rezelj, V.V., Guo, J.Z., Swaney, D.L., et al. (2020). A SARS-CoV-2 protein interaction map reveals targets for drug repurposing. Nature *583*, 459–468.
28. Messina, F., Giombini, E., Agrati, C., Vairo, F., Ascoli Bartoli, T., Al Moghazi, S., Piacentini, M., Locatelli, F., Kobinger, G., Maeurer, M., et al. (2020). Covid-19: viral-host interactome analyzed by network based-approach model to study pathogenesis of SARS-CoV-2 infection. J. Transl. Med. *18*, 233.
29. Cockrell, A.S., Yount, B.L., Scobey, T., Jensen, K., Douglas, M., Beall, A., Tang, X.C., Marasco, W.A., Heise, M.T., and Baric, R.S. (2016). A mouse model for MERS coronavirus-induced acute respiratory distress syndrome. Nat. Microbiol. *2*, 16226.
30. Gorse, G.J., O'Connor, T.Z., Hall, S.L., Vitale, J.N., and Nichol, K.L. (2009). Human coronavirus and acute respiratory illness in older adults with chronic obstructive pulmonary disease. J. Infect. Dis. *199*, 847–857.
31. Lim, Y.X., Ng, Y.L., Tam, J.P., and Liu, D.X. (2016). Human coronaviruses: a review of virus-host interactions. Diseases *4*, 26.
32. Zhai, X., Sun, J., Yan, Z., Zhang, J., Zhao, J., Zhao, Z., Gao, Q., He, W.T., Veit, M., and Su, S. (2020). Comparison of severe acute respiratory syndrome coronavirus 2 spike protein binding to ACE2 receptors from human, pets, farm animals, and putative intermediate hosts. J. Virol. *94*, e00831–20.
33. Schulz, K.S., and Mossman, K.L. (2016). Viral evasion strategies in type I IFN signaling—a summary of recent developments. Front. Immunol. *7*, 498.
34. Thomas, Sunil (2020). The structure of the membrane protein of SARS-CoV-2 resembles the sugar transporter semisweet. Pathog. Immun. *5*, 342.
35. Siu, K.L., Kok, K.H., Ng, M.J., Poon, V.K.M., Yuen, K.Y., Zheng, B.J., and Jin, D.Y. (2009). Severe acute respiratory syndrome coronavirus m protein inhibits type I interferon production by impeding the formation of TRAF3• TANK• TBK1/IKKε complex. J. Biol. Chem. *284*, 16202–16209.
36. Yuen, C.K., Lam, J.Y., Wong, W.M., Mak, L.F., Wang, X., Chu, H., Cai, J.P., Jin, D.Y., To, K.K., Chan, J.F., Yuen, K.Y., and Kok, K.H. (2020). SARS-CoV-2 nsp13, nsp14, nsp15 and orf6 function as potent interferon antagonists. Emerging Microbes Infect. *9*, 1418–1428.
37. Kopecky-Bromberg, S.A., Martínez-Sobrido, L., Frieman, M., Baric, R.A., and Palese, P. (2007). Severe acute respiratory syndrome coronavirus open reading frame (ORF) 3b, ORF 6, and nucleocapsid proteins function as interferon antagonists. J. Virol. *81*, 548–557.
38. Frieman, M., Yount, B., Heise, M., Kopecky-Bromberg, S.A., Palese, P., and Baric, R.S. (2007). Severe acute respiratory syndrome coronavirus ORF6 antagonizes stat1 function by sequestering nuclear import factors on the rough endoplasmic reticulum/Golgi membrane. J. Virol. *81*, 9812–9824.
39. Wu, B., and Hur, S. (2015). How RIG-I like receptors activate MAVS. Curr. Opin. Virol. *12*, 91–98.
40. Lu, R., Zhao, X., Li, J., Niu, P., Yang, B., Wu, H., Wang, W., Song, H., Huang, B., Zhu, N., et al. (2020). Genomic characterisation and epidemiology of 2019 novel coronavirus: implications for virus origins and receptor binding. Lancet *395*, 565–574.
41. Zhou, P., Yang, X.L., Wang, X.G., Hu, B., Zhang, L., Zhang, W., Si, H.R., Zhu, Y., Li, B., Huang, C.L., et al. (2020). A pneumonia outbreak associated with a new coronavirus of probable bat origin. Nature *579*, 270–273.
42. Pickering, B.S., Smith, G., Pinette, M.M., Embury-Hyatt, C., Moffat, E., Marszal, P., and Lewis, C.E. (2021). Susceptibility of domestic swine to experimental infection with severe acute respiratory syndrome coronavirus 2. Emerging Infect. Dis. *27*, 104.
43. Perozzi, B., Al-Rfou, R., and Skiena, S. (2014). DeepWalk: online learning of social representations. In Proceedings of the 20th ACM SIGKDD International Conference on Knowledge Discovery and Data Mining (KDD '14, ACM), pp. 701–710.
44. Tang, J., Qu, M., Wang, M., Zhang, M., Yan, J., and Mei, Q. (2015). Line: large-scale information network embedding. In Proceedings of the 24th International Conference on World Wide Web (ACM), pp. 1067–1077.
45. Grover, A., and Leskovec, J. (2016). node2vec: scalable feature learning for networks. In Proceedings of the 22nd ACM SIGKDD International Conference on Knowledge Discovery and Data Mining (ACM), pp. 855–864.
46. Wang, D., Cui, P., and Zhu, W. (2016). Structural deep network embedding. In Proceedings of the 22nd ACM SIGKDD International Conference on Knowledge Discovery and Data Mining (KDD '16 ACM), pp. 1225–1234.
47. Jiang, X., Li, P., Li, Y., and Zhen, X. (2019). Graph neural based end-to-end data association framework for online multiple-object tracking. arXiv, 1907.05315.
48. Li, P., Wang, Y., Zhao, H., Hong, P., and Liu, H. (2021). On dyadic fairness: exploring and mitigating bias in graph connections. In Proceedings of International Conference on Learning Representations.
49. Ahmed, A., Shervashidze, N., Narayanamurthy, S., Josifovski, V., and Smola, A.J. (2013). Distributed large-scale natural graph factorization. In Proceedings of the 22nd International Conference on World Wide Web (ACM), pp. 37–48.
50. Zheng, Y., Zhuang, M.W., Han, L., Zhang, J., Nan, M.L., Zhan, P., Kang, D., Liu, X., Gao, C., and Wang, P.H. (2020). Severe acute respiratory syndrome coronavirus 2 (SARS-CoV-2) membrane (M) protein inhibits type I and III interferon production by targeting RIG-I/MDA-5 signaling. Signal Transduct. Targeted Ther. *5*, 299.

51. Mehrabi, N., Morstatter, F., Saxena, N., Lerman, K., and Galstyan, A. (2019). A survey on bias and fairness in machine learning. arXiv, 1908.09635.

52. Hagberg, A., Swart, P., and Chult, D.S. (2008). Exploring network structure, dynamics, and function using networkx. In Proceedings of the 7th Python in Science Conference, G. Varoquaux, T. Vaught, and J. Millman, eds., pp. 11–15, United States.

53. Li, C.K., and Xu, X. (2010). Host immune responses to SARS coronavirus in humans. In Molecular Biology of the SARS-Coronavirus, S.K. Lal, ed. (Springer), pp. 259–278.

54. Totura, A.L., and Baric, R.S. (2012). SARS coronavirus pathogenesis: host innate immune responses and viral antagonism of interferon. Curr. Opin. Virol. *2*, 264–275.

55. Frieman, M., Heise, M., and Baric, R. (2008). SARS coronavirus and innate immunity. Virus Res. *133*, 101–112.

56. Mikolov, T., Chen, K., Corrado, G., and Dean, J. (2013). Efficient estimation of word representations in vector space. arXiv, 1301.3781.

57. Heidarian, A., and Dinneen, M.J. (2016). A hybrid geometric approach for measuring similarity level among documents and document clustering. In Proceedings of the 2016 IEEE Second International Conference on Big Data Computing Service and Applications (BigDataService), pp. 142–151.

58. Pedregosa, F., Varoquaux, G., Gramfort, A., Michel, V., Thirion, B., Grisel, O., Blondel, M., Prettenhofer, P., Weiss, R., Dubourg, V., et al. (2011). Scikit-learn: machine learning in Python. J. Machine Learn. Res. *12*, 2825–2830.

Patterns, Volume *2*

Supplemental information

Network-based virus-host interaction prediction with application to SARS-CoV-2

Hangyu Du, Feng Chen, Hongfu Liu, and Pengyu Hong

This supplementary material contained visualized demonstrations of viral entry in Figure S1 and IFN pathway mechanisms in Figure S2. The full network with predictions made by the model was visualized in three figures: Figure S3 for viral entry, Figure S4 for IFN pathway and Figure S5 for host infection. The full nodes and edges in the network are presented in Table S1 and Table S2. The predicted interactions are presented in Table S3 and Table S4. The PPI data source are shown in Table S5. The IMSP algorithm is presented in Alg. 1.

Supplementary Note 1: Virus Entry - Receptor Binding of S Protein

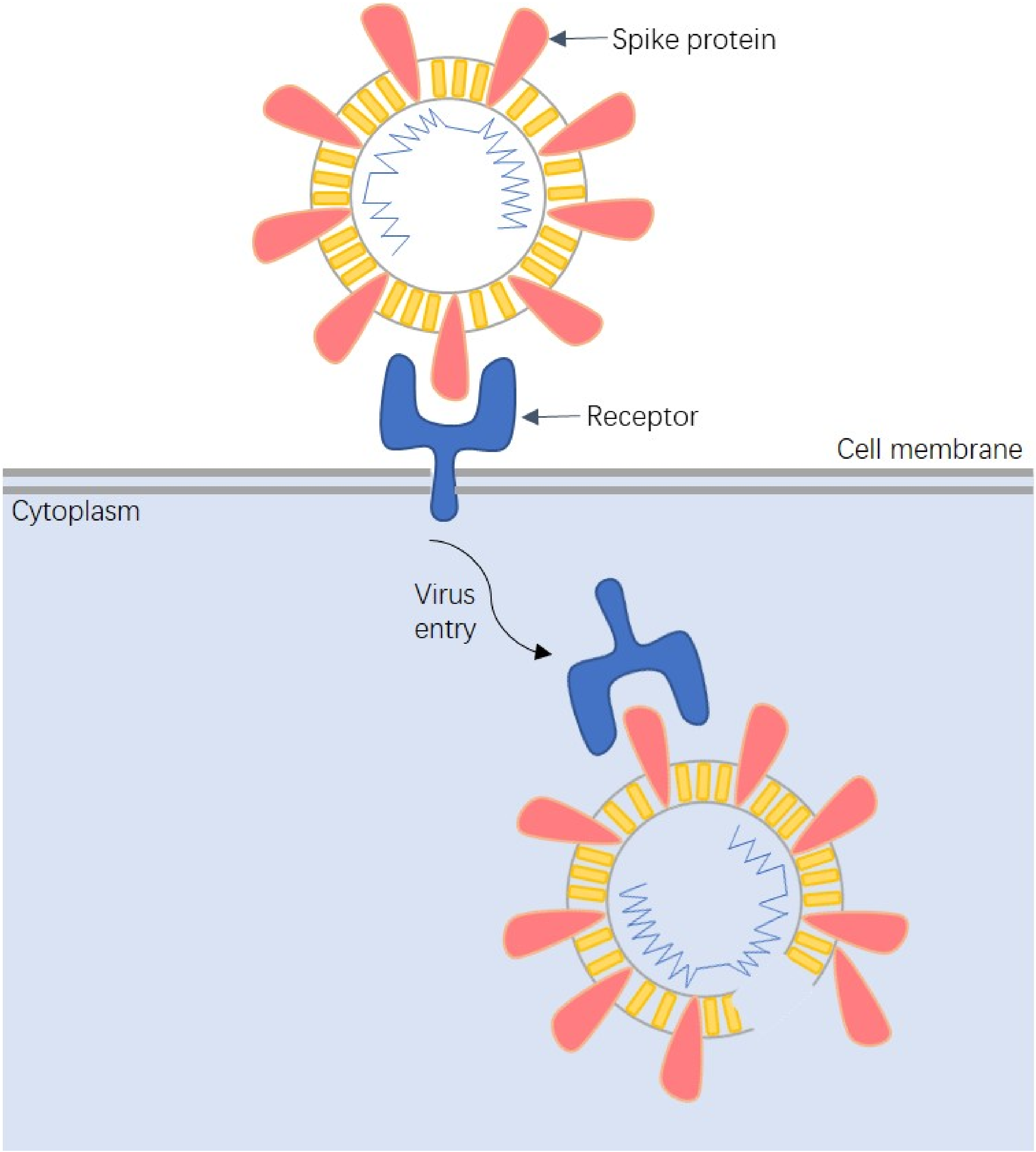

Fig. S1. The Process for Coronavirus Receptor Binding and Virus Entry. *The S protein in coronaviruses plays a crucial role in viral entry. It binds with host receptors and facilitates the fusion between the viral envelope and the host cell membrane.*

Supplementary Note 2: Immune Response - IFN Signaling Pathway

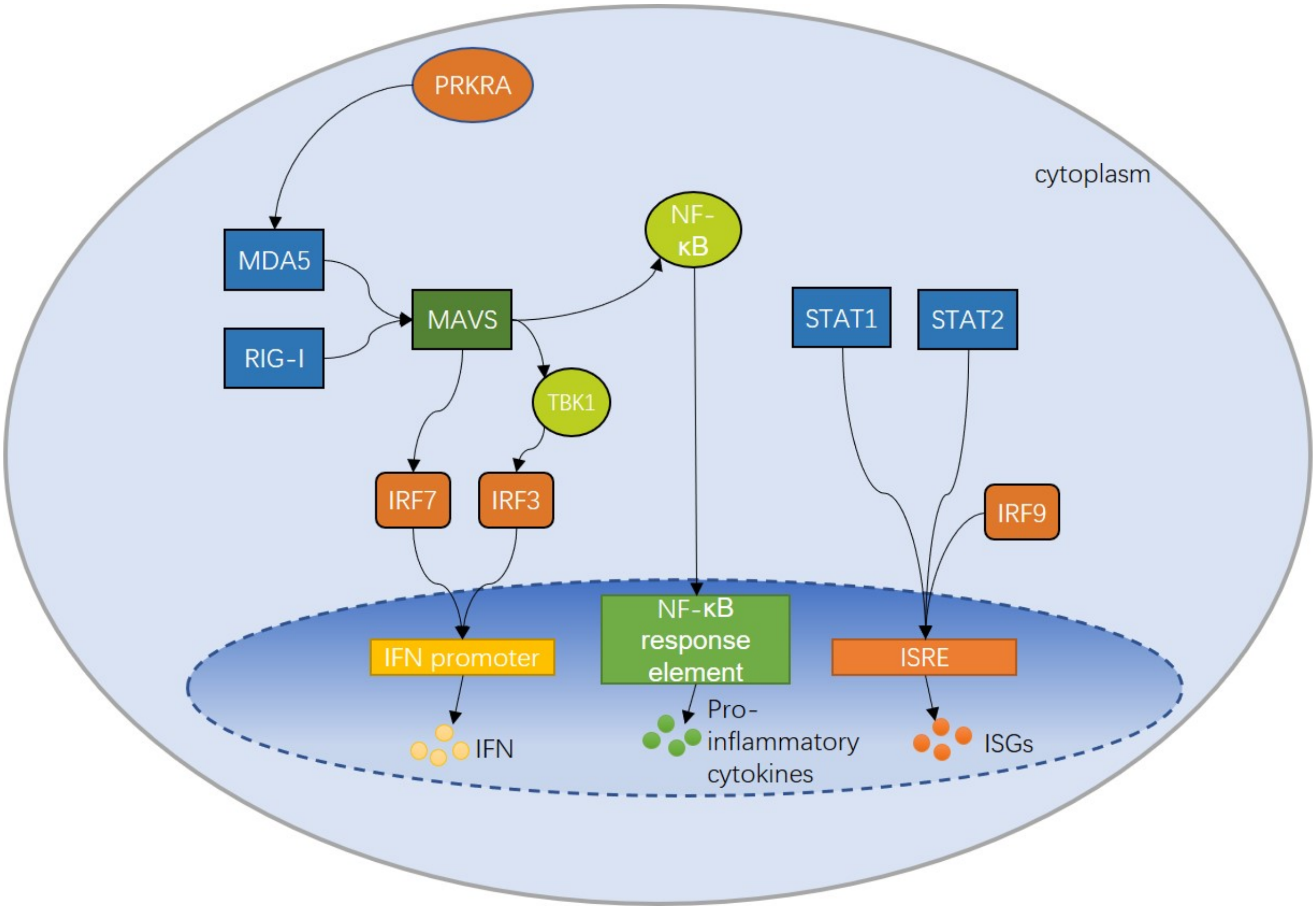

Fig. S2. Innate Immune Response to Coronaviruses' Viral Infection and IFN signaling Mechanism. *RIG-I and MDA5 detect the pattern of virus and trigger the production of Interferons (IFNs)*[S1] *and the activation of the NF-κB.*[S2] *The activated NF-κB induces the Pro-inflammatory cytokines,*[S3] *which play a central role in inflammatory diseases of infectious.*[S4] *STAT1 and STAT2 associate with IRF9 to induce the expression of interferon-stimulated genes (ISGs)*[S5] *and produce antiviral proteins.*[S6] *In this way, viral interactions with the host innate immune system to suppress immune responses become the critical determinant of the disease outcome and viral infection.*

Supplementary Note 3: Full Map of Binding Interactions between Coronaviruses' S Protein and Mammalian Hosts' Receptor

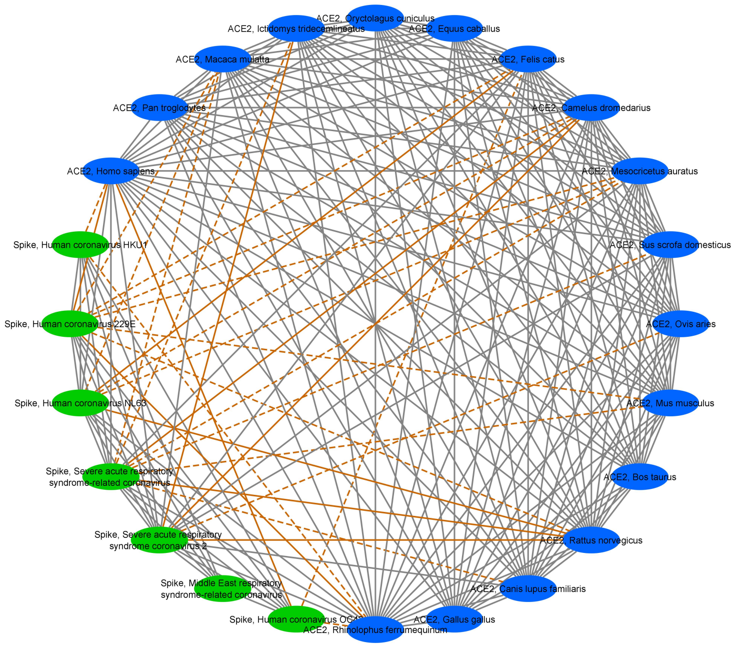

Fig. S3. Virus entry: binding relationships between the S-proteins of human coronaviruses and the ACE2 receptors of mammalian hosts. *This figure of the network is visualized by Cytoscape. Virus Protein nodes are represented in green, and Host Protein Layer nodes are represented in blue. The original interactions are represented in light grey lines, including the known receptor bindings between viruses spike and mammalian hosts ACE2. Predicted receptor bindings are represented in orange lines. As the infection relations have been checked for likelihood in the IMSP model, all predicted interactions are strong predicted interactions.*

Supplementary Note 4: Full Map of PPIs in IFN Signaling Pathway between Coronaviruses' Proteins and Mammalian Hosts' Proteins

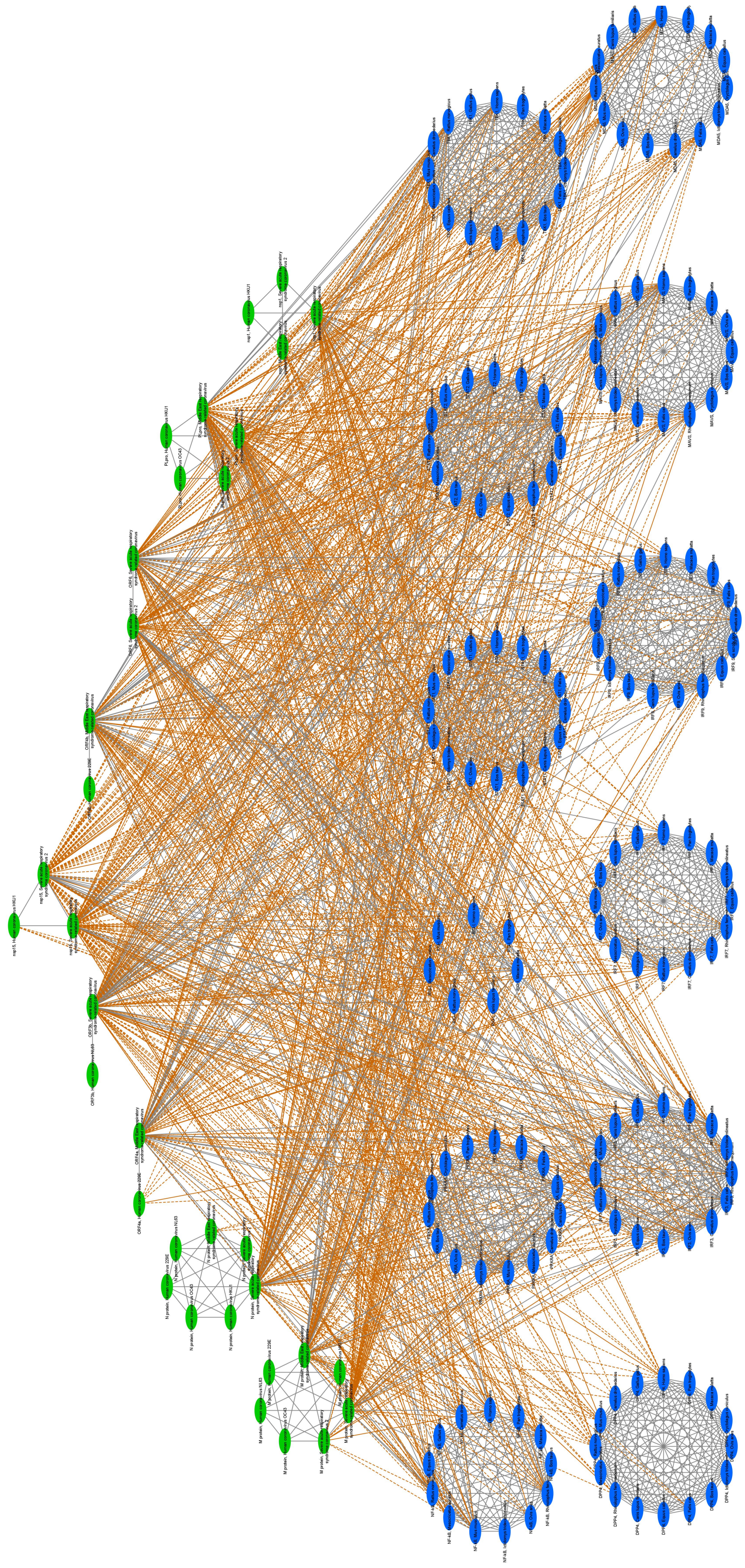

Fig. S4. IFN interactions: virus proteins interactions with IFN signaling pathway to suppress the IFN signaling. *Same representations for nodes and interactions as described in Figure 3. The predicted interactions are represented in orange lines: the solid lines stand for strong predictions, and dotted lines stand for weak predictions as defined in the IMSP model.*

Supplementary Note 5: Full Map of Infection Relationships between Coronaviruses and Mammalian Hosts

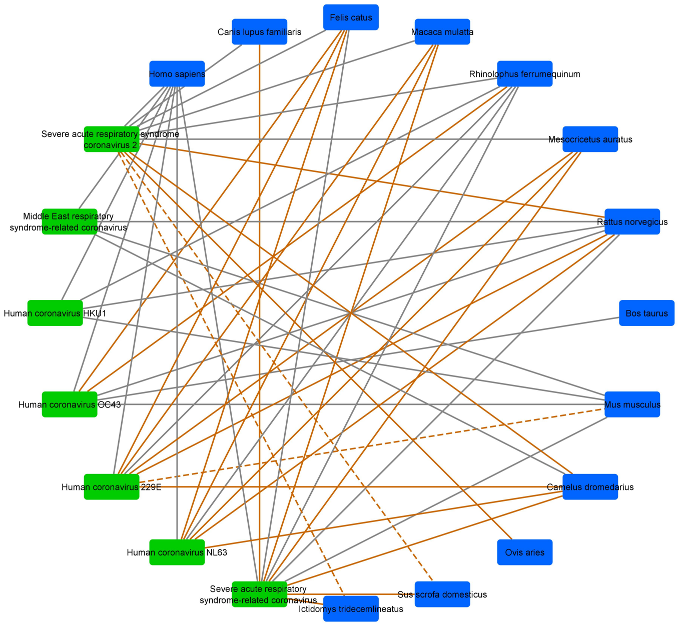

Fig. S5. Coronaviruses and mammalian hosts infection relationships *Virus Layer nodes are represented in green rhombi, and Host Layer nodes are represented in blue. The original infection interactions are represented in grey lines. Predicted infection relations are represented in orange lines: the solid lines stand for strong predictions and dotted lines stand for weak predictions as defined in the IMSP model.*

Supplementary Note 6: Network Node

Table S1. Node IDs and Node Full Names.

Node ID	Full name representation	Node ID	Full name representation
0	nsp15 Severe acute respiratory syndrome coronavirus 2	130	IRF7 Mus musculus
1	nsp15 Severe acute respiratory syndrome-related coronavirus	131	IRF7 Bos taurus
2	nsp15 Human coronavirus HKU1	132	IRF7 Canis lupus familiaris
3	STAT1 Homo sapiens	133	IRF7 Gallus gallus
4	STAT1 Pan troglodytes	134	MDA5 Homo sapiens
5	STAT1 Macaca mulatta	135	MDA5 Pan troglodytes
6	STAT1 Felis catus	136	MDA5 Macaca mulatta
7	STAT1 Camelus dromedarius	137	MDA5 Equus caballus
8	STAT1 Equus caballus	138	MDA5 Oryctolagus cuniculus
9	STAT1 Canis lupus familiaris	139	MDA5 Ictidomys tridecemlineatus
10	STAT1 Rhinolophus ferrumequinum	140	MDA5 Felis catus
11	STAT1 Bos taurus	141	MDA5 Camelus dromedarius
12	STAT1 Ovis aries	142	MDA5 Bos taurus
13	STAT1 Ictidomys tridecemlineatus	143	MDA5 Ovis aries
14	STAT1 Oryctolagus cuniculus	144	MDA5 Mus musculus
15	STAT1 Rattus norvegicus	145	MDA5 Rattus norvegicus
16	STAT1 Mus musculus	146	MDA5 Mesocricetus auratus
17	STAT1 Mesocricetus auratus	147	MDA5 Canis lupus familiaris
18	STAT1 Gallus gallus	148	MDA5 Gallus gallus
19	IRF9 Homo sapiens	149	PRKRA Homo sapiens
20	IRF9 Macaca mulatta	150	PRKRA Macaca mulatta
21	IRF9 Pan troglodytes	151	PRKRA Felis catus
22	IRF9 Felis catus	152	PRKRA Equus caballus
23	IRF9 Camelus dromedarius	153	PRKRA Canis lupus familiaris
24	IRF9 Sus scrofa domesticus	154	PRKRA Camelus dromedarius
25	IRF9 Equus caballus	155	PRKRA Mesocricetus auratus
26	IRF9 Rhinolophus ferrumequinum	156	PRKRA Mus musculus
27	IRF9 Ovis aries	157	PRKRA Ictidomys tridecemlineatus
28	IRF9 Canis lupus familiaris	158	PRKRA Ovis aries
29	IRF9 Bos taurus	159	PRKRA Bos taurus
30	IRF9 Ictidomys tridecemlineatus	160	PRKRA Rattus norvegicus
31	IRF9 Oryctolagus cuniculus	161	PRKRA Rhinolophus ferrumequinum
32	IRF9 Mus musculus	162	PRKRA Oryctolagus cuniculus
33	IRF9 Mesocricetus auratus	163	PRKRA Pan troglodytes
34	IRF9 Rattus norvegicus	164	ORF3b Severe acute respiratory syndrome-related coronavirus
35	IRF9 Gallus gallus	165	ORF3b Human coronavirus NL63
36	RIG-I Homo sapiens	166	DPP4 Homo sapiens
37	RIG-I Pan troglodytes	167	DPP4 Pan troglodytes
38	RIG-I Macaca mulatta	168	DPP4 Macaca mulatta
39	RIG-I Canis lupus familiaris	169	DPP4 Oryctolagus cuniculus
40	RIG-I Rattus norvegicus	170	DPP4 Ovis aries
41	RIG-I Mesocricetus auratus	171	DPP4 Ictidomys tridecemlineatus
42	RIG-I Mus musculus	172	DPP4 Bos taurus
43	ORF4b Middle East respiratory syndrome-related coronavirus	173	DPP4 Felis catus
44	ORF4b Human coronavirus 229E	174	DPP4 Equus caballus
45	nsp1 Severe acute respiratory syndrome coronavirus 2	175	DPP4 Canis lupus familiaris
46	nsp1 Severe acute respiratory syndrome-related coronavirus	176	DPP4 Rhinolophus ferrumequinum
47	nsp1 Middle East respiratory syndrome-related coronavirus	177	DPP4 Mesocricetus auratus
48	nsp1 Human coronavirus HKU1	178	DPP4 Rattus norvegicus
49	Spike Human coronavirus OC43	179	DPP4 Mus musculus
50	Spike Human coronavirus HKU1	180	DPP4 Camelus dromedarius
51	Spike Middle East respiratory syndrome-related coronavirus	181	DPP4 Gallus gallus
52	Spike Severe acute respiratory syndrome coronavirus 2	182	ORF6 Severe acute respiratory syndrome-related coronavirus
53	Spike Severe acute respiratory syndrome-related coronavirus	183	ORF6 Severe acute respiratory syndrome coronavirus 2
54	Spike Human coronavirus NL63	184	STAT2 Homo sapiens
55	Spike Human coronavirus 229E	185	STAT2 Pan troglodytes
56	IRF3 Homo sapiens	186	STAT2 Macaca mulatta
57	IRF3 Pan troglodytes	187	STAT2 Felis catus
58	IRF3 Macaca mulatta	188	STAT2 Canis lupus familiaris
59	IRF3 Ictidomys tridecemlineatus	189	STAT2 Camelus dromedarius
60	IRF3 Rhinolophus ferrumequinum	190	STAT2 Rhinolophus ferrumequinum
61	IRF3 Felis catus	191	STAT2 Equus caballus
62	IRF3 Camelus dromedarius	192	STAT2 Ovis aries
63	IRF3 Ovis aries	193	STAT2 Bos taurus
64	IRF3 Bos taurus	194	STAT2 Mesocricetus auratus
65	IRF3 Equus caballus	195	STAT2 Rattus norvegicus
66	IRF3 Oryctolagus cuniculus	196	STAT2 Ictidomys tridecemlineatus
67	IRF3 Rattus norvegicus	197	STAT2 Mus musculus
68	IRF3 Mesocricetus auratus	198	STAT2 Gallus gallus
69	IRF3 Mus musculus	199	PLpro Middle East respiratory syndrome-related coronavirus
70	IRF3 Canis lupus familiaris	200	PLpro Severe acute respiratory syndrome-related coronavirus
71	IRF3 Gallus gallus	201	PLpro Severe acute respiratory syndrome coronavirus 2
72	N protein Middle East respiratory syndrome-related coronavirus	202	PLpro Human coronavirus OC43
73	N protein Severe acute respiratory syndrome coronavirus 2	203	PLpro Human coronavirus HKU1
74	N protein Severe acute respiratory syndrome-related coronavirus	204	Homo sapiens
75	N protein Human coronavirus HKU1	205	Mus musculus
76	N protein Human coronavirus OC43	206	Rattus norvegicus

Table S1. Node IDs and Node Full Names.

Node ID	Full name representation	Node ID	Full name representation
77	N protein Human coronavirus 229E	207	Canis lupus familiaris
78	N protein Human coronavirus NL63	208	Camelus dromedarius
79	Human coronavirus OC43	209	Felis catus
80	Human coronavirus HKU1	210	Ictidomys tridecemlineatus
81	Middle East respiratory syndrome-related coronavirus	211	Bos taurus
82	Severe acute respiratory syndrome coronavirus 2	212	Pan troglodytes
83	Severe acute respiratory syndrome-related coronavirus	213	Gallus gallus
84	Human coronavirus NL63	214	Oryctolagus cuniculus
85	Human coronavirus 229E	215	Equus caballus
86	ACE2 Homo sapiens	216	Macaca mulatta
87	ACE2 Pan troglodytes	217	Ovis aries
88	ACE2 Macaca mulatta	218	Sus scrofa domesticus
89	ACE2 Ictidomys tridecemlineatus	219	Rhinolophus ferrumequinum
90	ACE2 Oryctolagus cuniculus	220	Mesocricetus auratus
91	ACE2 Equus caballus	221	M protein Middle East respiratory syndrome-related coronavirus
92	ACE2 Felis catus	222	M protein Human coronavirus HKU1
93	ACE2 Camelus dromedarius	223	M protein Severe acute respiratory syndrome-related coronavirus
94	ACE2 Mesocricetus auratus	224	M protein Severe acute respiratory syndrome coronavirus 2
95	ACE2 Sus scrofa domesticus	225	M protein Human coronavirus OC43
96	ACE2 Ovis aries	226	M protein Human coronavirus NL63
97	ACE2 Mus musculus	227	M protein Human coronavirus 229E
98	ACE2 Bos taurus	228	TBK1 Homo sapiens
99	ACE2 Rattus norvegicus	229	TBK1 Pan troglodytes
100	ACE2 Rhinolophus ferrumequinum	230	TBK1 Macaca mulatta
101	ACE2 Canis lupus familiaris	231	TBK1 Oryctolagus cuniculus
102	ACE2 Gallus gallus	232	TBK1 Ictidomys tridecemlineatus
103	ORF4a Middle East respiratory syndrome-related coronavirus	233	TBK1 Felis catus
104	ORF4a Human coronavirus 229E	234	TBK1 Bos taurus
105	NF-κB Homo sapiens	235	TBK1 Rhinolophus ferrumequinum
106	NF-κB Pan troglodytes	236	TBK1 Ovis aries
107	NF-κB Macaca mulatta	237	TBK1 Canis lupus familiaris
108	NF-κB Bos taurus	238	TBK1 Equus caballus
109	NF-κB Rhinolophus ferrumequinum	239	TBK1 Mesocricetus auratus
110	NF-κB Ovis aries	240	TBK1 Mus musculus
111	NF-κB Ictidomys tridecemlineatus	241	TBK1 Camelus dromedarius
112	NF-κB Mus musculus	242	TBK1 Rattus norvegicus
113	NF-κB Mesocricetus auratus	243	TBK1 Gallus gallus
114	NF-κB Rattus norvegicus	244	MAVS Homo sapiens
115	NF-κB Equus caballus	245	MAVS Pan troglodytes
116	NF-κB Gallus gallus	246	MAVS Macaca mulatta
117	NF-κB Camelus dromedarius	247	MAVS Ovis aries
118	IRF7 Homo sapiens	248	MAVS Equus caballus
119	IRF7 Pan troglodytes	249	MAVS Bos taurus
120	IRF7 Macaca mulatta	250	MAVS Oryctolagus cuniculus
121	IRF7 Ictidomys tridecemlineatus	251	MAVS Rhinolophus ferrumequinum
122	IRF7 Equus caballus	252	MAVS Felis catus
123	IRF7 Rhinolophus ferrumequinum	253	MAVS Camelus dromedarius
124	IRF7 Felis catus	254	MAVS Ictidomys tridecemlineatus
125	IRF7 Camelus dromedarius	255	MAVS Canis lupus familiaris
126	IRF7 Rattus norvegicus	256	MAVS Mesocricetus auratus
127	IRF7 Oryctolagus cuniculus	257	MAVS Mus musculus
128	IRF7 Mesocricetus auratus	258	MAVS Rattus norvegicus
129	IRF7 Ovis aries	259	MAVS Gallus gallus

Supplementary Note 7: Edge Table

Table S2. Edge Table with Edge Types

Supplementary Note 8: IMSP Predicted Infections

Certainty is the probability score for the predictions made by IMSP, ranging from 0%-100%. Confidence is the computational rule set in IMSP. Strong confidence represents that for a predicted interaction $E_{i,j}$, its two edge representations $EE_{i,j}$ and $EE_{j,i}$ are all classified into the same class other than the no-interaction class. For weak confidence interactions, only one representation is classified into the class other than the no-interaction class. Likelihood is the biological rule to validate the predictions. Based on pre-defined filters, the unlikely interactions are predictions that have conflicts with those filters.

Table S3. Predicted Infections Table

Source Name	Target Name	Certainty	Confidence	Likelihood
virus Human coronavirus OC43	host Rhinolophus ferrumequinum	96.51%	strong	likely
virus Severe acute respiratory syndrome-related coronavirus	host Macaca mulatta	96.45%	strong	likely
virus Human coronavirus 229E	host Rattus norvegicus	94.14%	strong	likely
virus Severe acute respiratory syndrome-related coronavirus	host Mesocricetus auratus	94.10%	strong	likely
virus Severe acute respiratory syndrome coronavirus 2	host Rattus norvegicus	91.86%	strong	likely
virus Human coronavirus NL63	host Rattus norvegicus	91.15%	strong	likely
virus Severe acute respiratory syndrome-related coronavirus	host Camelus dromedarius	90.80%	strong	likely
virus Human coronavirus 229E	host Camelus dromedarius	87.21%	strong	likely
virus Human coronavirus 229E	host Mesocricetus auratus	85.17%	strong	likely
virus Human coronavirus OC43	host Felis catus	84.30%	strong	likely
virus Severe acute respiratory syndrome-related coronavirus	host Canis lupus familiaris	83.64%	strong	likely
virus Human coronavirus NL63	host Camelus dromedarius	82.39%	strong	likely
virus Human coronavirus 229E	host Macaca mulatta	79.19%	strong	likely
virus Human coronavirus NL63	host Mesocricetus auratus	78.32%	strong	likely
virus Severe acute respiratory syndrome-related coronavirus	host Ictidomys tridecemlineatus	74.45%	strong	likely
virus Human coronavirus 229E	host Felis catus	73.59%	strong	likely
virus Severe acute respiratory syndrome coronavirus 2	host Camelus dromedarius	68.71%	strong	likely
virus Human coronavirus NL63	host Macaca mulatta	68.07%	strong	likely
virus Severe acute respiratory syndrome coronavirus 2	host Ovis aries	65.65%	strong	likely
virus Human coronavirus NL63	host Felis catus	65.62%	strong	likely
virus Severe acute respiratory syndrome-related coronavirus	host Sus scrofa domesticus	62.09%	strong	likely
virus Human coronavirus 229E	host Mus musculus	86.12%	weak	likely
virus Severe acute respiratory syndrome coronavirus 2	host Ictidomys tridecemlineatus	66.26%	weak	likely
virus Severe acute respiratory syndrome coronavirus 2	host Sus scrofa domesticus	59.56%	weak	likely
virus Human coronavirus HKU1	host Mesocricetus auratus	94.84%	strong	unlikely
virus Human coronavirus HKU1	host Camelus dromedarius	94.42%	strong	unlikely
virus Middle East respiratory syndrome-related coronavirus	host Rhinolophus ferrumequinum	93.21%	strong	unlikely
virus Human coronavirus OC43	host Camelus dromedarius	92.94%	strong	unlikely
virus Human coronavirus HKU1	host Macaca mulatta	91.85%	strong	unlikely
virus Human coronavirus OC43	host Mesocricetus auratus	90.58%	strong	unlikely
virus Human coronavirus HKU1	host Felis catus	89.32%	strong	unlikely
virus Middle East respiratory syndrome-related coronavirus	host Macaca mulatta	88.46%	strong	unlikely
virus Severe acute respiratory syndrome-related coronavirus	host Ovis aries	87.95%	strong	unlikely
virus Middle East respiratory syndrome-related coronavirus	host Mesocricetus auratus	84.07%	strong	unlikely
virus Human coronavirus OC43	host Macaca mulatta	83.75%	strong	unlikely
virus Severe acute respiratory syndrome-related coronavirus	host Bos taurus	80.89%	strong	unlikely
virus Human coronavirus HKU1	host Bos taurus	80.83%	strong	unlikely
virus Middle East respiratory syndrome-related coronavirus	host Ovis aries	79.52%	strong	unlikely
virus Human coronavirus HKU1	host Ovis aries	76.10%	strong	unlikely
virus Middle East respiratory syndrome-related coronavirus	host Felis catus	74.00%	strong	unlikely
virus Middle East respiratory syndrome-related coronavirus	host Ictidomys tridecemlineatus	69.40%	strong	unlikely
virus Human coronavirus OC43	host Ovis aries	67.82%	strong	unlikely
virus Human coronavirus HKU1	host Canis lupus familiaris	63.01%	strong	unlikely
virus Human coronavirus OC43	host Sus scrofa domesticus	51.93%	strong	unlikely
virus Human coronavirus 229E	host Ovis aries	47.25%	strong	unlikely
virus Middle East respiratory syndrome-related coronavirus	host Bos taurus	92.83%	weak	unlikely
virus Human coronavirus 229E	host Bos taurus	83.89%	weak	unlikely
virus Human coronavirus NL63	host Mus musculus	82.16%	weak	unlikely
virus Human coronavirus OC43	host Ictidomys tridecemlineatus	81.23%	weak	unlikely
virus Severe acute respiratory syndrome-related coronavirus	host Equus caballus	80.57%	weak	unlikely
virus Middle East respiratory syndrome-related coronavirus	host Canis lupus familiaris	77.79%	weak	unlikely
virus Middle East respiratory syndrome-related coronavirus	host Pan troglodytes	77.02%	weak	unlikely
virus Middle East respiratory syndrome-related coronavirus	host Sus scrofa domesticus	76.57%	weak	unlikely
virus Human coronavirus NL63	host Ovis aries	75.21%	weak	unlikely
virus Human coronavirus NL63	host Bos taurus	74.95%	weak	unlikely
virus Human coronavirus HKU1	host Ictidomys tridecemlineatus	74.01%	weak	unlikely
virus Severe acute respiratory syndrome-related coronavirus	host Pan troglodytes	73.98%	weak	unlikely
virus Human coronavirus HKU1	host Equus caballus	73.25%	weak	unlikely
virus Severe acute respiratory syndrome coronavirus 2	host Bos taurus	69.41%	weak	unlikely
virus Human coronavirus HKU1	host Pan troglodytes	67.38%	weak	unlikely
virus Human coronavirus HKU1	host Sus scrofa domesticus	65.95%	weak	unlikely
virus Human coronavirus OC43	host Gallus gallus	65.57%	weak	unlikely
virus Severe acute respiratory syndrome coronavirus 2	host Equus caballus	63.82%	weak	unlikely
virus Human coronavirus 229E	host Sus scrofa domesticus	62.90%	weak	unlikely
virus Severe acute respiratory syndrome-related coronavirus	host Gallus gallus	62.54%	weak	unlikely
virus Human coronavirus OC43	host Pan troglodytes	61.95%	weak	unlikely
virus Human coronavirus 229E	host Equus caballus	61.42%	weak	unlikely
virus Severe acute respiratory syndrome coronavirus 2	host Pan troglodytes	60.91%	weak	unlikely
virus Human coronavirus 229E	host Canis lupus familiaris	59.83%	weak	unlikely
virus Human coronavirus OC43	host Canis lupus familiaris	58.86%	weak	unlikely
virus Human coronavirus NL63	host Equus caballus	57.11%	weak	unlikely
virus Human coronavirus HKU1	host Gallus gallus	56.75%	weak	unlikely
virus Human coronavirus NL63	host Canis lupus familiaris	56.03%	weak	unlikely

Table S3. Predicted Infections Table

Source Name	Target Name	Certainty	Confidence	Likelihood
virus Human coronavirus 229E	host Ictidomys tridecemlineatus	54.27%	weak	unlikely
virus Human coronavirus OC43	host Equus caballus	53.57%	weak	unlikely
virus Human coronavirus NL63	host Ictidomys tridecemlineatus	52.83%	weak	unlikely
virus Middle East respiratory syndrome-related coronavirus	host Equus caballus	52.31%	weak	unlikely
virus Human coronavirus NL63	host Sus scrofa domesticus	50.30%	weak	unlikely
virus Severe acute respiratory syndrome coronavirus 2	host Gallus gallus	49.51%	weak	unlikely

Supplementary Note 9: IMSP Predicted PPIs

Table S4. Predicted Protein-Protein Interactions Table

Supplementary Note 10: PPIs Data Source

Table S5. PPIs Data Source Table

Virus Protein	Host Protein	Source
SARS-CoV-2 nsp15	IRF3	Yuen CK, Lam JY, Wong WM, et al. SARS-CoV-2 nsp13, nsp14, nsp15 and orf6 function as potent interferon antagonists. Emerg Microbes Infect. 2020;9(1):1418-1428. doi:10.1080/22221751.2020.1780953
SARS-CoV-2 nsp15	RIG-I	Yuen CK, Lam JY, Wong WM, et al. SARS-CoV-2 nsp13, nsp14, nsp15 and orf6 function as potent interferon antagonists. Emerg Microbes Infect. 2020;9(1):1418-1428. doi:10.1080/22221751.2020.1780953
SARS-CoV nsp15	IRF3	Yuen CK, Lam JY, Wong WM, et al. SARS-CoV-2 nsp13, nsp14, nsp15 and orf6 function as potent interferon antagonists. Emerg Microbes Infect. 2020;9(1):1418-1428. doi:10.1080/22221751.2020.1780953
SARS-CoV-2 ORF6	IRF3	Yuen CK, Lam JY, Wong WM, et al. SARS-CoV-2 nsp13, nsp14, nsp15 and orf6 function as potent interferon antagonists. Emerg Microbes Infect. 2020;9(1):1418-1428. doi:10.1080/22221751.2020.1780953
SARS-CoV-2 ORF6	RIG-I	Yuen CK, Lam JY, Wong WM, et al. SARS-CoV-2 nsp13, nsp14, nsp15 and orf6 function as potent interferon antagonists. Emerg Microbes Infect. 2020;9(1):1418-1428. doi:10.1080/22221751.2020.1780953
SARS-CoV nsp15	MAVS	Lei Y, Moore CB, Liesman RM, et al. MAVS-mediated apoptosis and its inhibition by viral proteins. PLoS One. 2009;4(5):e5466. doi:10.1371/journal.pone.0005466
HCoV-HKU1 nsp15	MAVS	Lei Y, Moore CB, Liesman RM, et al. MAVS-mediated apoptosis and its inhibition by viral proteins. PLoS One. 2009;4(5):e5466. doi:10.1371/journal.pone.0005466
SARS-CoV PLpro	TBK1	Siu KL, Kok KH, Ng MH, et al. Severe acute respiratory syndrome coronavirus M protein inhibits type I interferon production by impeding the formation of TRAF3.TANK.TBK1/IKKepsilon complex. J Biol Chem. 2009;284(24):16202-16209. doi:10.1074/jbc.M109.008227
MERS-CoV ORF4b	TBK1	Yang, Y., Ye, F., Zhu, N. et al. Middle East respiratory syndrome coronavirus ORF4b protein inhibits type I interferon production through both cytoplasmic and nuclear targets. Sci Rep 5, 17554 (2015). https://doi.org/10.1038/srep17554
MERS-CoV M protein	TBK1	Lui, P. L., Wong, L. Y. R., Fung, C. L., Siu, K. L., Yeung, M. L., Yuen, K. S., et al. (2016). Middle East respiratory syndrome coronavirus M protein suppresses type I interferon expression through the inhibition of TBK1-dependent phosphorylation of IRF3. Emerg. Microbes Infect. 5:e39. doi: 10.1038/emi.2016.33
MERS-CoV M protein	IRF3	Lui, P. L., Wong, L. Y. R., Fung, C. L., Siu, K. L., Yeung, M. L., Yuen, K. S., et al. (2016). Middle East respiratory syndrome coronavirus M protein suppresses type I interferon expression through the inhibition of TBK1-dependent phosphorylation of IRF3. Emerg. Microbes Infect. 5:e39. doi: 10.1038/emi.2016.33
MERS-CoV PLpro	TBK1	Sun L, Xing Y, Chen X, et al. Coronavirus papain-like proteases negatively regulate antiviral innate immune response through disruption of STING-mediated signaling. PLoS One. 2012;7(2):e30802. doi:10.1371/journal.pone.0030802
MERS-CoV S protein	DPP4	Zhao, J. et al. Rapid generation of a mouse model for Middle East respiratory syndrome. Proc. Natl Acad. Sci. USA 111, 4970–4975 (2014).
MERS-CoV ORF4a	NF-κB	Yang, Y. et al. The structural and accessory proteins M, ORF 4a, ORF 4b, and ORF 5 of Middle East respiratory syndrome coronavirus (MERS-CoV) are potent interferon antagonists. Protein Cell 4, 951–961 (2013).
MERS-CoV ORF4a	IRF3	Yang, Y. et al. The structural and accessory proteins M, ORF 4a, ORF 4b, and ORF 5 of Middle East respiratory syndrome coronavirus (MERS-CoV) are potent interferon antagonists. Protein Cell 4, 951–961 (2013).
MERS-CoV ORF4b	NF-κB	Yang, Y. et al. The structural and accessory proteins M, ORF 4a, ORF 4b, and ORF 5 of Middle East respiratory syndrome coronavirus (MERS-CoV) are potent interferon antagonists. Protein Cell 4, 951–961 (2013).
MERS-CoV ORF4b	IRF3	Yang, Y. et al. The structural and accessory proteins M, ORF 4a, ORF 4b, and ORF 5 of Middle East respiratory syndrome coronavirus (MERS-CoV) are potent interferon antagonists. Protein Cell 4, 951–961 (2013).
MERS-CoV PLpro	NF-κB	Bailey-Elkin, B. A. et al. Crystal structure of the Middle East respiratory syndrome coronavirus (MERS-CoV) papain-like protease bound to ubiquitin facilitates targeted disruption of deubiquitinating activity to demonstrate its role in innate immune suppression. J. Biol. Chem. 289, 34667–34682 (2014).
MERS-CoV PLpro	IRF3	Bailey-Elkin, B. A. et al. Crystal structure of the Middle East respiratory syndrome coronavirus (MERS-CoV) papain-like protease bound to ubiquitin facilitates targeted disruption of deubiquitinating activity to demonstrate its role in innate immune suppression. J. Biol. Chem. 289, 34667–34682 (2014).
SARS-CoV PLpro	NK-κB	Frieman, M., Ratia, K., Johnston, R. E., Mesecar, A. D. & Baric, R. S. Severe acute respiratory syndrome coronavirus papain-like protease ubiquitin-like domain and catalytic domain regulate antagonism of IRF3 and NF-$\varkappa$B signaling. J. Virol. 83, 6689–6705 (2009).
SARS-CoV PLpro	IRF3	Frieman, M., Ratia, K., Johnston, R. E., Mesecar, A. D. & Baric, R. S. Severe acute respiratory syndrome coronavirus papain-like protease ubiquitin-like domain and catalytic domain regulate antagonism of IRF3 and NF-$\varkappa$B signaling. J. Virol. 83, 6689–6705 (2009).
SARS-CoV ORF6	IRF3	Kopecky-Bromberg, S. A., Martinez-Sobrido, L., Frieman, M., Baric, R. A. & Palese, P. Severe acute respiratory syndrome coronavirus open reading frame (ORF) 3b, ORF 6, and nucleocapsid proteins function as interferon antagonists. J. Virol. 81, 548–557 (2007).
SARS-CoV ORF6	IRF9	Emmie de Wit, Neeltje van Doremalen, Darryl Falzarano, and Vincent J Munster. Sars and mers: recentinsights into emerging coronaviruses.Nature reviews. Microbiology, 14(8):523—534, August 2016
SARS-CoV ORF6	STAT1	Kopecky-Bromberg, S. A., Martinez-Sobrido, L., Frieman, M., Baric, R. A. & Palese, P. Severe acute respiratory syndrome coronavirus open reading frame (ORF) 3b, ORF 6, and nucleocapsid proteins function as interferon antagonists. J. Virol. 81, 548–557 (2007).
SARS-CoV ORF6	STAT2	Emmie de Wit, Neeltje van Doremalen, Darryl Falzarano, and Vincent J Munster. Sars and mers: recentinsights into emerging coronaviruses.Nature reviews. Microbiology, 14(8):523—534, August 2016
SARS-CoV ORF3b	STAT2	Emmie de Wit, Neeltje van Doremalen, Darryl Falzarano, and Vincent J Munster. Sars and mers: recentinsights into emerging coronaviruses.Nature reviews. Microbiology, 14(8):523—534, August 2016
SARS-CoV ORF3b	STAT1	Kopecky-Bromberg, S. A., Martinez-Sobrido, L., Frieman, M., Baric, R. A. & Palese, P. Severe acute respiratory syndrome coronavirus open reading frame (ORF) 3b, ORF 6, and nucleocapsid proteins function as interferon antagonists. J. Virol. 81, 548–557 (2007).
SARS-CoV ORF3b	IRF3	Kopecky-Bromberg, S. A., Martinez-Sobrido, L., Frieman, M., Baric, R. A. & Palese, P. Severe acute respiratory syndrome coronavirus open reading frame (ORF) 3b, ORF 6, and nucleocapsid proteins function as interferon antagonists. J. Virol. 81, 548–557 (2007).
SARS-CoV ORF3b	IRF9	Emmie de Wit, Neeltje van Doremalen, Darryl Falzarano, and Vincent J Munster. Sars and mers: recentinsights into emerging coronaviruses.Nature reviews. Microbiology, 14(8):523—534, August 2016
HCoV-NL63 S proten	ACE2	Hofmann H, Pyrc K, van der Hoek L, Geier M, Berkhout B, Pöhlmann S. Human coronavirus NL63 employs the severe acute respiratory syndrome coronavirus receptor for cellular entry. Proc Natl Acad Sci U S A. 2005;102(22):7988-7993. doi:10.1073/pnas.0409465102

Table S5. PPIs Data Source Table

Virus Protein	Host Protein	Source
MERS-CoV M protein	TBK1	Pak-Yin Lui, Lok-Yin Roy Wong, Cheuk-Lai Fung, Kam-Leung Siu, Man-Lung Yeung, Kit-San Yuen, Chi-Ping Chan, Patrick Chiu-Yat Woo, Kwok-Yung Yuen, and Dong-Yan Jin. Mid-dle east respiratory syndrome coronavirus m protein suppresses type i interferon expressionthrough the inhibition of tbk1-dependent phosphorylation of irf3.Emerging microbes & in-fections, 5(1):1–9, 2016
MERS-CoV M protein	STAT1	Emmie de Wit, Neeltje van Doremalen, Darryl Falzarano, and Vincent J Munster. Sars and mers: recentinsights into emerging coronaviruses.Nature reviews. Microbiology, 14(8):523—534, August 2016
MERS-CoV M protein	STAT2	Emmie de Wit, Neeltje van Doremalen, Darryl Falzarano, and Vincent J Munster. Sars and mers: recentinsights into emerging coronaviruses.Nature reviews. Microbiology, 14(8):523—534, August 2016
MERS-CoV M protein	IRF9	Emmie de Wit, Neeltje van Doremalen, Darryl Falzarano, and Vincent J Munster. Sars and mers: recentinsights into emerging coronaviruses.Nature reviews. Microbiology, 14(8):523—534, August 2016
MERS-CoV ORF4a	MDA5	Niemeyer, Daniela et al. "Middle East respiratory syndrome coronavirus accessory protein 4a is a type I interferon antagonist." Journal of virology vol. 87,22 (2013): 12489-95. doi:10.1128/JVI.01845-13
MERS-CoV ORF4a	STAT1	Emmie de Wit, Neeltje van Doremalen, Darryl Falzarano, and Vincent J Munster. Sars and mers: recentinsights into emerging coronaviruses.Nature reviews. Microbiology, 14(8):523—534, August 2016
MERS-CoV ORF4a	IRF9	Emmie de Wit, Neeltje van Doremalen, Darryl Falzarano, and Vincent J Munster. Sars and mers: recentinsights into emerging coronaviruses.Nature reviews. Microbiology, 14(8):523—534, August 2016
MERS-CoV ORF4a	STAT2	Emmie de Wit, Neeltje van Doremalen, Darryl Falzarano, and Vincent J Munster. Sars and mers: recentinsights into emerging coronaviruses.Nature reviews. Microbiology, 14(8):523—534, August 2016
MERS-CoV ORF4b	TBK1	Yang, Y. et al. Middle East respiratory syndrome coronavirus ORF4b protein inhibits type I interferon production through both cytoplasmic and nuclear targets. Sci. Rep. 5, 17554 (2015).
MERS-CoV ORF4b	IRF3	Yang, Y. et al. Middle East respiratory syndrome coronavirus ORF4b protein inhibits type I interferon production through both cytoplasmic and nuclear targets. Sci. Rep. 5, 17554 (2015).
MERS-CoV ORF4b	IRF7	Yang, Y. et al. Middle East respiratory syndrome coronavirus ORF4b protein inhibits type I interferon production through both cytoplasmic and nuclear targets. Sci. Rep. 5, 17554 (2015).
MERS-CoV ORF4b	IRF9	Emmie de Wit, Neeltje van Doremalen, Darryl Falzarano, and Vincent J Munster. Sars and mers: recentinsights into emerging coronaviruses.Nature reviews. Microbiology, 14(8):523—534, August 2016
SARS-CoV nsp1	STAT1	Wathelet, M. G., Orr, M., Frieman, M. B. & Baric, R. S. Severe acute respiratory syndrome coronavirus evades antiviral signaling: role of nsp1 and rational design of an attenuated strain. J. Virol. 81, 11620–11633 (2007).
SARS-CoV nsp1	STAT2	Wathelet, M. G., Orr, M., Frieman, M. B. & Baric, R. S. Severe acute respiratory syndrome coronavirus evades antiviral signaling: role of nsp1 and rational design of an attenuated strain. J. Virol. 81, 11620–11633 (2007).
SARS-CoV ORF3b	MAVS	Emmie de Wit, Neeltje van Doremalen, Darryl Falzarano, and Vincent J Munster. Sars and mers: recentinsights into emerging coronaviruses.Nature reviews. Microbiology, 14(8):523—534, August 2016
SARS-CoV ORF3b	MDA5	Emmie de Wit, Neeltje van Doremalen, Darryl Falzarano, and Vincent J Munster. Sars and mers: recentinsights into emerging coronaviruses.Nature reviews. Microbiology, 14(8):523—534, August 2016
SARS-CoV ORF3b	RIG-I	Emmie de Wit, Neeltje van Doremalen, Darryl Falzarano, and Vincent J Munster. Sars and mers: recentinsights into emerging coronaviruses.Nature reviews. Microbiology, 14(8):523—534, August 2016
SARS-CoV M protein	NF-κB	Fang, Xiaonan et al. "The membrane protein of SARS-CoV suppresses NF-κB activation." Journal of medical virology vol. 79,10 (2007): 1431-9. doi:10.1002/jmv.20953
SARS-CoV M protein	IRF3	Siu KL, Kok KH, Ng MH, et al. Severe acute respiratory syndrome coronavirus M protein inhibits type I interferon production by impeding the formation of TRAF3.TANK.TBK1/IKKepsilon complex. J Biol Chem. 2009;284(24):16202-16209. doi:10.1074/jbc.M109.008227
SARS-CoV N protein	MAVS	Emmie de Wit, Neeltje van Doremalen, Darryl Falzarano, and Vincent J Munster. Sars and mers: recentinsights into emerging coronaviruses.Nature reviews. Microbiology, 14(8):523—534, August 2016
SARS-CoV N protein	MDA5	Emmie de Wit, Neeltje van Doremalen, Darryl Falzarano, and Vincent J Munster. Sars and mers: recentinsights into emerging coronaviruses.Nature reviews. Microbiology, 14(8):523—534, August 2016
SARS-CoV N protein	IRF3	Emmie de Wit, Neeltje van Doremalen, Darryl Falzarano, and Vincent J Munster. Sars and mers: recentinsights into emerging coronaviruses.Nature reviews. Microbiology, 14(8):523—534, August 2016
SARS-CoV N protein	RIG-I	Emmie de Wit, Neeltje van Doremalen, Darryl Falzarano, and Vincent J Munster. Sars and mers: recentinsights into emerging coronaviruses.Nature reviews. Microbiology, 14(8):523—534, August 2016

Supplementary Note 11: IMSP algorithm

Algorithm 1 IMSP algorithm

Input : Pairwise similarity matrices M, interaction relationships R, relevant biological metadata D
Output: Type for each Edge (Interaction) $I_{i,j}$

```
// Stage 1: network construction and representation learning
G = constructNetwork(M, R, D) // construct the unweighted network G
// Text2vec is a Word2vec-based sentence embedding model
Text2vec(V) = Text2vec.train(G.nodes) // Pre-train node content embedding
Text2vec(I) = Text2vec.train(G.pos_and_neg_edges) // Pre-train edge content embedding
for V_i in V do
    R_i^C = Text2vec(V).get(V_i) // Get the content embedding for Node V_i
end
for I_{i,j} in I do
    if V_i and V_j are protein homologs then
        w_{i,j} = getSequenceSimilarity(V_i, V_j) // assign sequence similarity by BLASTp as edge weight
    else
        w_{i,j} = σ(TS-SS_{i,j}/mean(TS-SS)) // assign TS-SS_{i,j} similarity, which takes input R_i^C and R_j^C, as edge
            weight. Refer to Eq.(1)-(4)
    end
end
Node2vec(V) = Node2vec.train(G) // Pre-train structural embeddings on weighted graph G
IE = combine(Node2vec(V), Text2vec(I)) // Generate final edge embeddings. Refer to Eq.(7)
// Stage 2: edge classification
IE_train = getTrainingIE(G) // Get representations for training edges
Label_train = getTrainingLabels(G) // Get labels for training edges
clf = MLP.train(IE_train, Label_train)
return clf.predict() // Return predictions
```

References

[S1] Tomoh Matsumiya and Diana M Stafforini. Function and regulation of retinoic acid-inducible gene-i. *Critical Reviews™ in Immunology*, 30(6), 2010.
[S2] Hilario J Ramos and Michael Gale Jr. Rig-i like receptors and their signaling crosstalk in the regulation of antiviral immunity. *Current opinion in virology*, 1(3):167–176, 2011.
[S3] Ting Liu, Lingyun Zhang, Donghyun Joo, and Shao-Cong Sun. Nf-κb signaling in inflammation. *Signal transduction and targeted therapy*, 2(1):1–9, 2017.
[S4] Laurie Kilpatrick and Mary Catherine Harris. Cytokines and inflammatory response in the fetus and neonate. In *Fetal and neonatal physiology*, pages 1555–1572. Elsevier, 2004.
[S5] Agata Michalska, Katarzyna Blaszczyk, Joanna Wesoly, and Hans AR Bluyssen. A positive feedback amplifier circuit that regulates interferon (ifn)-stimulated gene expression and controls type i and type ii ifn responses. *Frontiers in immunology*, 9:1135, 2018.
[S6] Lionel B Ivashkiv and Laura T Donlin. Regulation of type i interferon responses. *Nature reviews Immunology*, 14(1):36–49, 2014.
